# A database of low-energy atomically precise nanoclusters

**DOI:** 10.1038/s41597-023-02200-4

**Published:** 2023-05-20

**Authors:** Sukriti Manna, Yunzhe Wang, Alberto Hernandez, Peter Lile, Shanping Liu, Tim Mueller

**Affiliations:** Department of Materials Science and Engineering, Johns Hopkins University, Baltimore, MD 21218 USA

**Keywords:** Structural properties, Structure prediction, Computational methods, Nanoparticles

## Abstract

The chemical and structural properties of atomically precise nanoclusters are of great interest in numerous applications, but the structures of the clusters can be computationally expensive to predict. In this work, we present the largest database of cluster structures and properties determined using ab-initio methods to date. We report the methodologies used to discover low-energy clusters as well as the energies, relaxed structures, and physical properties (such as relative stability, HOMO-LUMO gap among others) for 63,015 clusters across 55 elements. We have identified clusters for 593 out of 1595 cluster systems (element-size pairs) explored by literature that have energies lower than those reported in literature by at least 1 meV/atom. We have also identified clusters for 1320 systems for which we were unable to find previous low-energy structures in the literature. Patterns in the data reveal insights into the chemical and structural relationships among the elements at the nanoscale. We describe how the database can be accessed for future studies and the development of nanocluster-based technologies.

## Background & Summary

Small nanoclusters possess novel physical properties which differ from those of their bulk counterparts, including discrete energy levels^1,2^, nonlinear optical properties^3^, magnetism^4^, high catalytic activity^5–7^, multiple absorption bands^7,8^, and enhanced photoluminescence^9–12^. These properties emerge as a consequence of their small sizes and relatively high number of uncoordinated atoms on the surface, and they can be tuned by altering the size and shape of the cluster^13,14^. The past few decades have shown significant progress in computational methods to predict these properties, but before a property can be calculated it is necessary to first determine the atomic structure of the cluster. A number of algorithms have been developed to predict the ground state atomic structure by comprehensively sampling the potential energy surface (PES) of the cluster. These methods include genetic algorithms^15–17^, simulated annealing^18^, particle swarm optimization^19^, Bayesian optimization^20^ and basin-hopping^21^ methods. In each of these methods, identification of the ground state structure is accomplished by first calculating the energies of a large number of candidate structures and then selecting the structures with the lowest energies.

Due in part to strong quantum finite-size effects, accurate determination of the relative cluster energies is best accomplished using ab-initio calculations, and the number of low-energy configurational isomers is estimated to grow exponentially with the number of atoms in the cluster^22,23^. For these reasons searching for the ground state atomic structure can be computationally demanding. An alternative approach to identifying low-energy structures is to search through a reference database of known structures. In recent years, materials databases for crystalline materials have transformed materials research^24–28^. However, current nanocluster structure datasets are either unavailable to the public, limited in scope, or primarily utilize lower levels of theory like interatomic potential models^29–32^ and tight binding models^33^.

Here we present the Quantum Cluster Database (QCD) of low-energy cluster structures for 55 elements, for clusters of 3–55 atoms, calculated using density functional theory (DFT)^34^. The database contains structures obtained through an ab-initio genetic algorithm, an accelerated genetic algorithm using machine-learned interatomic potentials, regression analysis of chemically similar elements, and a survey of the scientific literature. The structures from the scientific literature include 3682 nanoclusters collected or derived from the Cambridge Cluster Database (CCD)^35^, which were identified mostly using interatomic potentials. The 55 elements encompass different regions of the periodic table, including alkali and alkaline earth metals, transition metals, post transition metals, metalloids, and non-metals. Although we are continuing to add to this data set, to the best of our knowledge, this dataset already constitutes the most extensive collection of computed cluster structures at the DFT level of theory. The data set can be used to guide experimental synthesis of predicted nanoclusters, to guide searches for low-energy clusters in different chemical environments, to computationally screen for clusters suitable for a variety of applications, or to train machine learning models. Since the structural energies were obtained using a consistent computational method, the data also serves as a direct source for comparative benchmark studies of different DFT or other electronic structure techniques within the context of atomic cluster modelling. All atomic structures and their calculated properties are openly accessible, enabling researchers across the world to access it for free and use it for further analysis.

## Methods

We have used the following methods to populate the Quantum Cluster Database with atomically precise nanoclusters:

1. We have searched the literature for coordinates of previously discovered candidate low-energy clusters. The atomic structures are available on our QCD website (http://muellergroup.jhu.edu/qcd) and their literature sources are summarized in Table 1 and Fig. 5.

**Table 1:** References of QCD clusters that were extracted from literature.

Element	References	Element	References
*Ag*	^41–47^	*Nb*	^41,42^
*Al*	^36,42,44,46,48–50^	*Ni*	^41–44,47,49,51^
*As*		*Os*	^41,42^
*Au*	^41,42,45,47,52–55^	*P*	^48,56^
*B*	^57–63^	*Pb*	^43–45,49,64–66^
*Ba*	^42–45^	*Pd*	^41,42,67^
*Be*	^68^	*Pt*	^41,42,47^
*Bi*		*Rb*	^43–45,49^
*C*		*Re*	^41,42^
*Ca*	^43,45,49,68^	*Rh*	^41,42,47^
*Cd*	^41,42,69–71^	*Ru*	^41,42^
*Co*	^41,42,72^	*S*	^73^
*Cr*	^41–44,49^	*Sb*	
*Cs*	^42–45,49,74^	*Sc*	^41,42^
*Cu*	^41–43,45,47,49,75^	*Se*	^76^
*Fe*	^41–44,77^	*Si*	^78–86^
*Ga*	^42,87,88^	*Sn*	
*Ge*	^89^	*Sr*	^43–45,49^
*Hf*	^41,42^	*Ta*	^41,42^
*Hg*	^41,42^	*Te*	
*In*	^42^	*Ti*	^41,42^
*Ir*	^41,42^	*Tl*	^42^
*K*	^42–45,49,90^	*V*	^41,42^
*Li*	^91^	*W*	^41–44,49^
*Mg*	^42,48,68,92^	*Y*	^41,42^
*Mn*	^41,42,48^	*Zn*	^41,42,69,71,93^
*Mo*	^41–44,49,77^	*Zr*	^41,42^
*Na*	^42–44,48,49,94^
2. We have used a genetic algorithm with *ab-initio* calculations. This method^36^ was primarily used to identify structures of sizes and elements that are computationally cheap as determined by the number of valence electrons in the projector augmented wave potentials used (Supplementary Table 1), such as *Mg*, *Li*, *Sb*, *Na*, *Ga*, *Si*, *Al*, *B*, *C*, *P* and *S*.
3. We have used a genetic algorithm accelerated by actively learned moment tensor potentials (MTP)^37–40^ trained on-the-fly. This method has been used to search clusters for a few sizes of *Al*^36^, *B*, *C*, *P* and *S*.
4. We have used correlations among energies of same structures but different elements to generate low-energy clusters of elements from known low-energy structures of chemically similar elements.

A brief description of each of these methods is provided below, with additional details in the Supplementary Note 1.

### Low-energy structures mined from existing literature

Many of the clusters in the QCD have been studied before, including systematic DFT studies of small and large clusters across different elements. We collected atomic structures of clusters from publications that provide atomic coordinates of reported low-energy structures, as calculated using DFT, and from the Cambridge Cluster Database, which consists primarily of structures discovered using empirical potentials^36,41–94^. All structures from the literature were relaxed using our DFT settings as described in the section on DFT calculations. In Table 1, we provide the literature references for these cluster structures grouped by element.

### Low-energy structures from *ab initio* genetic algorithm searches

Low-energy cluster structures were also identified by means of a genetic algorithm (GA)^95,96^, an optimization algorithm based on natural evolution in which beneficial characteristics prevail over successive generations. In our implementation^36^, a GA run begins by populating a pool of clusters with random structures and/or seed structures assembled from previous GA runs. All cluster energies are evaluated by relaxing the atomic positions using DFT^34^. Child clusters are generated from pool clusters using one of two types of operations: crossover, in which parts of each parent cluster are combined to form a child cluster, and mutation, in which a subset of atoms of a cluster structure are randomly relocated. If a child cluster has lower energy than the highest-energy pool cluster and is not structurally equivalent to other pool clusters, it replaces the highest-energy cluster of the pool. The cycle continues until the total number of clusters in the GA run is at least 1000. After the search, the 10 lowest-energy clusters (where available) for each element-size pair were collected and added to QCD. Additional details of the genetic algorithm method can be found in SI section 1 as well as the work by Wang *et al*.^36^.

### Learning on the Fly (LOTF)-GA

We have recently developed a way to accelerate the genetic algorithm using machine-learned interatomic potentials trained on-the-fly using active learning^36–38^. The machine-learned interatomic potentials are used to quickly identify candidate low-energy clusters, which are further relaxed locally by DFT to refine the energies. This method has been used to identify low-energy structures for some sizes of *Al*, *B*, *C*, *P* and *S*. Additional details about this method can be found in reference^36^.

### Clusters built from low-energy structures of chemically similar elements

We have constructed additional low-energy cluster structures by taking advantage of the fact that for some elements there are strong correlations between the total energies of chemically similar cluster structures. Low-energy clusters of one element can be used as template to quickly generate low-energy clusters of the positively correlated elements by rescaling the template in proportion to the ratio of nearest neighbor distances. To identify these relationships, we created 55 representative cluster structure prototypes in a two-step process.

In the first step, we used the genetic algorithm to identify low energy structures for clusters of 5, 10, 15 and 20 atoms for *Al*, *Be*, *Li*, *Mg*, *Na*, *Si*, *Ta*, and *Ti*. These elements were chosen because they cover different parts of the periodic table and are computationally inexpensive relative to others because of the small numbers of valence electrons. The low energy configurations are provided in Supplementary Note 5.

**Fig. 1:**
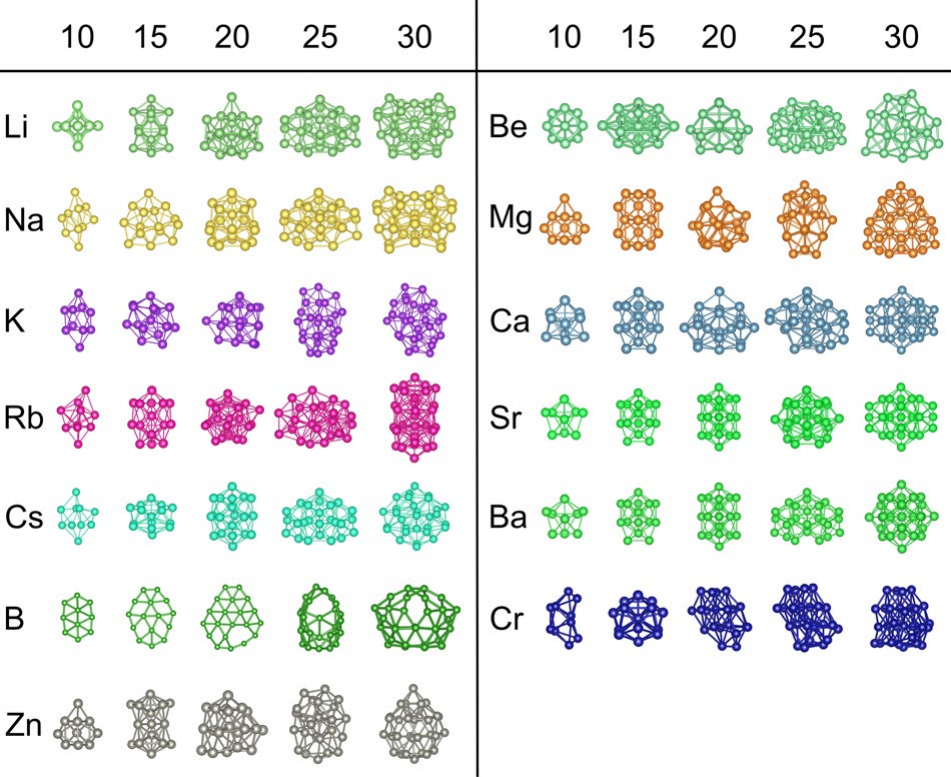
Template clusters used for the data driven method to expedite the filling of the database.

**Fig. 2:**
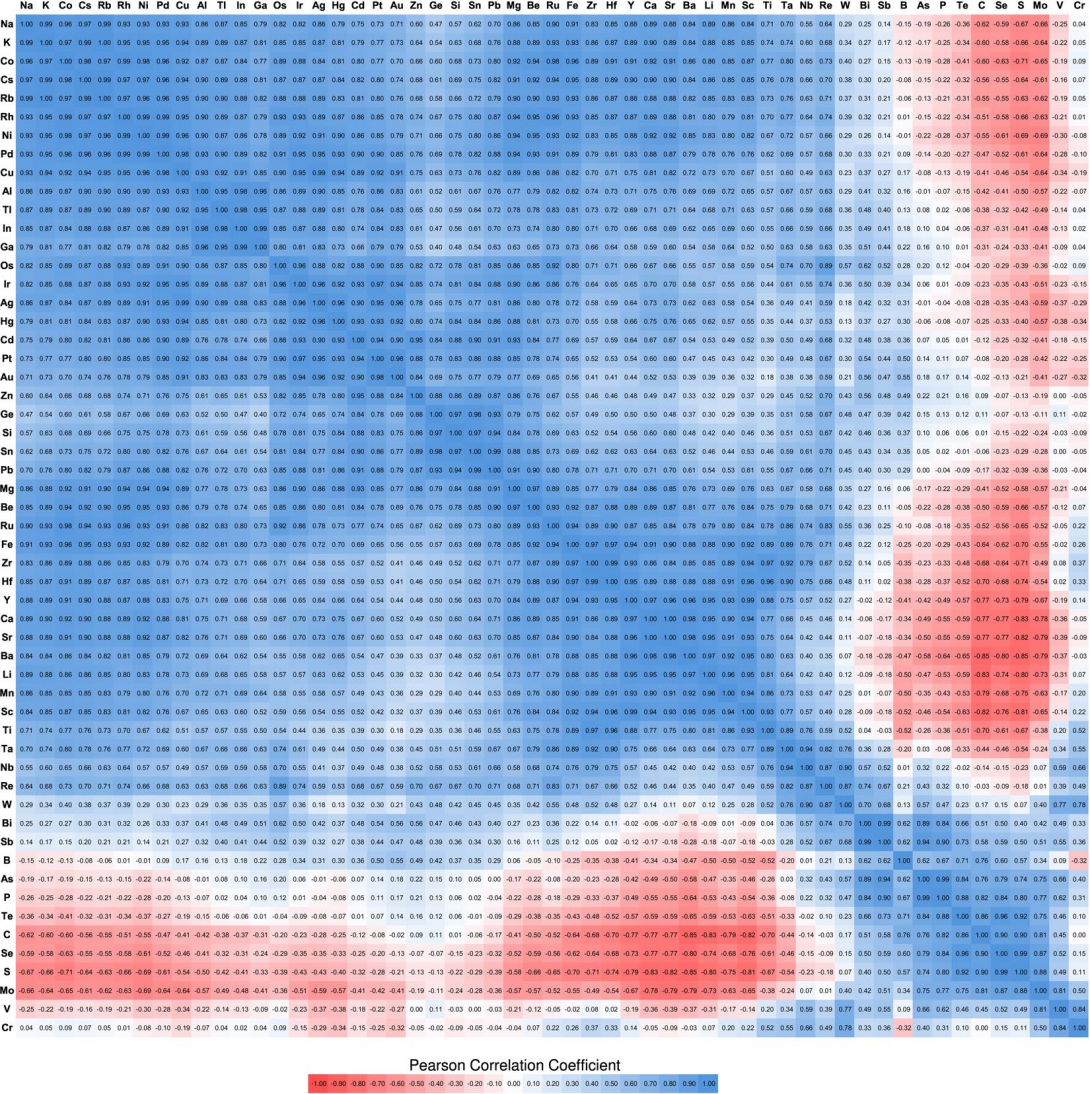
The Pearson correlation coefficients between energies of the set of template clusters of one element with energies of the same set of template clusters of for the rest elements, sorted in the way such that positively correlated elements are close to each other. Blue represents positive correlation, meaning structures having high energies for one element tend to also have high energies for the other element, and structures having low energies for one element tend to also have low energies for the other element. Red represents negative correlation, and white represents no correlation. The correlation values presented in this figure can be downloaded from the header of the QCD website homepage.

In the second step, we used these clusters as templates to create clusters of all the other elements. For each target element, the interatomic distances in the cluster were scaled by the ratio of the nearest neighbor distances of the target element and the template element. The nearest neighbor distances (Supplementary Table 2) are the bond lengths in their most stable bulk form retrieved from the Materials Project^26^. To identify a chemically diverse set of elements, we used least-squares regression to express the DFT-calculated energies of unrelaxed clusters for each element as a linear combination of the energies of the remaining 54 elements. The residual errors for these fits provide a measure of the extent by which each element is different from the other 54 elements. We selected 13 elements with the highest errors: *B*, *Ba*, *Be*, *Ca*, *Cr*, *Cs*, *K*, *Li*, *Mg*, *Na*, *Rb*, *Sr*, and *Zn*, as these are likely to have distinct ground state structures. We then used the genetic algorithm to search for low-energy structures for clusters of 10, 15, 20, 25, and 30 atoms for these 13 elements. The low-energy structures discovered by the genetic algorithm are shown in Fig. 1, and their coordinates are provided in the Supplementary Note 6. These 65 structures were used as structural templates to determine again correlations among energies of different elements, following the same procedure described above. The correlation values are plotted in the heat map of Fig. 2. A positive correlation between a pair of elements means that a cluster structure having high energy for one element also tends to have high energy for the other element, while the negative correlation indicates the reverse relationship.

To evaluate the diversity of the 65 template structures, we compared the clusters using a structural similarity score as described in reference^97^, where perfectly similar structures have a score of 0.0, and we consider structures with a score above 0.3 to be dissimilar. Across the 5 different sizes and 13 different elements, only five pairs have a similarity score less than 0.3, indicating that the remaining pairs of structures are structurally distinct.

After discovering low-energy clusters with the genetic algorithm, we filled gaps on the database (i.e., elements and sizes where no clusters were available) using correlations among the energies of elements (as shown in Fig. 2). For a gap of a given system (an element-size pair), we identified the most correlated element and used its 5 lowest-energy clusters of the same size as templates to generate new clusters that were likely to have low energy. We followed the process of re-scaling the interatomic distances using the bulk nearest-neighbor bond lengths.

### DFT calculations

All DFT local energy minimizations were carried out using the Vienna ab initio Simulation Package^98^ (VASP) with the Perdew-Burke-Ernzerhof^99^ (PBE) generalized gradient approximation exchange-correlation functional. We found that VASP was particularly efficient for clusters with a large number of atoms, which consumed the greatest amount of computational resources. We used the projector-augmented wave^100^ method, with the pseudopotentials and the corresponding default cutoff energies listed in Supplementary Table 1. The convergence criterion for electronic self-consistency was set to 10^−5^ eV per cluster. Structures were optimized using the conjugate gradient algorithm^101,102^ or the RMM-DIIS algorithm^103^ as implemented in VASP until all the atomic forces were less than 0.1 eV/Å. Low-energy clusters within 1 eV from the lowest-energy cluster of each system and all clusters collected from the literature (in total 31,911 clusters) were re-optimized with a tighter force-convergence criterion of 0.025 eV/Å, to increase the accuracy of the low-energy isomers. All calculations were run at the gamma point with spin polarization. The magnetic moments were initialized as 1 µB/atom for non-magnetic elements. For magnetic elements, local magnetic moments were written out for most calculations and the detailed initialization scheme can be found in a separate section below. Gaussian smearing^104^ with σ = 0.0001 eV was used to achieve high accuracy when calculating final energies (2283 clusters out of the total 63,015 clusters used σ = 0.001). To accelerate convergence for some clusters, a two-step minimizations scheme was adopted with smearing of 0.1 eV in the initial step for faster convergence and a smaller value of 0.0001 eV for the final step. Symmetry was turned off for all DFT calculations to increase the chance of completing the calculations successfully. We found the inclusion of spin-orbit coupling (SOC) had little effect on the ranking of low-energy structures. To maintain the consistency of settings of DFT calculations, we did not include SOC-predicted total energies and atomic structures in the QCD database.

All DFT calculations were performed using VASP which can only perform periodic calculations, so each cluster is in effect surrounded by translationally equivalent clusters. Hence it is essential to use a simulation cell that is sufficiently large to avoid interactions among periodic images. For all elements, we enforced that the minimum distance between atoms in periodic images must be greater than 10 angstroms. Additionally, for elements in the Groups 1 A and 2 A of the periodic table, the minimum distance between neighboring images must also be greater than 3.5 times the nearest-neighbor distances listed in Supplementary Table 2. If, after relaxation, the minimum distance between neighboring images shrank below the aforementioned values, we increased the supercell size and ran the DFT calculation again. We found that these “box size” criteria are sufficient to reach energy convergence within 2 meV/atom in all 1135 tested cases (see Supplementary Fig. 7 and Supplementary Note 4 for more details) with a root mean squared error of 0.118 meV/atom.

### Workflow

**Fig. 3:**
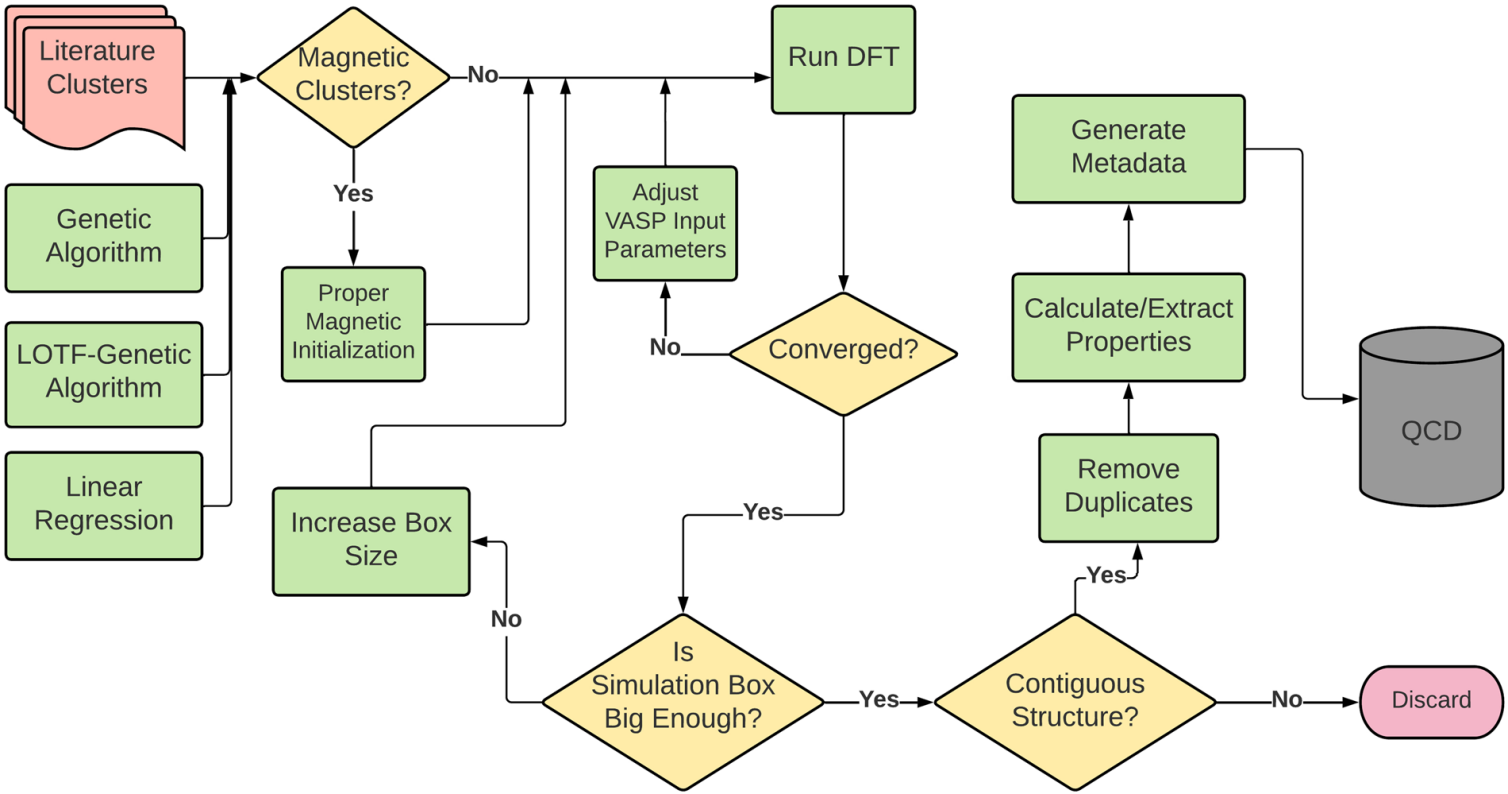
Schematic overview of the high-throughput workflow used in this study.

We identified candidate low-energy cluster structures using one of the four methods listed above. DFT calculations were performed on these clusters structures before adding them to the database. An outline of the high-throughput workflow used in these DFT calculations is provided in Fig. 3. We first initialized calculations of magnetic elements with proper magnetic values (details in next section). Then we ran ionic relaxations and checked convergence. For calculations that did not converge, we adjusted input parameters such as the step size of optimization algorithms and charge mixing parameters and reran them until convergence was achieved.

The atoms sometimes form periodic configurations that correspond to nanowires or slabs. We filtered out these types of structures by discarding clusters that had a minimum distance between periodic images smaller than 1.5 times the atomic nearest neighbor distance. We also screened for discontiguous clusters using this same criterion and discarded any discontiguous clusters that were identified.

To ensure the Quantum Cluster Database contains only unique clusters for a given element and size, when two clusters had a structural similarity score^97^ less than 0.3, the cluster with higher energy was discarded. If the higher-energy cluster was from the literature, the appropriate literature references would be assigned to the structurally similar low-energy cluster. All filters that ensure quality of the clusters in the database are summarized in the Fig. 3.

The properties and metadata described in the Data Records section were calculated for each cluster and stored in a PostgreSQL database. Finally, the data are displayed in the Quantum Cluster Database website (http://muellergroup.jhu.edu/qcd) and output as a JSON file and a CSV file.

### Treatment of magnetic clusters

The final magnetic state of a cluster may depend on the atomic magnetic moments used to initialize the calculation. The final magnetic state is particularly likely to be non-zero for elements with non-zero magnetic moments in their elemental bulk phase, specifically *Fe*, *Mn*, *Co*, *Ni*, *Ru*, *Rh*, *V*, *Cu*, and *Cr*. For elements other than these, we initialized spin-polarized calculations with the default magnetic moment (1 *μ*_*B*_/atom). For the magnetic elements, we performed a benchmark on 2228 clusters with 3 to 55 atoms selected from an early version of QCD and initialized spin-polarized calculations with 4 different magnetic moments, namely 1 *μ*_*B*_/atom, 2 *μ*_*B*_/atom, 3 *μ*_*B*_/atom, and 5 *μ*_*B*_/atom, to evaluate the effect of the initial magnetic moments on the final magnetic states and the total energies. We found that the final magnetic states for *Fe*, *Mn*, *Ru*, *Rh*, *V*, and *Cr* clusters are particularly likely to depend on the initial magnetic moments, whereas for *Cu* and *Co* the initialization with 3 *μ*_*B*_/atom relaxed into the lowest energy configurations in almost all of the benchmarked clusters. For *Ni* clusters, the final states were independent of initialization, so we used the default 1 *μ*_*B*_/atom in the QCD calculations. Supplementary Table 4 showed the effects of different initial magnetic moments on the final magnetic states. To mitigate the chance of missing the correct final magnetic states, multiple initial magnetic moments were used for *Fe*, *Mn*, *Ru*, *Rh*, *V*, and *Cr* clusters, and the calculations yielding the lowest total energies were included in QCD. The set of initial magnetic moments of each element were chosen such that they led to the lowest energy states for more than 97% of all benchmarked clusters of the corresponding element. Table 2 lists the selected set of magnetic moments for the six elements, together with the single initialization value for *Co*, *Cu*, and *Ni*.

**Table 2:** Magnetic initialization schemes for magnetic elements.

Element	Initial Atomic Magnetic Moments (*μ*_*B*_/atom)
*Fe*	+3, +5
*Mn*	+1, +2, +5
*Ru*	+1, +3, +5
*Rh*	+1, +2, +3
*V*	+1, +2, +3
*Cr*	+1, +2, +3, +5
*Co*	+3
*Cu*	+3
*Ni*	+1

## Data Records

We have created a website at http://muellergroup.jhu.edu/qcd to host the database. It provides downloadable links to the correlation table (Fig. 2) and an archive of all relaxed cluster structures, and individual webpages for each cluster with interactive visualization and tabulated cluster properties (discussed in the next section). The input and output files of the DFT calculations of all 63,015 clusters are publicly available (licensed under CC-BY-4.0) in the NOMAD database^105^ at 10.17172/NOMAD/2023.02.01-1^106^. A link is created at the individual cluster webpage on the QCD website connecting to the corresponding data entry in the NOMAD database, where the DFT files can be easily downloaded.

### File format

Properties of all clusters are available for download as a JSON file and as a.csv file on the Quantum Cluster Database website. In the JSON file, each cluster is stored as a key/value pair with “cluster_id” as the key and an object composed of all quantities listed in Table 3 as the value. Within the object, properties of the corresponding cluster are also stored as key/value pairs with keys being those listed in Table 3. The columns of the.csv file correspond to the keys described in Table 3. The input and output VASP files for DFT calculation of each cluster are available in the NOMAD repository at 10.17172/NOMAD/2023.02.01-1.

**Table 3:** Keys, types of data, and description of the QCD data in the JSON file and.csv format.

Key	Datatype	Description
cluster_id	string	ID of the cluster in the QCD
element_symbol	string	Symbol of the element of the cluster
n_atoms	number	Number of atoms in the cluster
n_val_electrons	number	Number of valence electrons corresponding to the pseudopotential
energy_dft	number	Energy in eV
energy_relative	number	Energy in eV above the lowest energy structure of the same element and size
energy_n_minus_one	number	Formation energy in eV relative to the lowest energy structure of the same element but of size N-1
energy_n_plus_one	number	Formation energy in eV relative to the lowest energy structure of the same element but of size N + 1
homo_lumo_gap	number	HOMO-LUMO Gap in eV
magnetic_moment	number	Magnetic moment of the cluster in units of Bohr magneton (µ_B_)
similar_structures	list	Space delimited list of cluster_id of clusters within QCD that are similar to this cluster
references	list	Space delimited list of literature references
structure_xyz	string	Structure represented in XYZ format^*^
structure_poscar_format	string	Structure represented in POSCAR format^*^

*Semicolons are used instead of line breaks.

### Properties

For each cluster of a given number of atoms N and element type k, the database contains the energy relative to the lowest energy structure of size N and species k, the formation energy with respect to the lowest-energy cluster of size N-1 of species k (Eq. (1)), the formation energy with respect to the lowest-energy cluster with N + 1 atoms of the same species (Eq. (2)), the HOMO-LUMO gap, the number of valence electrons considered by DFT, the magnetic moment, a list of similar structures within the Quantum Cluster Database, a list of literature references for the cluster (“http://muellergroup.jhu.edu/qcd” if it was generated by GA or low-energy clusters of chemically similar elements), the coordinates (downloadable in XYZ format and the VASP POSCAR format), and an interactive visualization of the cluster. The formation energies are calculated using the following equations:

1 $${E}_{f,N-1\to N}={E}_{N}-\left({E}_{N-1}+{E}_{atom}\right)$$

2 $${E}_{f,N+1\to N}=\left({E}_{N}+{E}_{atom}\right)-{E}_{N+1}$$

where *E*_N_ is the energy of this cluster of size N, *E*_N-1_ is the energy of the lowest-energy cluster of size N-1, *E*_N+1_ is the energy of the lowest-energy cluster of size N + 1, and *E*_atom_ is the energy of an isolated atom. The energies for isolated atoms used in these calculations are provided in Supplementary Table 3.

The sizes of simulation cells determine the distances between periodic images and can be important for reproducing our results. Therefore, at the structure-view page of each cluster on the QCD website, we provide a link to the relaxed structure in the VASP POSCAR format, which contains the cell lattice vectors and from which the lengths of the simulation cell can be readily calculated.

As a summary, we listed below in Table 4 the links through which readers can access the information discussed in this work.

**Table 4:** Summary of information provided in this work and corresponding links to access it.

Type	Details	Link
Website	Web interface of the Quantum Cluster Database, containing element correlations, properties and visualization of clusters, cluster structures.	http://muellergroup.jhu.edu/qcd
Dataset	DFT calculations of all QCD clusters.	10.17172/NOMAD/2023.02.01-1
Code	Genetic algorithm for generating clusters.	https://gitlab.com/muellergroup/cluster-ga
Code	Management code of QCD.	https://gitlab.com/muellergroup/qcd_mgmt

## Technical Validation

### Comparison of literature clusters and newly reported QCD clusters

**Fig. 4:**
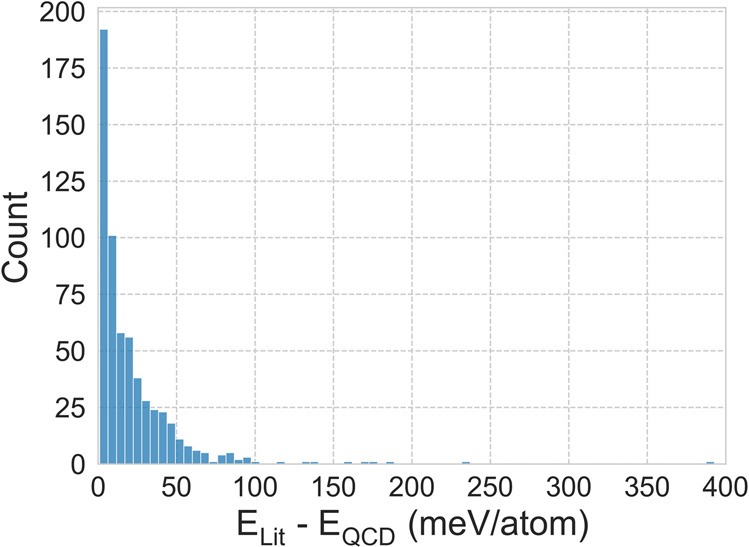
Histogram of energy differences between the lowest-energy clusters reported in the literature (minus 1 meV/atom to account for DFT precision) and the lowest-energy clusters newly reported in the QCD. Out of 1595 systems for which literature have reported structures and provided atomic coordinates, QCD discovered new clusters with lower energies for 593 systems.

For a given cluster size and element, we compared the lowest-energy cluster from the literature against the lowest-energy cluster newly reported in the database to assess which had lower energy. There are 1595 systems for which there is at least one literature structure in the database. Out of those, the database has discovered new lowest-energy clusters for 593 systems that are lower in energy by at least 1 meV/atom (Fig. 4).

**Fig. 5:**
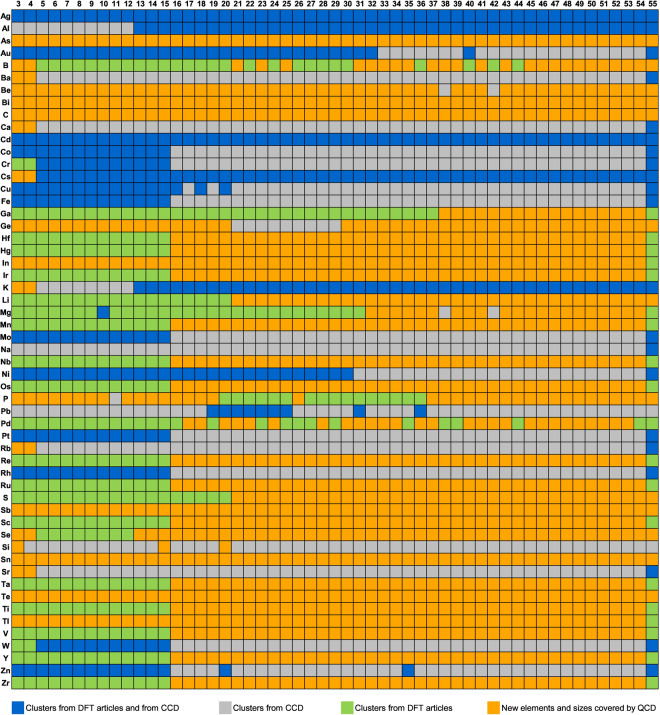
A summary of existing studies of the structures of elemental clusters with 3–55 atoms, including publications that used DFT to find atomic structures (green and blue) as well as the Cambridge Cluster Database (grey and blue) which primarily used interatomic potentials. Together, CCD and literature provide at least one cluster for 1595 systems. The Quantum Cluster Database covers the same cluster systems as well as the remaining 1320 systems that are previously unexplored (orange).

The Quantum Cluster Database contains 1379 structure types or templates (i.e., relative arrangements of atoms) that were not previously reported in the literature (Fig. 5). The 1379 templates were identified from the set of all clusters with calculated energies within 1 meV/atom of the lowest energy cluster with the same element and size. In comparison, there are 582 templates of low-energy clusters from the literature.

Before our work, there were 1595 cluster systems, or approximately 55% of the total 2915 systems, that had at least one structure whose atomic coordinates are available in literature (including the CCD). With the Quantum Cluster Database, the percentage increases to 100%. Table 5 provides a summary of the statistics of cluster systems and total number of clusters from different approaches. We note the sum of the numbers of clusters from different sources does not equal the total number of clusters in the QCD because some clusters are found in multiple sources, as shown in Table 5.

**Table 5:** Statistics of the number of cluster systems and cluster structures from different sources.

Source	Number of cluster systems explored	Total number of clusters included in the QCD*
Cambridge Cluster Database	1355*	3682
Literature	1126*	2574
Genetic algorithm and correlation	2915	57687
Summary of QCD	2915	63015

*Clusters from CCD which are geometrically similar to clusters from literature could also be marked as from literature and vice versa. This makes the sum of cluster systems from these two sources larger than the 1595 systems colored as from CCD or other literature in Fig. 5. Similarly the sum of the numbers of clusters from different sources may not sum to the total number of clusters in the QCD because some clusters may be found in multiple sources.

### Magnetization of magnetic elements

**Fig. 6:**
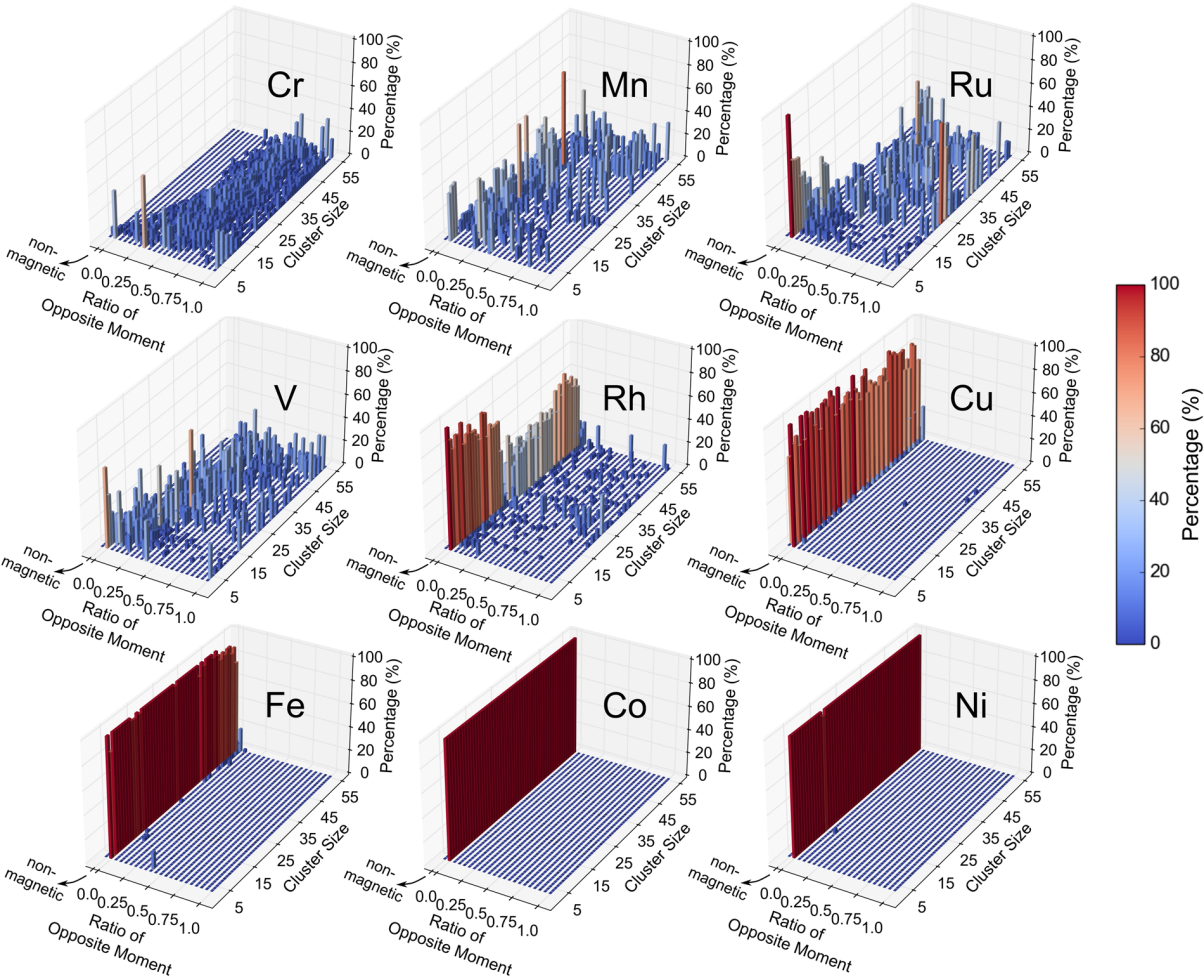
Distribution of the ratio of the sum of opposite local magnetic moments (Eq. (3)) for each cluster size from 3 to 55. If magnetic moments in both spin-up and spin-down directions are 0, it is categorized as non-magnetic. The percentages for each cluster size sum to 1.

We performed a more in-depth analysis on the final magnetic moments of the nine magnetic elements listed above (*Fe*, *Mn*, *Co*, *Ni*, *Ru*, *Rh*, *V*, *Cu*, and *Cr*). We performed our analysis on 12,171 DFT calculations which listed local atomic magnetic moments. We calculated for each cluster the ratio of opposite local magnetic moments:

3 $$r=\min \left(\frac{-{\mu }_{down}}{{\mu }_{up}},\;\frac{-{\mu }_{up}}{{\mu }_{down}}\right),$$

where *μ*_*up*_ is the sum of local magnetic moments with positive values, *μ*_*down*_ represents the sum of local magnetic moments with negative values, and the function min() takes the minimum of the two values. Figure 6 shows the distribution of the ratio of opposite local magnetic moments across the investigated cluster sizes. Although *Cr* is the only antiferromagnetic element in bulk phase at room temperature, we found antiferromagnetic nanoclusters with significant local magnetic moments (≥0.5 *μ*_*B*_ in spin-up and down direction) in five elements, *Cr*, *Mn*, *Ru*, *Rh*, *V*. As the cluster size increases, the lower bound of the opposite moment ratios in Cr gradually increases, indicating more and more spins of *Cr* atoms tend to order antiparallelly. This is likely because there are more high-coordinated atoms as cluster size rises, whose local atomic environments mimic that of the bulk phase. Aside from the antiferromagnetic isomers with the ratio of opposite moments close to 1, there are many clusters with ratios between 0 and 1 for the five elements (*Cr*, *Mn*, *Ru*, *Rh* and *V*), exhibiting states similar to ferrimagnetic configurations, which might result from geometric frustration. Because of the complex geometry of nanoclusters and the lack of a periodic lattice, it is hard for the spins of neighboring atoms to order perfectly in an antiparallel pattern. The ferromagnetic elements *Fe*, *Co*, and *Ni* remain ferromagnetic as atomic clusters. *Cu* mostly alternates between non-magnetic and ferromagnetic with a total magnetic moment of 0 and 1 *μ*_*B*_ for even and odd-sized clusters because of the odd number of valance electrons. For the few cases of *Cu* where the ratio of opposite moments is large, the magnitudes of local moments are very small, suggesting a non-magnetic nature.

### Effect of spin-orbit coupling

**Fig. 7:**
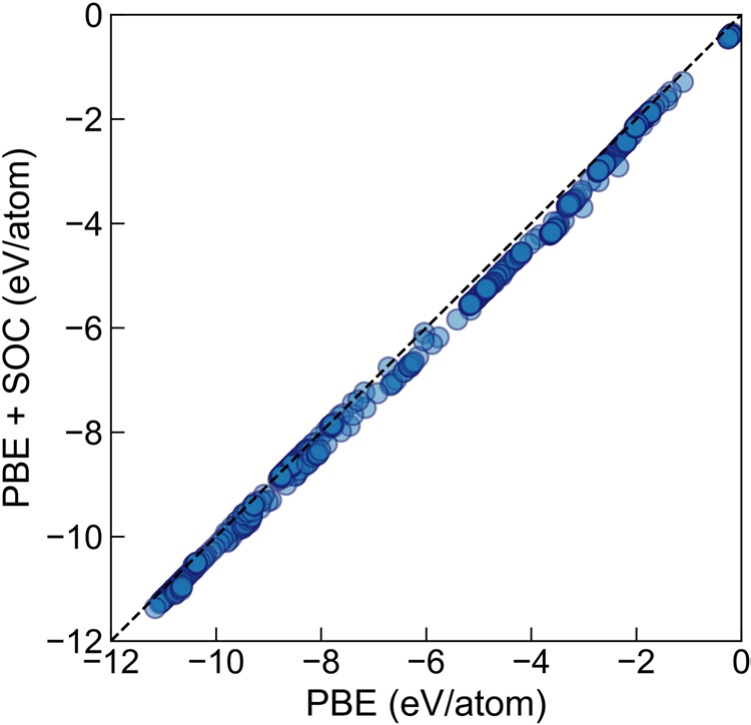
Total energies from PBE calculations vs. energies from PBE + SOC calculations for 11 heavy-metal elements (*Au*, *Bi*, *Hf*, *Hg*, *Ir*, *Os*, *Pb*, *Pt*, *Re*, *Ta*, and *Tl*).

We investigated the effect of spin-orbit coupling (SOC) on heavy-metal elements by performing additional PBE + SOC calculations on the lowest energy clusters of 11 heavy-metal elements, namely *Au*, *Bi*, *Hf*, *Hg*, *Ir*, *Os*, *Pb*, *Pt*, *Re*, *Ta*, and *Tl*, selected based on the work by Piotrowski *et al*.^42^, with sizes ranging from 3 to 55 atoms. Additionally, we choose the six (where available) lowest energy isomers for small (size 10), medium (size 30), and large (size 55) clusters to study the effect of SOC on relative ordering. The energies calculated by PBE + SOC linearly correlated with energies computed by PBE, as shown in Fig. 7. We also developed a cheap proxy to approximate PBE + SOC computed energies from PBE computed energies for these 11 different elements using the least-squares regression. The conversion factors (slope and intercept) for converting to PBE + SOC energies from PBE energies for these elements are provided in Supplementary Fig. 3. We also found that the use of SOC has little effect on the energy rankings of isomers (Supplementary Fig. 4–6). For three small clusters of systems *Pb*_10_, *Tl*_10_ and *Hf*_10_, the PBE + SOC relaxed structures are geometrically dissimilar to the relaxed PBE structures with similarity scores larger than 0.3, and therefore are excluded from the comparisons in Supplementary Fig. 4–6.

## Usage Notes

**Fig. 8:**
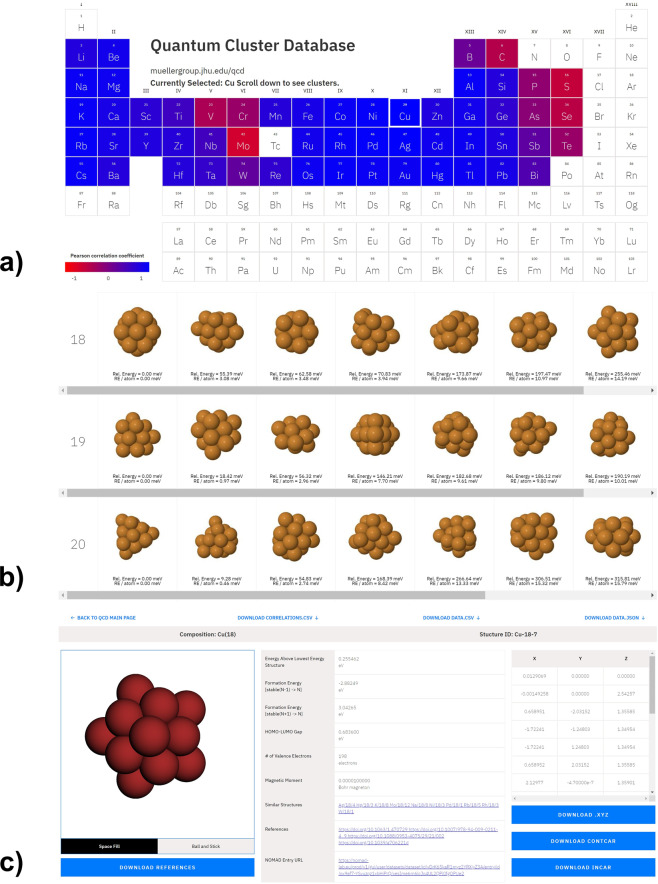
Snapshots of navigating the Quantum Cluster Database. (**a**) Homepage of the Quantum Cluster Database. (**b**) List of clusters displayed after selecting a particular element (*Cu* in the picture). (**c**) Structure view of a selected cluster and its properties.

The homepage of the QCD website (http://muellergroup.jhu.edu/qcd) provides a view of the periodic table and downloadable links to an archive of all relaxed structures and the files summarizing properties of all clusters (in JSON and CSV format). When clicking on one of the 55 elements reported in this work, the other elements will be colored according to the energy correlations (Fig. 8a) and a list of clusters with 3 to 55 atoms of this element will be displayed below the periodic table (Fig. 8b). By default, clusters within 200 meV from the lowest-energy cluster of each size will be displayed. This range can be changed in the input box right beneath the periodic table. For each cluster in the list, a snapshot of the relaxed structure is provided, along with the energy relative to the lowest energy cluster of the corresponding system.

Clicking on a particular cluster from the list brings users to the structure-view page of this specific structure (Fig. 8c). This webpage tabulates the properties of the cluster listed in Table 3 (except for the DFT total energy and the atomic coordinates in POSCAR format). Beside the table, the webpage provides an interactive visualization of the relaxed structure and downloadable links to the structure in XYZ format and POSCAR format of VASP, the VASP input file (INCAR) and a file containing references of this cluster in the BibTex format. The entire set of input and output files of this cluster can also be downloaded from the link to the NOMAD repository at the header of the individual entry page.

## Supplementary information

Supplementary Information

## Data Availability

The implementations of the DFT and MTP genetic algorithms used to search for low-energy structures are available via GitLab: https://gitlab.com/muellergroup/cluster-ga. The scripts and code for managing QCD, for example merging new clusters into QCD, updating existing clusters with DFT calculations using updated parameters, generating metadata of clusters listed in Table 3, are also open-sourced at https://gitlab.com/muellergroup/qcd_mgmt.

## References

[1] Jena P, Sun Q (2018). Super Atomic Clusters: Design Rules and Potential for Building Blocks of Materials. Chem. Rev..

[2] Wilcoxon JP, Abrams BL (2006). Synthesis, structure and properties of metal nanoclusters. Chemical Society Reviews.

[3] Jin R (2015). Atomically precise metal nanoclusters: stable sizes and optical properties. Nanoscale.

[4] Cramer CJ, Truhlar DG (2009). Density functional theory for transition metals and transition metal chemistry. Physical Chemistry Chemical Physics.

[5] Li G, Jin R (2013). Atomically Precise Gold Nanoclusters as New Model Catalysts. Acc. Chem. Res..

[6] Batista KE (2020). Ab Initio Investigation of CO2 Adsorption on 13-Atom 4d Clusters. Journal of chemical information and modeling.

[7] Felício-Sousa P, Andriani KF (2021). & Da Silva, J. L. Ab initio investigation of the role of the d-states occupation on the adsorption properties of H 2, CO, CH 4 and CH 3 OH on the Fe 13, Co 13, Ni 13 and Cu 13 clusters. Physical Chemistry Chemical Physics.

[8] Jia X, Li J, Wang E (2013). Cu Nanoclusters with Aggregation Induced Emission Enhancement. Small.

[9] Zhang Y (2018). Unique size-dependent nanocatalysis revealed at the single atomically precise gold cluster level. Proceedings of the National Academy of Sciences.

[10] Chakraborty I, Pradeep T (2017). Atomically Precise Clusters of Noble Metals: Emerging Link between Atoms and Nanoparticles. Chemical Reviews.

[11] Watanabe Y (2014). Atomically precise cluster catalysis towards quantum controlled catalysts. Science and Technology of Advanced Materials.

[12] Zhu Y, Qian H, Jin R (2011). Catalysis opportunities of atomically precise gold nanoclusters. Journal of Materials Chemistry.

[13] Li ZY (2008). Three-dimensional atomic-scale structure of size-selected gold nanoclusters. Nature.

[14] Castleman AW, Khanna SN (2009). Clusters, Superatoms, and Building Blocks of New Materials. The Journal of Physical Chemistry C.

[15] Wu SQ (2013). An adaptive genetic algorithm for crystal structure prediction. J. Phys.: Condens. Matter.

[16] Curtis F (2018). GAtor: A First-Principles Genetic Algorithm for Molecular Crystal Structure Prediction. Journal of Chemical Theory and Computation.

[17] Jennings PC, Lysgaard S, Hummelshøj JS, Vegge T, Bligaard T (2019). Genetic algorithms for computational materials discovery accelerated by machine learning. npj Computational Materials.

[18] Kirkpatrick S, Gelatt CD, Vecchi MP (1983). Optimization by simulated annealing. science.

[19] Lv J, Wang Y, Zhu L, Ma Y (2012). Particle-swarm structure prediction on clusters. The Journal of Chemical Physics.

[20] Yamashita T (2018). Crystal structure prediction accelerated by Bayesian optimization. Physical Review Materials.

[21] Yang S, Day GM (2021). Exploration and Optimization in Crystal Structure Prediction: Combining Basin Hopping with Quasi-Random Sampling. Journal of Chemical Theory and Computation.

[22] Stillinger FH (1999). Exponential multiplicity of inherent structures. Phys. Rev. E.

[23] Heard, C. J. & Johnston, R. L. in *Challenges and Advances in Computational Chemistry and Physics* Vol. 23 (eds M. Nguyen & B. Kiran) (Springer, Cham, 2017).

[24] Curtarolo S (2012). AFLOW: An automatic framework for high-throughput materials discovery. Computational Materials Science.

[25] Saal JE, Kirklin S, Aykol M, Meredig B, Wolverton C (2013). Materials design and discovery with high-throughput density functional theory: the open quantum materials database (OQMD). Jom.

[26] Jain A (2013). Commentary: The Materials Project: A materials genome approach to accelerating materials innovation. APL Materials.

[27] Choudhary K (2020). The joint automated repository for various integrated simulations (JARVIS) for data-driven materials design. npj Computational Materials.

[28] Zhou J (2019). 2DMatPedia, an open computational database of two-dimensional materials from top-down and bottom-up approaches. Scientific data.

[29] Joswig J-O, Springborg M (2003). Genetic-algorithms search for global minima of aluminum clusters using a Sutton-Chen potential. Physical Review B.

[30] Shao X, Liu X, Cai W (2005). Structural optimization of silver clusters up to 80 atoms with Gupta and Sutton-Chen potentials. Journal of chemical theory and computation.

[31] Grigoryan VG, Alamanova D, Springborg M (2006). Structure and energetics of Cu_*N*_ clusters with (2 ≤ N ≤ 150): An embedded-atom-method study. Phys. Rev. B.

[32] Loeffler TD (2020). Active Learning A Neural Network Model For Gold Clusters & Bulk From Sparse First Principles Training Data. ChemCatChem.

[33] Dong X, Wang GM, Blaisten-Barojas E (2004). Tight-binding model for calcium nanoclusters: Structural, electronic, and dynamical properties. Physical Review B.

[34] Kohn W, Sham L (1965). Phys. Rev. A. Self–Consistent Equations Including Exchange and Correlation Effects.

[35] Wales, D. J. *et al*. The Cambridge Cluster Database https://www-wales.ch.cam.ac.uk/CCD.html (2022).

[36] Wang Y (2022). Accelerated prediction of atomically precise cluster structures using on-the-fly machine learning. npj Comput. Mater..

[37] Shapeev AV (2016). Moment Tensor Potentials: A Class of Systematically Improvable Interatomic Potentials. Multiscale Model. Simul..

[38] Podryabinkin EV, Shapeev AV (2017). Active learning of linearly parametrized interatomic potentials. Computational Materials Science.

[39] Zuo Y (2020). Performance and cost assessment of machine learning interatomic potentials. The Journal of Physical Chemistry A.

[40] Novikov IS, Gubaev K, Podryabinkin EV, Shapeev AV (2020). The MLIP package: moment tensor potentials with MPI and active learning. Machine Learning: Science and Technology.

[41] Chaves AS, Piotrowski MJ, Da Silva JLF (2017). Evolution of the structural, energetic, and electronic properties of the 3d, 4d, and 5d transition-metal clusters (30 TM_n_ systems for n = 2–15): a density functional theory investigation. Physical Chemistry Chemical Physics.

[42] Piotrowski MJ (2016). Theoretical Study of the Structural, Energetic, and Electronic Properties of 55-Atom Metal Nanoclusters: A DFT Investigation within van der Waals Corrections, Spin–Orbit Coupling, and PBE+U of 42 Metal Systems. J. Phys. Chem. C.

[43] Doye JPK, Wales DJ (1997). Structural consequences of the range of the interatomic potential A menagerie of clusters. J. Chem. Soc., Faraday Trans..

[44] Doye JPK, Wales DJ, Berry RS (1995). The effect of the range of the potential on the structures of clusters. J. Chem. Phys..

[45] Wales, D. J. & Doye, J. P. K. in *Large Clusters of Atoms and Molecules* (ed Martin, T. P.) 241–279 (Springer Netherlands, 1996).

[46] Chen M, Dyer JE, Li K, Dixon DA (2013). Prediction of Structures and Atomization Energies of Small Silver Clusters, (Ag)_n_, n <100. J. Phys. Chem. A.

[47] Doye JPK, Wales DJ (1998). Global minima for transition metal clusters described by Sutton–Chen potentials. New Journal of Chemistry.

[48] Galvão BRL, Viegas LP (2019). What Electronic Structure Method Can Be Used in the Global Optimization of Nanoclusters?. J. Phys. Chem. A.

[49] Doye JPK, Wales DJ (1996). The effect of the range of the potential on the structure and stability of simple liquids: from clusters to bulk, from sodium to C60. J. Phys. B: At. Mol. Opt. Phys..

[50] Aguado A, López JM (2009). Structures and stabilities of Al_n_^+^, Al_n_, and Al_n_^−^ (n = 13–34) clusters. J. Chem. Phys..

[51] Song W, Lu W-C, Wang CZ, Ho KM (2011). Magnetic and electronic properties of the nickel clusters Ni_n_ (n ≤ 30). Comput. Theor. Chem..

[52] Liu XJ, Hamilton I, Krawczyk RP, Schwerdtfeger P (2012). The stability of small helical gold nanorods: a relativistic density functional study. J Comput Chem.

[53] Fa W, Luo C, Dong J (2005). Bulk fragment and tubelike structures of Au_N_ (N = 2-26). Phys. Rev. B.

[54] Fernández EM, Soler JM, Garzón IL, Balbás LC (2004). Trends in the structure and bonding of noble metal clusters. Phys. Rev. B.

[55] Zhao H-Y (2010). Structural evolution of Au_n_ (n = 20–32) clusters: Lowest-lying structures and relativistic effects. Phys. Lett. A.

[56] Sai L (2020). Structural Evolution of Medium-Sized Phosphorus Clusters (P20–P36) from Ab Initio Global Search. J. Cluster Sci..

[57] Tai TB, Nguyen MT (2015). Electronic structure and photoelectron spectra of B_n_ with n = 26–29: an overview of structural characteristics and growth mechanism of boron clusters. Physical Chemistry Chemical Physics.

[58] Tai TB, Duong LV, Pham HT, Mai DTT, Nguyen MT (2014). A disk-aromatic bowl cluster B30: toward formation of boron buckyballs. Chem. Commun..

[59] Pham HT, Duong LV, Tam NM, Pham-Ho MP, Nguyen MT (2014). The boron conundrum: Bonding in the bowl B30 and B36, fullerene B40 and triple ring B42 clusters. Chem. Phys. Lett..

[60] Tai TB, Nguyen MT (2016). A new chiral boron cluster B44 containing nonagonal holes. Chem. Commun..

[61] Pham HT, Duong LV, Pham BQ, Nguyen MT (2013). The 2D-to-3D geometry hopping in small boron clusters: The charge effect. Chem. Phys. Lett..

[62] Tai TB, Grant DJ, Nguyen MT, Dixon DA (2010). Thermochemistry and Electronic Structure of Small Boron Clusters (B_n_, n = 5−13) and Their Anions. J. Phys. Chem. A.

[63] Arvanitidis AG, Tai TB, Nguyen MT, Ceulemans A (2014). Quantum rules for planar boron nanoclusters. Physical Chemistry Chemical Physics.

[64] Doye JPK, Hendy SC (2003). On the structure of small lead clusters. The European Physical Journal D - Atomic, Molecular, Optical and Plasma Physics.

[65] Doye JPK (2006). Lead clusters: Different potentials, different structures. Computational Materials Science.

[66] Götz DA, Shayeghi A, Johnston RL, Schwerdtfeger P, Schäfer R (2016). Structural evolution and metallicity of lead clusters. Nanoscale.

[67] Nava P, Sierka M, Ahlrichs R (2003). Density functional study of palladium clusters. Physical Chemistry Chemical Physics.

[68] Dieterich JM, Gerke S, Mata RA (2012). A First-Principles-Based Potential for the Description of Alkaline Earth Metals. Journal of Atomic, Molecular, and Optical Physics.

[69] Doye JPK (2003). Identifying structural patterns in disordered metal clusters. Phys. Rev. B.

[70] Kohaut S, Springborg M (2016). Growth patterns and structural motifs of cadmium clusters with up to 60 atoms: disordered or not?. Physical Chemistry Chemical Physics.

[71] Johansson MP, Pyykkö P (2004). The importance of being tetrahedral: the cadmium pyramids CdN; N = 4, 10, 20, 35 and 56. Physical Chemistry Chemical Physics.

[72] Zhan L, Chen JZY, Liu W-K, Lai SK (2005). Asynchronous multicanonical basin hopping method and its application to cobalt nanoclusters. The Journal of Chemical Physics.

[73] Jin Y (2015). Geometries, stabilities and fragmental channels of neutral and charged sulfur clusters: S_n_^Q^ (n = 3–20, Q = 0, ±1). Physical Chemistry Chemical Physics.

[74] Aguado A (2012). Discovery of Magnetic Superatoms and Assessment of van der Waals Dispersion Effects in Cs_n_ Clusters. J. Phys. Chem. C.

[75] Calaminici P, Pérez-Romero M, Vásquez-Pérez JM, Köster AM (2013). On the ground state structure of neutral Cu_n_ (n = 12,14,16,18,20) clusters. Comput. Theor. Chem..

[76] Alparone A (2012). Density functional theory Raman spectra of cyclic selenium clusters Se_n_ (n = 5–12). Comput. Theor. Chem..

[77] Elliott JA, Shibuta Y, Wales DJ (2009). Global minima of transition metal clusters described by Finnis–Sinclair potentials: A comparison with semi-empirical molecular orbital theory. Philosophical Magazine.

[78] Zhou RL, Pan BC (2007). Structural features of silicon clusters Si_n_ (n = 40–57,60). Phys. Lett. A.

[79] Yoo S, Zeng XC (2006). Structures and relative stability of medium-sized silicon clusters. IV. Motif-based low-lying clusters Si21–Si30. J. Chem. Phys..

[80] Goedecker S, Hellmann W, Lenosky T (2005). Global Minimum Determination of the Born-Oppenheimer Surface within Density Functional Theory. Phys. Rev. Lett..

[81] Yoo S, Shao N, Koehler C, Fraunhaum T, Zeng XC (2006). Structures and relative stability of medium-sized silicon clusters. V. Low-lying endohedral fullerenelike clusters Si31–Si40 and Si45. J. Chem. Phys..

[82] Yoo S, Zeng XC (2005). Motif Transition in Growth Patterns of Small to Medium-Sized Silicon Clusters. Angew. Chem. Int. Ed..

[83] Wang J, Zhou X, Wang G, Zhao J (2005). Optimally stuffed fullerene structures of silicon nanoclusters. Phys. Rev. B.

[84] Yoo S, Zhao J, Wang J, Zeng XC (2004). Endohedral Silicon Fullerenes Si_N_ (27 ≤ N ≤ 39). Journal of the American Chemical Society.

[85] Bazterra VE (2004). Modified genetic algorithms to model cluster structures in medium-size silicon clusters. Phys. Rev. A.

[86] Yoo S, Zeng XC, Zhu X, Bai J (2003). Possible Lowest-Energy Geometry of Silicon Clusters Si21 and Si25. Journal of the American Chemical Society.

[87] Núñez S, López JM, Aguado A (2012). Neutral and charged gallium clusters: structures, physical properties and implications for the melting features. Nanoscale.

[88] Drebov N, Weigend F, Ahlrichs R (2011). Structures and properties of neutral gallium clusters: A theoretical investigation. J. Chem. Phys..

[89] Yoo S, Zeng XC (2006). Search for global-minimum geometries of medium-sized germanium clusters. II. Motif-based low-lying clusters Ge21–Ge29. The Journal of Chemical Physics.

[90] Aguado A (2013). Structures, relative stabilities, and electronic properties of potassium clusters K_n_ (13 ≤ n ≤ 80). Comput. Theor. Chem..

[91] Hu H-S (2016). Theoretical studies of the global minima and polarizabilities of small lithium clusters. Chem. Phys. Lett..

[92] Belyaev SN, Panteleev SV, Ignatov SK, Razuvaev AG (2016). Structural, electronic, thermodynamic and spectral properties of Mg_n_ (n = 2–31) clusters. A DFT study. Comput. Theor. Chem..

[93] Aguado A, Vega A, Lebon A, von Issendorff B (2018). Are zinc clusters really amorphous? A detailed protocol for locating global minimum structures of clusters. Nanoscale.

[94] Noya EG, Doye JPK, Wales DJ, Aguado A (2007). Geometric magic numbers of sodium clusters: Interpretation of the melting behaviour. The European Physical Journal D.

[95] Oganov AR, Lyakhov AO, Valle M (2011). How Evolutionary Crystal Structure Prediction Works—and Why. Acc. Chem. Res..

[96] Trimarchi G, Freeman AJ, Zunger A (2009). Predicting stable stoichiometries of compounds via evolutionary global space-group optimization. Physical Review B.

[97] Li X-T, Yang X-B, Zhao Y-J (2017). Geometrical eigen-subspace framework based molecular conformation representation for efficient structure recognition and comparison. J. Chem. Phys..

[98] Kresse G, Furthmuller J (1996). Efficient iterative schemes for ab initio total-energy calculations using a plane-wave basis set. Phys. Rev. B.

[99] Perdew JP, Burke K, Ernzerhof M (1996). Generalized Gradient Approximation Made Simple. Phys. Rev. Lett..

[100] Blöchl PE (1994). Projector augmented-wave method. Phys. Rev. B.

[101] Gillan M (1989). Calculation of the vacancy formation energy in aluminium. Journal of Physics: Condensed Matter.

[102] Štich I, Car R, Parrinello M, Baroni S (1989). Conjugate gradient minimization of the energy functional: A new method for electronic structure calculation. Physical Review B.

[103] Pulay P (1980). Convergence acceleration of iterative sequences. The case of scf iteration. Chem. Phys. Lett..

[104] Aarons J, Sarwar M, Thompsett D, Skylaris C-K (2016). Perspective: Methods for large-scale density functional calculations on metallic systems. The Journal of chemical physics.

[105] Draxl C, Scheffler M (2018). NOMAD: The FAIR concept for big data-driven materials science. MRS Bull..

[106] Manna S (2022). NOMAD Repository.

