# Supplementary material for: A database of low-energy atomically precise nanoclusters
Source: Sci Data. 2023 May 20;10:308. doi: 10.1038/s41597-023-02200-4 (PMC10199895; doi:10.1038/s41597-023-02200-4)
Supplement: Supplementary file 1 — Supplementary Information [file 41597_2023_2200_MOESM1_ESM.pdf]

*Supporting Information for*

# A database of low-energy atomically precise nanoclusters

Sukriti Manna[1,2], Yunzhe Wang[1,2], Alberto Hernandez[1], Peter Lile[1], Shanping Liu[1] and Tim Mueller[1]

[1]Department of Materials Science and Engineering, Johns Hopkins University, Baltimore, MD 21218

[2]These authors contributed equally

*Corresponding author(s): Tim Mueller (tmueller@jhu.edu)

## Table of Contents

**Supplementary Note 1.** Identifying low energy clusters using genetic algorithm ..... 5

**Supplementary Note 2.** Selection of pseudopotentials ..... 6

**Supplementary Note 3.** DFT calculation strategies for treating magnetic clusters ..... 10

**Supplementary Note 4.** Box size analysis ..... 15

**Supplementary Note 5.** Template Structures Set 1 ..... 17

**Supplementary Note 6.** Template Structures Set 2 ..... 50

## Table of Figures

**Supplementary Figure 1.** A summary of existing studies of the structures of elemental clusters with 3-55 atoms, including publications that used DFT to find atomic structures (green) as well the Cambridge Cluster Database which primarily used interatomic potentials (gray). The overlapping cluster systems are marked blue. The remaining cluster systems, totaling 1320, are those for which no available atomic coordinates were found in literature and are left blank. The detailed literature sources are provided in Table 1 in the main article............................................................ 4
**Supplementary Figure 2.** Schematic workflow of the genetic algorithm used for constructing the Quantum Cluster Database. ............................................................ 5
**Supplementary Figure 3.** PBE + SOC energy conversion from PBE energies using least-square linear regression fit $E_{PBE+SOC} = m * E_{PBE} + b$. The linear parameters (slope $m$ and intercept $b$) along with their RMSE error are provided for each element in the legend of each subplot. ............................................ 11
**Supplementary Figure 4.** Effect of PBE + SOC on relative energy ordering among isomers of *Au*, *Bi*, *Hf*, *Hg*, and *Ir* clusters. Relative energies with respect to the lowest energy isomers are plotted for each polymorph, and the polymorphs are sorted based on the PBE+SOC energies. One $Hf_{10}$ structure was not included in this plot because PBE and PBE+SOC relaxed to dissimilar structures.................................. 12
**Supplementary Figure 5.** Effect of PBE +SOC on relative energy ordering among the isomers of *Os*, *Ta*, *Pb*, *Pt*, and *Re* clusters. Element names and their sizes are stamped in each subplot. Relative energies with respect to lowest energy isomer are plotted against each polymorph which is sorted w.r.t PBE+SOC energies. One $Pb_{10}$ structure was not included in this plot because PBE and PBE+SOC relaxed to dissimilar structures.............................................................. 13
**Supplementary Figure 6.** Effect of PBE +SOC on relative energy ordering among the isomers of *Tl* clusters. Element names and their sizes are stamped in each subplot. Relative energies with respect to lowest energy isomer are plotted against each polymorph which is sorted w.r.t PBE+SOC energies. One $Tl_{10}$ structure was not included in this plot because PBE and PBE-SOC relaxed to dissimilar structures. ....... 14
**Supplementary Figure 7.** Effect of changes in the computational box length from the currently used box length on predicting total energies.............................................................. 15
**Supplementary Figure 8.** The Pearson correlation coefficients for all 55 elements calculated from the energies of relaxed structures, sorted by the mean absolute difference in correlation coefficients calculated from unrelaxed (top) and relaxed (bottom) structures. The data used to generate this figure and Figure 2 of the main text are available as a csv file at the header of the QCD website homepage. ............................. 16

## Table of Tables

**Supplementary Table 1.** Pseudopotentials used in VASP calculations to construct Quantum Cluster Database. ........ 7
**Supplementary Table 2.** List of nearest neighbor distances of all 55 elements used in this work. ........ 8
**Supplementary Table 3.** Energy of an isolated atom for 55 different elements calculated using DFT. ........ 9
**Supplementary Table 4.** Finite magnetic moments after relaxation with default and multiple magnetic initializations per atom using the MAGMOM flag in VASP. This analysis was done by considering clusters of all sizes between 3-55 atoms for each magnetic element. ........ 10

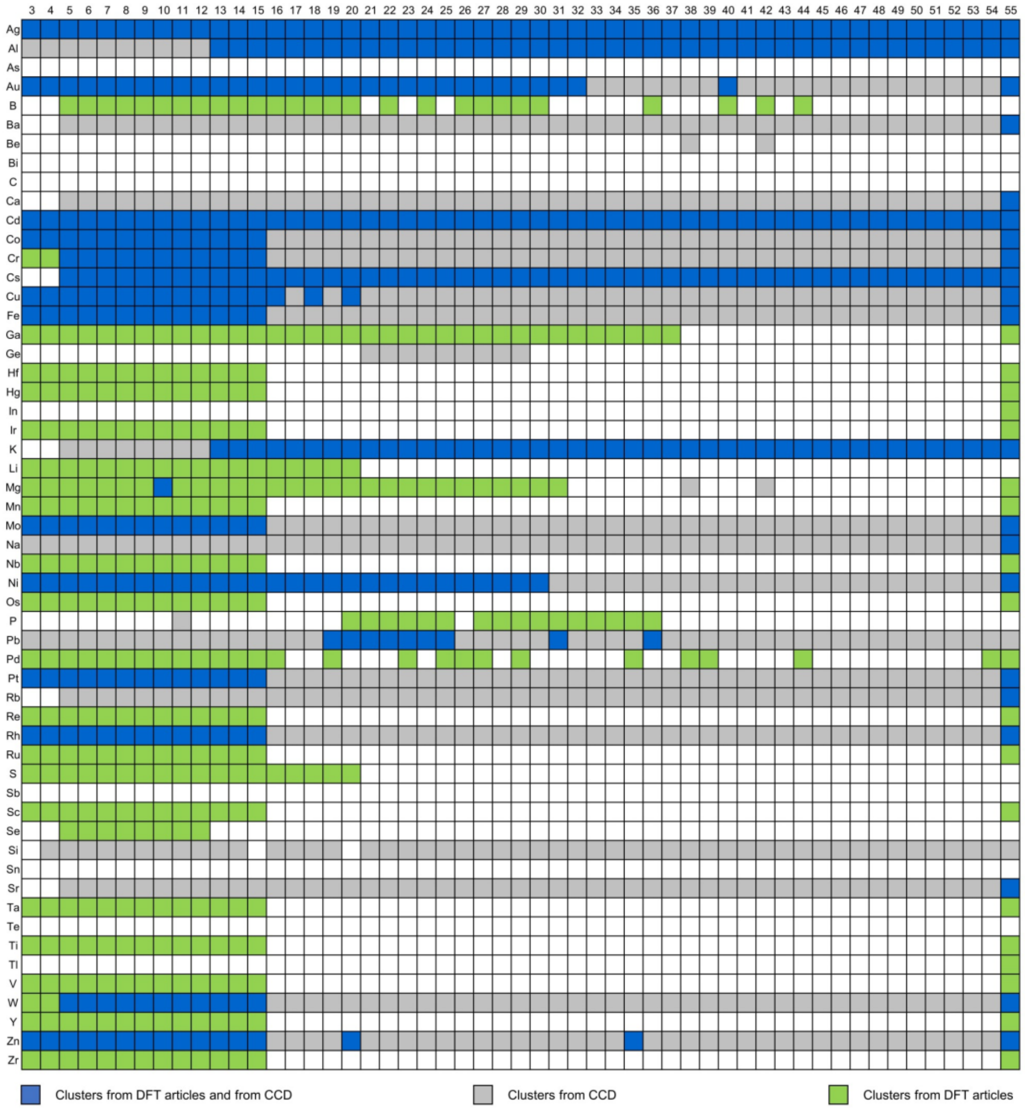


**Supplementary Figure 1.** A summary of existing studies of the structures of elemental clusters with 3-55 atoms, including publications that used DFT to find atomic structures (green) as well the Cambridge Cluster Database which primarily used interatomic potentials (gray). The overlapping cluster systems are marked blue. The remaining cluster systems, totaling 1320, are those for which no available atomic coordinates were found in literature and are left blank. The detailed literature sources are provided in Table 1 in the main article.

## Supplementary Note 1. Identifying low energy clusters using genetic algorithm

Low-energy atomic structures of clusters were identified using a genetic algorithm (GA), a global optimization technique inspired by the principles of natural selection[1]. We implemented our own GA code based off the Birmingham Parallel Genetic Algorithm (BPGA) with some variations[2,3]. Supplementary Figure 2 shows a schematic workflow of the genetic algorithm, details of which are described in our previous work[4].

The majority of GA searches in this work were performed using GA with pure DFT calculations (referred as GA_DFT in our previous work[4]). We evaluated multiple variations of the genetic algorithm as we filled the database. The final parameters and workflow were mostly the same as we reported in reference 4, with a few differences. First, we used a pool size of 10 and stopped the GA once the total number of clusters reached 1000. We also included "seeding" as a genetic operation in GA searches for sulfur clusters, with the ratios of pool clusters generated from seeding, mutation and crossover operations equal to 1:1:3. In the seeding operation, new structures are generated from seed structures which are typically known low-energy structures with different numbers of atoms. Atoms are either randomly added or subtracted from the seed structure until the cluster reaches the target size. Seeding was included for sulfur clusters more than 10 atoms to reduce the chance of clusters being relaxed into discontiguous forms. Low-energy sulfur clusters with N atoms were used to seed searches for clusters with N+1 to N+5 atoms. For large sulfur clusters with 52 and 53 atoms, we initialized them as zig-zag rings, as suggested by the morphology of stable small-size clusters, and then collected the ones that remain contiguous after relaxed by DFT. We accelerated the GA search for sulfur clusters with 51, 52, and 53 atoms with on-the-fly active learning (termed as "GA_AL" in our previous work[4]).

In the energy evaluation by DFT during the GA runs, we set the side length of each simulation box to ensure a distance of at least 10 angstroms between periodic images. For *Na*, *K*, *Rb*, *Cs*, *Mg*, *Sr*, and *Ba*, the distances are increased to at least 3.5 times the nearest neighbor distances because they have larger atomic radii compared with the rest.

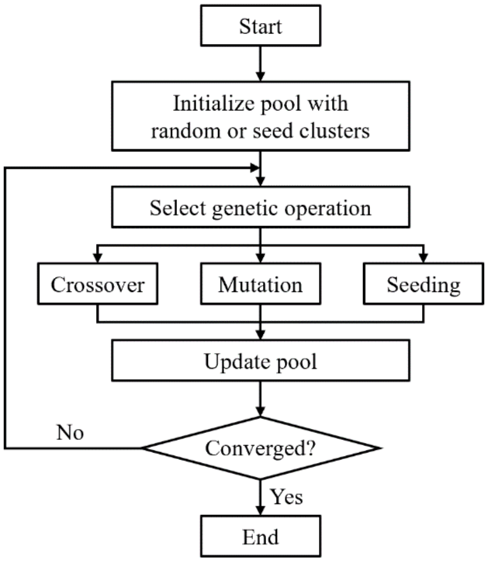


**Supplementary Figure 2.** Schematic workflow of the genetic algorithm used for constructing the Quantum Cluster Database.

## Supplementary Note 2. Selection of pseudopotentials

We chose a set of pseudopotentials that have been widely used and tested by the community for our DFT calculations. The "TITEL" term and ENMAX value from these pseudopotentials distributed by VASP are listed in the Supplementary Table 1 below. To evaluate the impact of the choice of pseudopotentials on our results, we recalculated energies for 3213 clusters of different sizes for 5 randomly chosen benchmark elements, *Ag*, *Be*, *Cd*, *Sc*, and *Ge*, using the newer PBE_GW pseudopotentials. We observe that the shifts in energies from PBE_GW pseudopotentials relative to those from our current choices of PBE pseudopotentials are mostly constant and will not significantly change the relative ordering of clusters in terms of stability.

**Supplementary Table 1.** Pseudopotentials used in VASP calculations to construct Quantum Cluster Database.

| Element | Name ("TITEL" from POTCAR) | ENMAX (eV) | Element | Name ("TITEL" from POTCAR) | ENMAX (eV) |
|---|---|---|---|---|---|
| *Ag* | PAW_PBE Ag 02Apr2005 | 249.844 | *Nb* | PAW_PBE Nb_pv 08Apr2002 | 208.608 |
| *Al* | PAW_PBE Al 04Jan2001 | 240.300 | *Ni* | PAW_PBE Ni 02Aug2007 | 269.532 |
| *As* | PAW_PBE As 22Sep2009 | 208.702 | *Os* | PAW_PBE Os 17Jan2003 | 228.022 |
| *Au* | PAW_PBE Au 04Oct2007 | 229.943 | *P* | PAW_PBE P 06Sep2000 | 255.040 |
| *B* | PAW_PBE B 06Sep2000 | 318.614 | *Pb* | PAW_PBE Pb 08Apr2002 | 97.973 |
| *Ba* | PAW_PBE Ba_sv 06Sep2000 | 187.210 | *Pd* | PAW_PBE Pd 04Jan2005 | 250.925 |
| *Be* | PAW_PBE Be 06Sep2000 | 247.543 | *Pt* | PAW_PBE Pt 04Feb2005 | 230.283 |
| *Bi* | PAW_PBE Bi 08Apr2002 | 105.037 | *Rb* | PAW_PBE Rb_pv 06Sep2000 | 121.882 |
| *C* | PAW_PBE C 08Apr2002 | 400.000 | *Re* | PAW_PBE Re 17Jan2003 | 226.216 |
| *Ca* | PAW_PBE Ca_pv 06Sep2000 | 119.554 | *Rh* | PAW_PBE Rh 04Feb2005 | 228.996 |
| *Cd* | PAW_PBE Cd 06Sep2000 | 274.342 | *Ru* | PAW_PBE Ru 04Feb2005 | 213.271 |
| *Co* | PAW_PBE Co 02Aug2007 | 267.968 | *S* | PAW_PBE S 06Sep2000 | 258.689 |
| *Cr* | PAW_PBE Cr 06Sep2000 | 227.080 | *Sb* | PAW_PBE Sb 06Sep2000 | 172.037 |
| *Cs* | PAW Cs_sv_GW 23Mar2010 | 198.101 | *Sc* | PAW_PBE Sc 04Feb2005 | 154.763 |
| *Cu* | PAW_PBE Cu 22Jun2005 | 295.446 | *Se* | PAW_PBE Se 06Sep2000 | 211.534 |
| *Fe* | PAW_PBE Fe 06Sep2000 | 267.883 | *Si* | PAW_PBE Si 05Jan2001 | 245.345 |
| *Ga* | PAW_PBE Ga 08Apr2002 | 134.678 | *Sn* | PAW_PBE Sn 08Apr2002 | 103.236 |
| *Ge* | PAW_PBE Ge 05Jan2001 | 173.807 | *Sr* | PAW_PBE Sr_sv 07Sep2000 | 229.282 |
| *Hf* | PAW_PBE Hf 20Jan2003 | 220.333 | *Ta* | PAW_PBE Ta 17Jan2003 | 223.667 |
| *Hg* | PAW_PBE Hg 06Sep2000 | 233.214 | *Te* | PAW_PBE Te 08Apr2002 | 174.982 |
| *In* | PAW_PBE In 08Apr2002 | 95.934 | *Ti* | PAW_PBE Ti 08Apr2002 | 178.330 |
| *Ir* | PAW_PBE Ir 06Sep2000 | 210.870 | *Tl* | PAW_PBE Tl 08Apr2002 | 90.140 |
| *K* | PAW_PBE K_pv 17Jan2003 | 116.731 | *V* | PAW_PBE V 08Apr2002 | 192.543 |
| *Li* | PAW_PBE Li 17Jan2003 | 140.000 | *W* | PAW_PBE W 08Apr2002 | 223.057 |
| *Mg* | PAW_PBE Mg 13Apr2007 | 200.000 | *Y* | PAW_PBE Y_sv 25May2007 | 202.626 |
| *Mn* | PAW_PBE Mn 06Sep2000 | 269.865 | *Zn* | PAW_PBE Zn 06Sep2000 | 276.727 |
| *Mo* | PAW_PBE Mo 08Apr2002 | 224.584 | *Zr* | PAW_PBE Zr_sv 04Jan2005 | 229.898 |
| *Na* | PAW_PBE Na 08Apr2002 | 101.968 | | | |

**Supplementary Table 2.** List of nearest neighbor distances of all 55 elements used in this work.

| Element | Nearest Neighbor Distance (Å) | Element | Nearest Neighbor Distance (Å) |
|---|---|---|---|
| *Ag* | 2.895913 | *Nb* | 2.870738 |
| *Al* | 2.855954 | *Ni* | 2.46851 |
| *As* | 2.550183 | *Os* | 2.684334 |
| *Au* | 2.915931 | *P* | 2.197732 |
| *B* | 1.666025 | *Pb* | 3.555145 |
| *Ba* | 4.356367 | *Pd* | 2.774875 |
| *Be* | 2.201364 | *Pt* | 2.794795 |
| *Bi* | 3.099441 | *Rb* | 4.492154 |
| *C* | 1.421378 | *Re* | 2.74767 |
| *Ca* | 3.817938 | *Rh* | 2.694163 |
| *Cd* | 2.978816 | *Ru* | 2.640016 |
| *Co* | 2.458547 | *S* | 2.059424 |
| *Cr* | 2.446799 | *Sb* | 3.083376 |
| *Cs* | 4.631936 | *Sc* | 3.210087 |
| *Cu* | 2.548771 | *Se* | 2.361713 |
| *Fe* | 2.442529 | *Si* | 2.366088 |
| *Ga* | 2.525538 | *Sn* | 2.877918 |
| *Ge* | 2.500974 | *Sr* | 4.236609 |
| *Hf* | 3.12281 | *Ta* | 2.858984 |
| *Hg* | 3.291589 | *Te* | 2.903174 |
| *In* | 3.279844 | *Ti* | 2.627395 |
| *Ir* | 2.731446 | *Tl* | 3.421145 |
| *K* | 4.145087 | *V* | 2.570188 |
| *Li* | 2.967711 | *W* | 2.740305 |
| *Mg* | 3.150178 | *Y* | 3.541122 |
| *Mn* | 2.127518 | *Zn* | 2.611721 |
| *Mo* | 2.721704 | *Zr* | 3.162049 |
| *Na* | 3.383385 | | |

**Supplementary Table 3.** Energy of an isolated atom for 55 different elements calculated using DFT.

| Element | $E_{atom}$ (eV/atom) | Element | $E_{atom}$ (eV/atom) |
|---|---|---|---|
| *Ag* | -0.20678238 | *Nb* | -2.5290874 |
| *Al* | -0.31502421 | *Ni* | -0.32229605 |
| *As* | -1.70347393 | *Os* | -2.9235101 |
| *Au* | -0.18460885 | *P* | -1.89085386 |
| *B* | -0.46684362 | *Pb* | -0.58444951 |
| *Ba* | -0.03489553 | *Pd* | -1.47706062 |
| *Be* | -0.03862034 | *Pt* | -0.4479688 |
| *Bi* | -1.32698449 | *Rb* | -0.14078528 |
| *C* | -1.37041552 | *Re* | -3.42541225 |
| *Ca* | -0.06131973 | *Rh* | -1.55608394 |
| *Cd* | -0.17270692 | *Ru* | -2.48498773 |
| *Co* | -1.89843675 | *S* | -1.07740284 |
| *Cr* | -5.4337722 | *Sb* | -1.41325936 |
| *Cs* | -1.80010576 | *Sc* | -1.98280265 |
| *Cu* | -0.2482986 | *Se* | -0.88066384 |
| *Fe* | -3.46408472 | *Si* | -0.87300431 |
| *Ga* | -0.2782155 | *Sn* | -0.64398885 |
| *Ge* | -0.77222759 | *Sr* | -0.06657764 |
| *Hf* | -3.47421688 | *Ta* | -3.66584012 |
| *Hg* | -0.1240558 | *Te* | -0.73243934 |
| *In* | -0.22810749 | *Ti* | -2.25079318 |
| *Ir* | -1.63543721 | *Tl* | -0.19704188 |
| *K* | -0.15644111 | *V* | -3.49576063 |
| *Li* | -0.29370456 | *W* | -4.10747422 |
| *Mg* | -0.00092076 | *Y* | -2.23850182 |
| *Mn* | -5.16311873 | *Zn* | -0.16649868 |
| *Mo* | -3.30513752 | *Zr* | -2.27940671 |
| *Na* | -0.2205613 | | |

## Supplementary Note 3. DFT calculation strategies for treating magnetic clusters

The final magnetic state of a cluster may be in a local minimum instead of the global minimum. To reduce the chance of this happening, for each of the magnetic elements (*Fe*, *Mn*, *Co*, *Ni*, *Ru*, *Rh*, *V*, *Cu*, and *Cr*) we evaluated the effect of different initial magnetic moments (+1 $\mu_B$/atom, +2$\mu_B$/atom, +3$\mu_B$/atom, and +5 $\mu_B$/atom) on total energies and final magnetic states, as set using the MAGMOM parameter in VASP. We evaluated the final magnetic states and energies of 2228 clusters with 3-55 atoms selected from an early version of the QCD. We found that for *Co*, *Cu* and *Ni*, the final magnetic state is almost always independent of MAGMOM, whereas for the others (*Fe*, *Mn*, *Ru*, *Rh*, *V*, and *Cr* ) it is common for the cluster to get trapped in a distinct local minimum, making it important to initialize calculations with multiple MAGMOM values. Often clusters in the QCD with an odd (even) number of electrons would have a net magnetic moment of 1 $\mu_B$ (0 $\mu_B$) per cluster. This behavior was largely independent of MAGMOM but results from the single unpaired electron. We list the elements which retain non-zero magnetic moments after relaxation and the effects of multiple magnetic initializations on magnetic moments after relaxation in Supplementary Table 4.

**Supplementary Table 4.** Finite magnetic moments after relaxation with default and multiple magnetic initializations per atom using the MAGMOM flag in VASP. This analysis was done by considering clusters of all sizes between 3-55 atoms for each magnetic element.

| Element | Mean absolute magnetic moments in ($\mu_B$/atom) with default magnetic initialization in VASP | STD DEV | Mean absolute magnetic moments in ($\mu_B$/atom) with multiple magnetic initializations in VASP | STD DEV |
|---|---|---|---|---|
| *Fe* | 3.05 | 0.17 | 2.77 | 0.36 |
| *Mn* | 2.76 | 0.63 | 2.33 | 1.04 |
| *Co* | 1.92 | 0.18 | 1.92 | 0.104 |
| *Ni* | 0.8 | 0.12 | 0.77 | 0.081 |
| *Ru* | 0.7 | 0.63 | 0.69 | 0.388 |
| *Rh* | 0.69 | 0.32 | 0.654 | 0.341 |
| *V* | 0.39 | 0.92 | 0.404 | 0.455 |
| *Cu* | 0.03 | 0.06 | 0.036 | 0.056 |
| *Cr* | 0.45 | 0.42 | 0.954 | 0.685 |

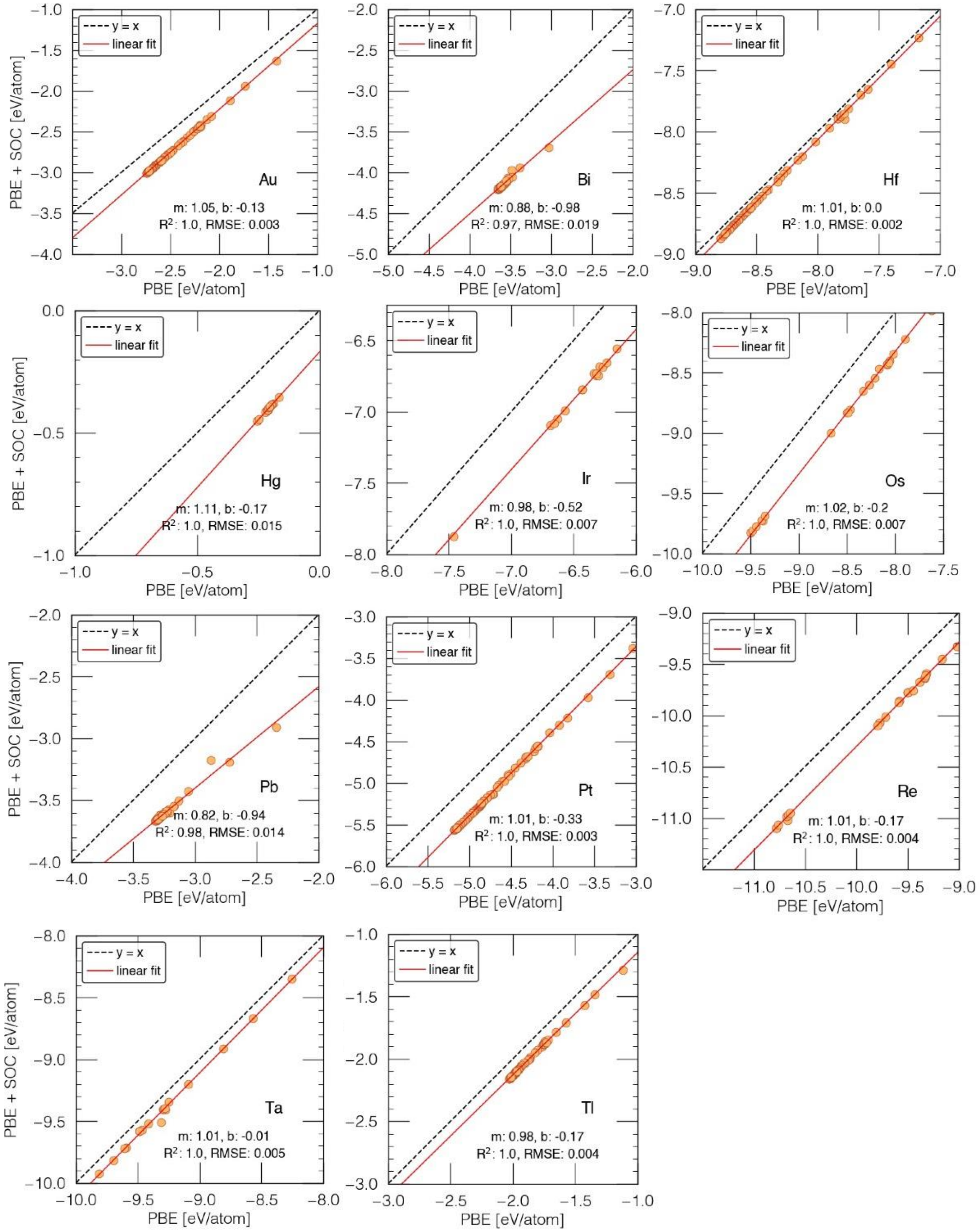


**Supplementary Figure 3.** PBE + SOC energy conversion from PBE energies using least-square linear regression fit $E_{PBE+SOC} = m * E_{PBE} + b$. The linear parameters (slope $m$ and intercept $b$) along with their RMSE error are provided for each element in the legend of each subplot.

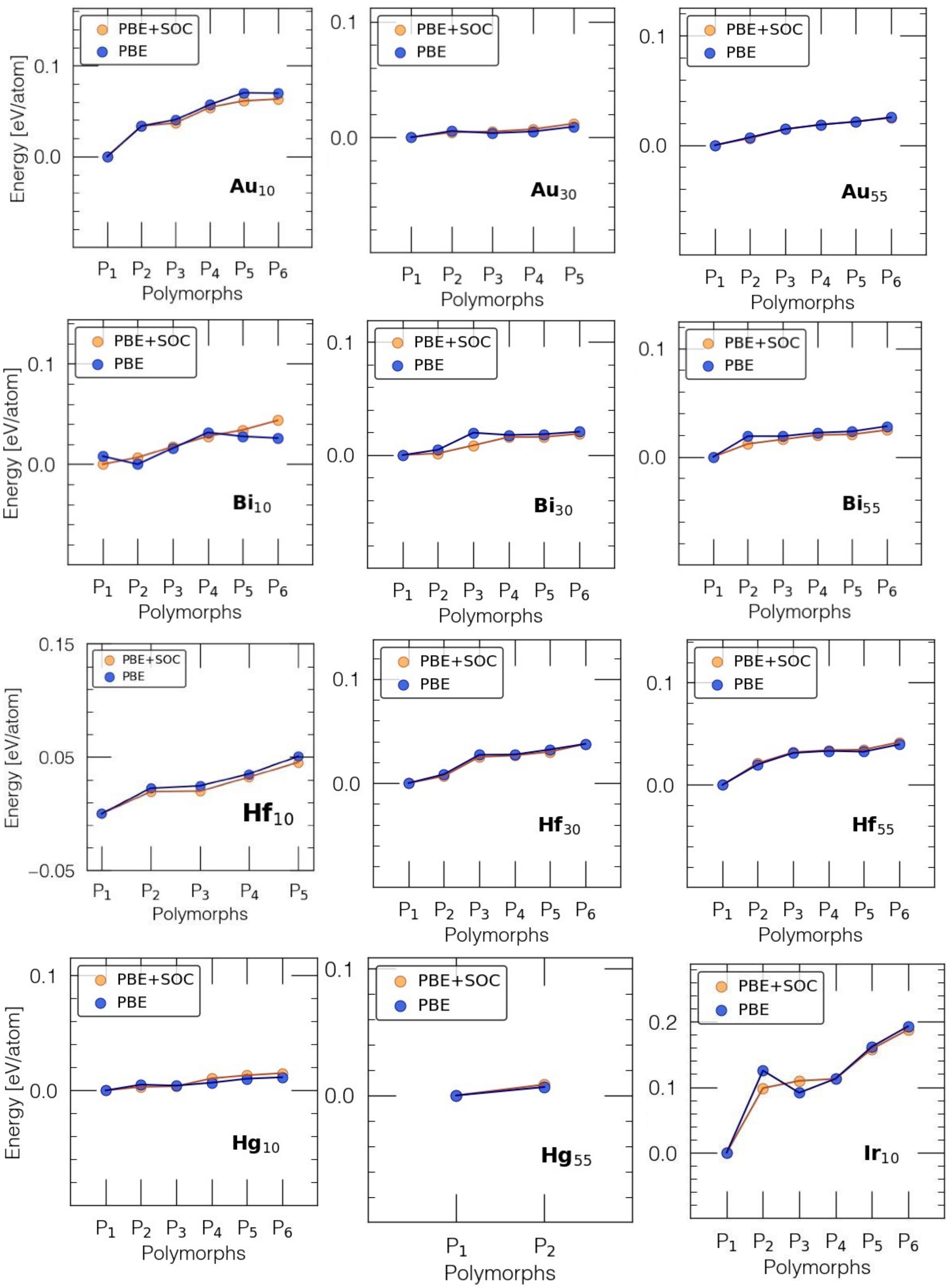


**Supplementary Figure 4.** Effect of PBE + SOC on relative energy ordering among isomers of *Au*, *Bi*, *Hf*, *Hg*, and *Ir* clusters. Relative energies with respect to the lowest energy isomers are plotted for each polymorph, and the polymorphs are sorted based on the PBE+SOC energies. One $Hf_{10}$ structure was not included in this plot because PBE and PBE+SOC relaxed to dissimilar structures.

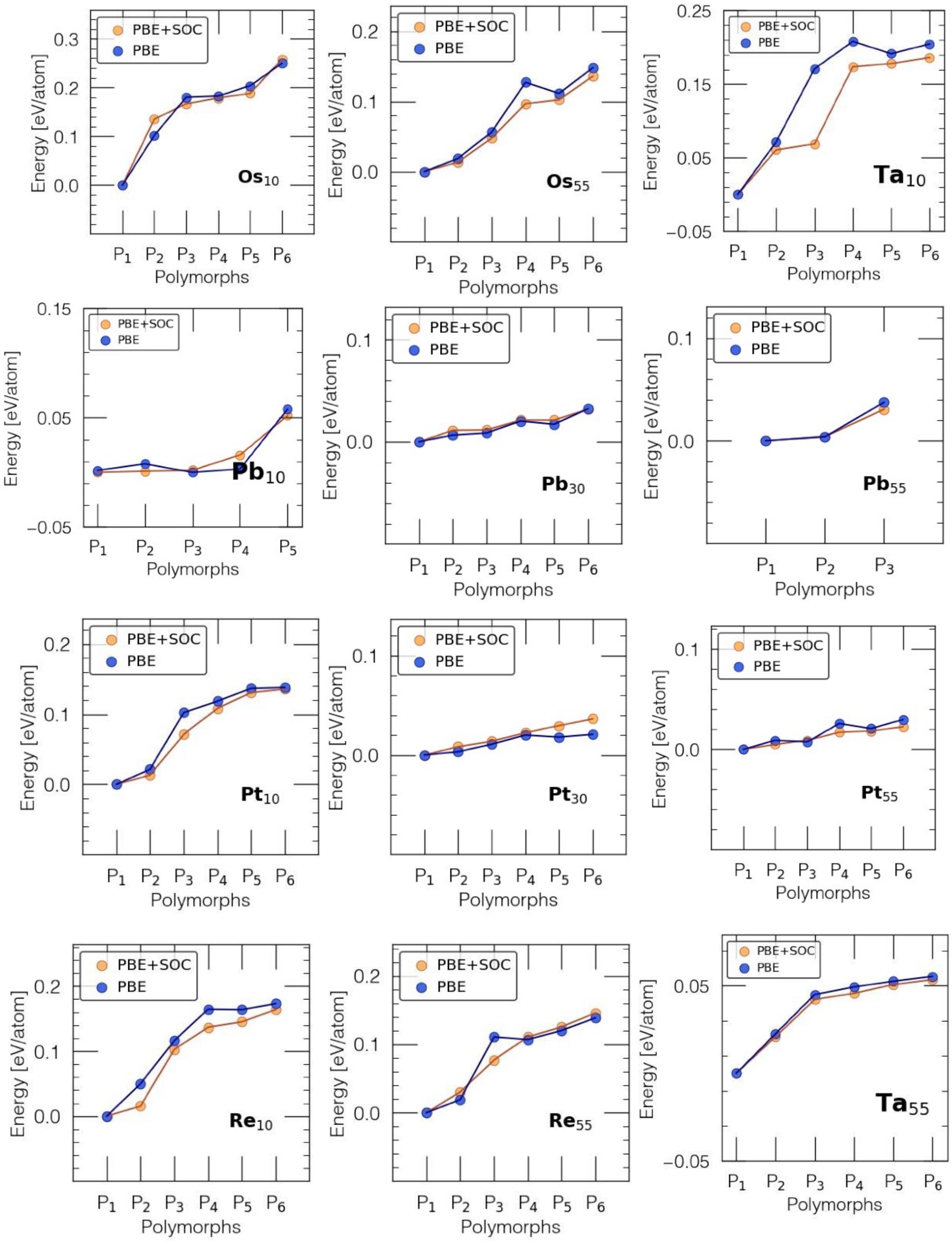


**Supplementary Figure 5.** Effect of PBE +SOC on relative energy ordering among the isomers of *Os*, *Ta*, *Pb*, *Pt*, and *Re* clusters. Element names and their sizes are stamped in each subplot. Relative energies with respect to lowest energy isomer are plotted against each polymorph which is sorted with respect to PBE+SOC energies. One $Pb_{10}$ structure was not included in this plot because PBE and PBE+SOC relaxed to dissimilar structures.

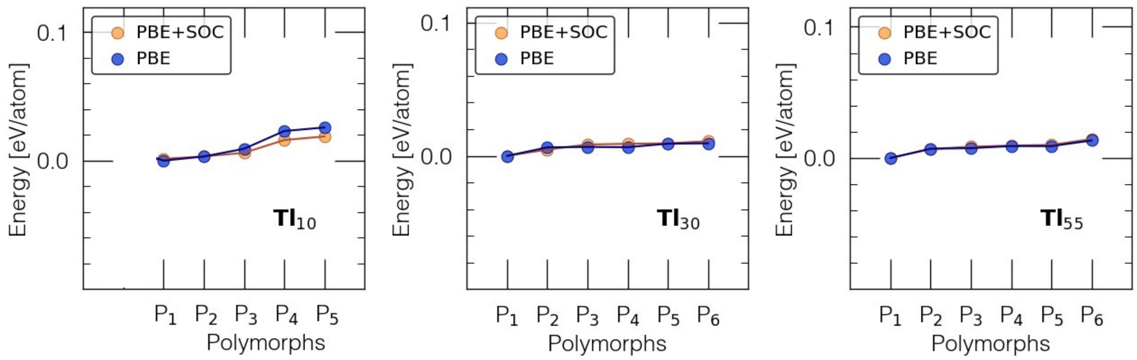


**Supplementary Figure 6.** Effect of PBE +SOC on relative energy ordering among the isomers of *Tl* clusters. Element names and their sizes are stamped in each subplot. Relative energies with respect to lowest energy isomer are plotted against each polymorph which is sorted with respect to PBE+SOC energies. One $Tl_{10}$ structure was not included in this plot because PBE and PBE-SOC relaxed to dissimilar structures.

## Supplementary Note 4. Box size analysis

To investigate whether the box sizes used in the QCD are sufficiently large, we performed benchmarks on 1335 clusters from 49 different elements with 3-55 atoms selected from the QCD. In all cases, energies were converged within 2 meV/atom, with a root mean square error of just 0.118 meV/atom.

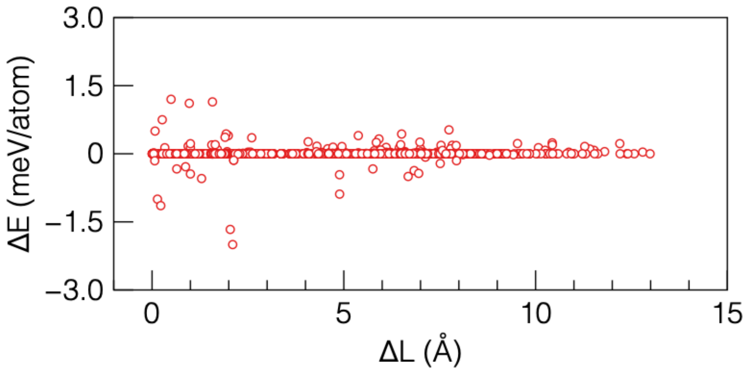


**Supplementary Figure 7.** Effect of changes in the computational box length from the currently used box length on predicting total energies.

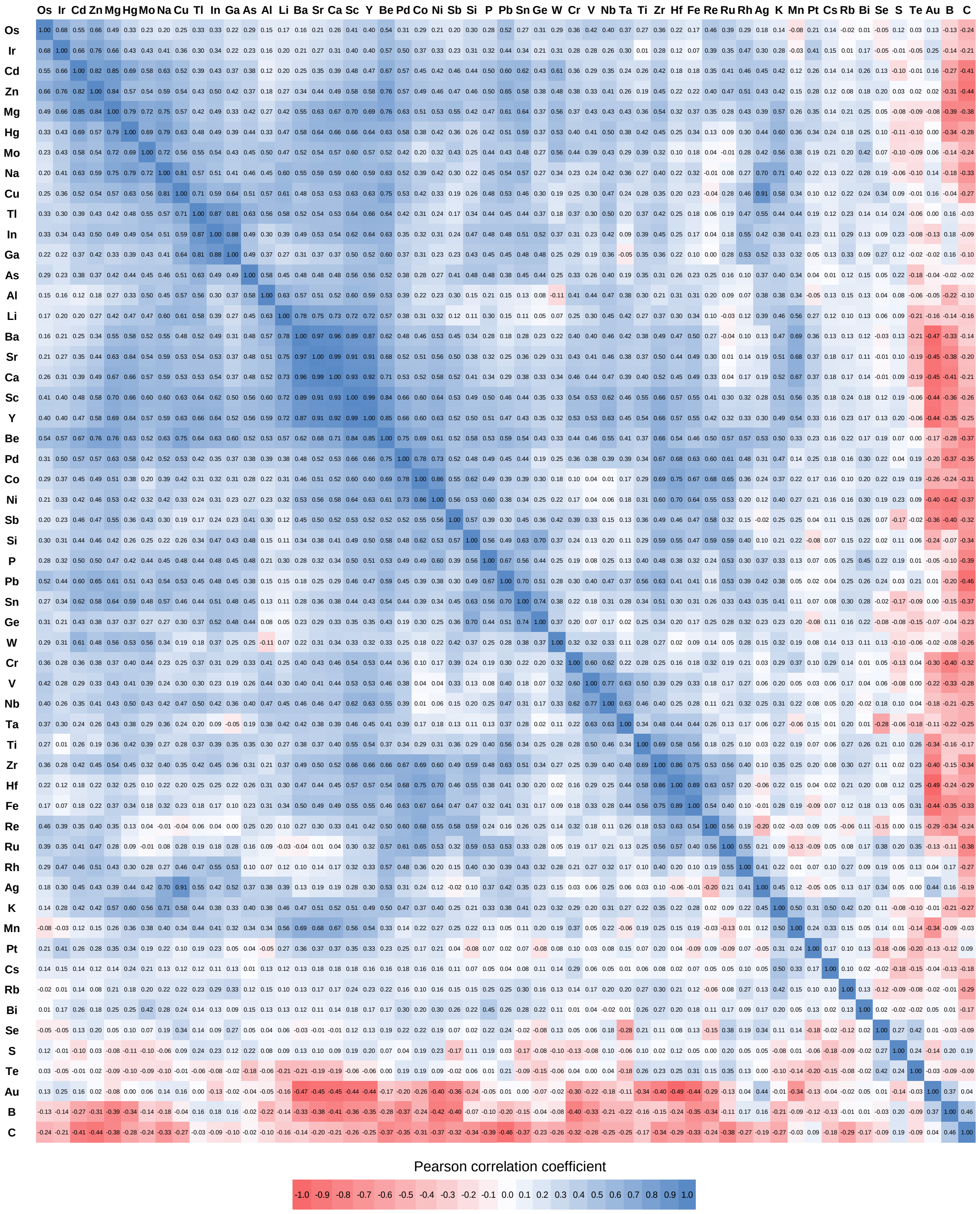


**Supplementary Figure 8.** The Pearson correlation coefficients for all 55 elements calculated from the energies of relaxed structures, sorted by the mean absolute difference in correlation coefficients calculated from unrelaxed (top) and relaxed (bottom) structures. The data used to generate this figure and Figure 2 of the main text are available as a csv file at the header of the QCD website homepage.

## Supplementary Note 5. Template Structures Set 1

The following structures are the low-energy clusters used in the first step of the two-step process for determining the chemical correlations among elements, as laid out in the main text section titled "Clusters built from low-energy structures of chemically similar elements". They include clusters of 5, 10, 15 and 20 atoms for the elements of Al, Be, Li, Mg, Na, Si, Ta, and Ti. All structures are provided in the POSCAR format used by the Vienna Ab initio Simulation Package (VASP).

```
Template Cluster: Al-10-1
   1.00000000000000
    15.4462123787814498    0.0000000000000000    0.0000000000000000
     0.0000000000000000   15.4462123787814498    0.0000000000000000
     0.0000000000000000    0.0000000000000000   15.4462123787814498
   Al
    10
Direct
  0.5820601806025661  0.5858778994050856  0.4546761976671014
  0.5300768830098469  0.5619497549366886  0.6380095505211819
  0.6247196578231532  0.4245020923491276  0.5459754529073390
  0.4183046319566657  0.5576393624957978  0.5185634173626266
  0.4668024469635267  0.3998614323973500  0.6186312895344629
  0.6864769185661300  0.5718595726982545  0.5876514619561677
  0.3459086562043368  0.3892157991586050  0.5076003419432868
  0.5082005538366001  0.4244456573822307  0.4313487181667364
  0.4780769271880695  0.5971455075470382  0.3247155025409946
  0.3593731438491200  0.4875029216298140  0.3728280674001182
```

```
Template Cluster: Al-15-1
   1.00000000000000
    15.3102150587935508    0.0000000000000000    0.0000000000000000
     0.0000000000000000   15.3102150587935508    0.0000000000000000
     0.0000000000000000    0.0000000000000000   15.3102150587935508
   Al
    15
Direct
  0.6373227610237668  0.5497382138085208  0.4043546782970208
  0.4862712614508993  0.4711017933127472  0.3274934865154067
  0.4693611085362258  0.7561106310110076  0.5031322614063500
  0.4813354040465358  0.6359739704728282  0.3848191970353242
  0.6061935388964913  0.3790184449096977  0.4198991621363928
  0.5933293726029565  0.6385424722653692  0.5534775407728589
  0.6549786668599165  0.4703464205431256  0.5713798801820548
  0.4992935604054952  0.5001257969457671  0.4997466495911192
  0.5109346453703867  0.5315810053223345  0.6711283972718942
  0.5430964589849235  0.2467541353831844  0.5030743958482740
  0.3419882779089082  0.5213694698108678  0.4264940523820751
  0.3920279111398168  0.6231986623707191  0.5731631846657277
  0.5118829249272959  0.3655692910365597  0.6186937503652271
  0.4122383294218885  0.3574994727626286  0.4488305386920710
  0.3597457784244861  0.4530702200446566  0.5943128248381954
```

```
Template Cluster: Al-20-1
   1.00000000000000
    17.0062937660931404    0.0000000000000000    0.0000000000000000
     0.0000000000000000   17.0062937660931404    0.0000000000000000
     0.0000000000000000    0.0000000000000000   17.0062937660931404
   Al
    20
Direct
  0.6416950053154078  0.4018320978632332  0.6259603232161369
  0.7105881746869647  0.4911355849952922  0.4982746759552596
  0.5280449716776099  0.3039301792642833  0.5468234920171995
  0.5460659599455288  0.5179715489011852  0.7044231559954314
  0.6596899331791793  0.5724686055073926  0.6175981530400023
  0.5000471771337002  0.3950400492464550  0.3994426565675820
  0.3934525993509982  0.4053084964140127  0.5283609321884293
  0.6457374659542009  0.3570486365813525  0.4627773133577371
  0.5478245029318102  0.4732677000044136  0.5404117557049603
  0.4784969091505020  0.3851183794448678  0.6687719033068099
  0.5956856360122820  0.5369921392776345  0.3940158177566209
  0.4180470745576619  0.5515379982114506  0.6097165873455539
  0.5438629106897194  0.6396860156520651  0.5271708299931552
  0.4470906731479258  0.5366134433348558  0.4529134368867555
  0.3455828596517876  0.4379724087584153  0.3764648069833783
  0.4694451080764566  0.5171443427016184  0.2949062060301529
  0.2951319248187453  0.5327761448171265  0.5051834136524541
  0.4978698560823974  0.6668801106974244  0.3736684217895109
  0.3866253825232056  0.6754055504775187  0.5068208631733169
  0.3490158751138965  0.6018705678493904  0.3662952550395381
```

```
Template Cluster: Al-5-1
   1.00000000000000
    13.6898347767111606    0.0000000000000000    0.0000000000000000
     0.0000000000000000   13.6898347767111606    0.0000000000000000
     0.0000000000000000    0.0000000000000000   13.6898347767111606
   Al
     5
Direct
  0.5593038719523212  0.3455827072384002  0.3987720807633727
  0.5268997453497222  0.6183270633050716  0.5646495650039777
  0.3387723995375835  0.5863014875858823  0.5641088531388047
  0.4386744030325506  0.4514188444890291  0.4887536489731359
  0.6363495801278218  0.4983698973816175  0.4837158521206879
```

```
Template Cluster: Be-10-1
   1.00000000000000
    13.6369959856906497    0.0000000000000000    0.0000000000000000
     0.0000000000000000   13.6369959856906497    0.0000000000000000
     0.0000000000000000    0.0000000000000000   13.6369959856906497
   Be
    10
Direct
  0.5127429012482247  0.4803089855498241  0.6271572607657686
  0.5147249392958867  0.3719135491097197  0.5077513966577658
  0.6234213890905715  0.4726666713893775  0.5269598227423917
  0.5415124585191086  0.6087103497807320  0.5561207665056149
  0.6134009824555634  0.5843017838145558  0.4255124433380172
  0.3865109602470370  0.4158498918361423  0.5745078824407789
  0.4012476994069457  0.4555897472769899  0.4294913258694583
  0.4627938498644039  0.5912988357627855  0.4163214762806422
  0.5446418110702839  0.4552181102610847  0.3872875047549201
  0.3990030088019813  0.5641420752187810  0.5488901206446493
```

Template Cluster: Be-15-1

```
   1.00000000000000
    15.1560308903273295    0.0000000000000000    0.0000000000000000
     0.0000000000000000   15.1560308903273295    0.0000000000000000
     0.0000000000000000    0.0000000000000000   15.1560308903273295
   Be
    15
Direct
  0.4455754620206330  0.6615297998289625  0.5999435377169998
  0.5181139837116222  0.5558583645325228  0.6389769128308693
  0.5520478144534078  0.6235993197153838  0.5205557587669805
  0.6173400526205229  0.5006720081892382  0.5639733308066258
  0.3938224789713294  0.5352592151403861  0.5775905200009455
  0.4197549018165968  0.6251780714991941  0.4734902748313723
  0.4855845470328718  0.4265986452571889  0.6105877396692749
  0.4998660580332039  0.4998287793021379  0.5000820536006613
  0.6059923527301234  0.4654897193593346  0.4226498797157691
  0.5800057713488874  0.3750218938929709  0.5268597828375876
  0.5132779625315679  0.5732156829463406  0.3890811807290906
  0.5556586225602576  0.3387940087957002  0.4000124724020757
  0.3827246356572405  0.4994642443262515  0.4363726593622091
  0.4484267667885210  0.3757504831647804  0.4788660407013062
  0.4818085897232072  0.4437397640496221  0.3609578560282313
```

```
Template Cluster: Be-20-1
   1.00000000000000
    15.2188144398366596    0.0000000000000000    0.0000000000000000
     0.0000000000000000   15.2188144398366596    0.0000000000000000
     0.0000000000000000    0.0000000000000000   15.2188144398366596
   Be
    20
Direct
  0.6032317676444209  0.3574094723851997  0.5162106975002251
  0.6625079174401247  0.4812805145292768  0.5376433849883714
  0.5604462417921283  0.4377755275697380  0.6183448151709384
  0.4670768368926912  0.3596379246899227  0.5289454029381495
  0.4260797823615507  0.4248704149065118  0.6420326510572334
  0.6353283345993280  0.4366612303937289  0.4136446892924552
  0.5219284505651373  0.3648836626920878  0.4036274864556846
  0.5915484523089656  0.5847328055774118  0.5833297545991200
  0.4892158894070667  0.5472018693406110  0.6607218267082453
  0.6110804506829385  0.5712384140224245  0.4422341196853600
  0.5004179966485220  0.4967313822605245  0.5021210594487966
  0.5230638840788304  0.4982202063384338  0.3578741673500161
  0.4060546070675100  0.4312318694350343  0.4046319265067519
  0.3498151430376329  0.4305167043171966  0.5259066458928509
  0.5703372129140226  0.6878999492158393  0.4982356168594665
  0.3676398338686240  0.5386425183977995  0.6039027042020365
  0.4596533464225459  0.6288389100378923  0.5498781693222083
  0.4839939013575076  0.6204567925294748  0.4127609757303665
  0.3719480357704095  0.5559133856444172  0.4653013627684675
  0.3986319151400505  0.5458564457164955  0.3326525435232702
```

Template Cluster: Be-5-1
1.00000000000000
12.3395075399331606 0.0000000000000000 0.0000000000000000
0.0000000000000000 12.3395075399331606 0.0000000000000000
0.0000000000000000 0.0000000000000000 12.3395075399331606
Be
5
Direct
0.5510167254798145 0.3749970982751942 0.5280081509879564
0.5537437792682306 0.5367885402511469 0.5679363723342506
0.5306227806433478 0.4923172478780282 0.4113145919631347
0.4134461820612199 0.4703881875249631 0.5217903379348274
0.4511705325473803 0.6255089260706675 0.4709505467798236

Template Cluster: Li-10-1

```
   1.00000000000000
    15.4409571314248897    0.0000000000000000    0.0000000000000000
     0.0000000000000000   15.4409571314248897    0.0000000000000000
     0.0000000000000000    0.0000000000000000   15.4409571314248897
   Li
    10
Direct
  0.6600195734881523  0.5849402383177528  0.4305287163893801
  0.5584423305788933  0.5556426613223169  0.5820842894699065
  0.5601898756358833  0.3602399660592025  0.6213666333132556
  0.5557895633441617  0.7401036810802761  0.5157683776606461
  0.5563011313222432  0.4162362617909926  0.4448168370855672
  0.4446423059250548  0.2598289224708794  0.4831162216473653
  0.3906531347286264  0.6362637781532261  0.5861897826653516
  0.4736566209180442  0.5861749136189681  0.4279616917220847
  0.4110453543496159  0.4423809800578375  0.5452723798285601
  0.3892601097093246  0.4181885971285421  0.3628950702178891
```

Template Cluster: Li-15-1
1.00000000000000
16.0400094085654992 0.0000000000000000 0.0000000000000000
0.0000000000000000 16.0400094085654992 0.0000000000000000
0.0000000000000000 0.0000000000000000 16.0400094085654992
Li
15
Direct
0.6558537562606424 0.3694509372767606 0.3849267059911016
0.4999621860783756 0.2694561449657677 0.4646399496467357
0.5124468473104907 0.4338477119184546 0.4260091200647840
0.5882062059280756 0.4051827431828977 0.5733134821290362
0.5456305748747003 0.4918545530326282 0.2700918360240049
0.3895973247859782 0.3799281196321971 0.3347548977296323
0.6188578096696985 0.5635920863403470 0.4367012928299634
0.3871109244181547 0.4096519892311389 0.5355950142777676
0.4214288393872972 0.5723371241207282 0.3961092086433680
0.4896383401444568 0.5544220599298076 0.5610726744777057
0.6277048065268259 0.5944647591732408 0.6383084450091919
0.4602081868223604 0.4788100832890235 0.7053791774388234
0.3332495974771890 0.5987138485261114 0.5759199019969372
0.5058568331859778 0.7105857424517684 0.5085112634156596
0.4642477671297840 0.6677020969291423 0.6886670303252889

Template Cluster: Li-20-1
1.00000000000000
16.4792629169544398 0.0000000000000000 0.0000000000000000
0.0000000000000000 16.4792629169544398 0.0000000000000000
0.0000000000000000 0.0000000000000000 16.4792629169544398
Li
20
Direct
0.6765650001439119 0.6287812244489110 0.4578535621662987
0.5354260165998967 0.5476486213607944 0.3577793569478361
0.5113946168960044 0.7236380876417765 0.4440280025180220
0.6897102557990129 0.4454770389529123 0.4018600450424503
0.6030305516750202 0.6918362007933477 0.6221470818932191
0.3929464549222018 0.5432848454806462 0.2512173870112156
0.6680323422607952 0.4875331635469203 0.5840381558953459
0.5484062945499322 0.4221293389650718 0.4780401338193852
0.5364348565509611 0.5838201233194990 0.5252228334151984
0.5371982871246110 0.3622234225235297 0.3234714762757174
0.3911629971109777 0.4369222424573637 0.3912173760120545
0.3788162392732114 0.5984012327189886 0.4282272610677291
0.6354045538262213 0.2903629940269120 0.4977168186959954
0.4199077770180342 0.4771270192192237 0.5601755623920974
0.5344607404965417 0.3661084323421974 0.6424575323959979
0.3908647477151827 0.6375967532105286 0.6067707069546681
0.5176571574319314 0.5369390749176832 0.6923075936937547
0.2630493794323454 0.4803231436595617 0.5200899757610198
0.4138882919892394 0.3126982400199517 0.5195595695760676
0.3556434391839752 0.4271488003941804 0.6958195684659284

```
Template Cluster: Li-5-1
   1.00000000000000
    15.8002140862901701    0.0000000000000000    0.0000000000000000
     0.0000000000000000   15.8002140862901701    0.0000000000000000
     0.0000000000000000    0.0000000000000000   15.8002140862901701
   Li
     5
Direct
  0.5829509350755859  0.4838035379454061  0.6356858570268806
  0.5621140307824140  0.5665101389986219  0.4702564414260484
  0.4156056474062327  0.5362797515493614  0.5544367498275891
  0.5209082404713722  0.3991134950367526  0.4754465979422424
  0.4184211462643954  0.5142930764698509  0.3641743537772326
```

Template Cluster: Mg-10-1
1.00000000000000
13.9279393065084296 0.0000000000000000 0.0000000000000000
0.0000000000000000 13.9279393065084296 0.0000000000000000
0.0000000000000000 0.0000000000000000 13.9279393065084296
Mg
10
Direct
0.5274702891744628 0.4243725904976898 0.6691748555686559
0.5085579615452142 0.6408854087535923 0.6669093902353243
0.6111995989807646 0.3502877071204454 0.4757265460736600
0.3107206105018636 0.6074025868047953 0.3767856914182747
0.4064447570695745 0.3052923631724888 0.5331964379992564
0.3599837838643435 0.5166925554028517 0.5652295594858923
0.5086623692605312 0.6464479939891250 0.4487566808722970
0.6490745806154853 0.5170435189584290 0.3418108551876290
0.6722620230429709 0.5503714164764130 0.5551064866676493
0.4456240259447964 0.4412038588241762 0.3673034964913536

```
Template Cluster: Mg-15-1
   1.00000000000000
    17.7525718978898794    0.0000000000000000    0.0000000000000000
     0.0000000000000000   17.7525718978898794    0.0000000000000000
     0.0000000000000000    0.0000000000000000   17.7525718978898794
   Mg
    15
Direct
  0.7094640650344919  0.4846321130017932  0.4991141266328399
  0.6443707551328710  0.6243076277160032  0.4121545102593260
  0.6304316733024420  0.6101816225873586  0.5798639275104095
  0.6319895999682225  0.4629176332066765  0.3414634726947217
  0.4808575860902066  0.6087194278193280  0.4943062747302300
  0.5069221841423162  0.5754513616434567  0.3271167689032464
  0.6112345080793945  0.3510200686427885  0.4664107465998227
  0.5512537658848374  0.4572420858930383  0.5881881174914184
  0.4648528925608562  0.5853043171459498  0.6642950126990539
  0.4680453302497627  0.4343035559955689  0.4181725445058253
  0.3410863948405886  0.5511454277569788  0.4112622470954007
  0.4452702487391462  0.3264168059617605  0.5506896261497525
  0.3204019045660907  0.5765690610444727  0.5770277032794066
  0.3852906129089142  0.4365980942904066  0.6638974785994028
  0.3085284784998670  0.4151907972943980  0.5060374428491515
```

```
Template Cluster: Mg-20-1
   1.00000000000000
    18.6776942262281906    0.0000000000000000    0.0000000000000000
     0.0000000000000000   18.6776942262281906    0.0000000000000000
     0.0000000000000000    0.0000000000000000   18.6776942262281906
   Mg
    20
Direct
  0.5041880133814856  0.5111862483160313  0.2550452542986978
  0.6146877647616675  0.5103847612550363  0.3733630241056265
  0.4929285192982629  0.3949951347186825  0.3675673712774492
  0.6163866156843991  0.3836773521324836  0.4778249574354327
  0.7005695659642248  0.5207520915470950  0.5129037593163149
  0.4758189380266438  0.2943129164275938  0.4911724848532260
  0.3696097385934454  0.4992464121602364  0.3391214938996224
  0.6265403792786608  0.6505012323152829  0.4546737716713893
  0.4843273936047268  0.6138275171332609  0.3799275884155823
  0.5805641435815442  0.2965755108656210  0.6109461673311060
  0.6051836733147987  0.4555994667146856  0.6272970224154911
  0.3533921924669053  0.5806933116274120  0.4761021280152607
  0.6199273793832640  0.6180647318160849  0.6116327207342288
  0.3647983956387716  0.4122317413210523  0.4761236851947843
  0.4848850826426578  0.6667780185474130  0.5354349219008412
  0.4498134743126252  0.3922694921758014  0.6201278963659418
  0.3256020833645607  0.4931331496479462  0.6116802713219988
  0.5054867622724032  0.5036276875998822  0.5008193077759600
  0.4755352737615446  0.5520408549869954  0.6555843961996066
  0.3497546106674074  0.6501023686914180  0.6226517774714355
```

```
Template Cluster: Mg-5-1
   1.00000000000000
    13.0249268186004894    0.0000000000000000    0.0000000000000000
     0.0000000000000000   13.0249268186004894    0.0000000000000000
     0.0000000000000000    0.0000000000000000   13.0249268186004894
   Mg
     5
Direct
  0.5356409998062875  0.5038427985495322  0.3726298057220881
  0.3712779558942489  0.5059636188134600  0.5323451010562397
  0.5124804872646325  0.7160085650522588  0.5102565855128876
  0.5916792778143888  0.4902486531001509  0.5948079949342248
  0.4889212792204491  0.2839363644845981  0.4899605127745747
```

Template Cluster: Na-10-1
1.00000000000000
22.6269584191506112 0.0000000000000000 0.0000000000000000
0.0000000000000000 22.6269584191506112 0.0000000000000000
0.0000000000000000 0.0000000000000000 22.6269584191506112
Na
10
Direct

| | | |
|---|---|---|
| 0.5869258320540955 | 0.4544113642597072 | 0.3758763784905891 |
| 0.5517230418022505 | 0.5740611736827154 | 0.4664775002301365 |
| 0.5623066397581026 | 0.4338067083136865 | 0.5307724332377115 |
| 0.4908964166883482 | 0.5599765399697171 | 0.3083713795140017 |
| 0.5859202970560418 | 0.5566194860132850 | 0.6203264397208730 |
| 0.4425768573584615 | 0.5351179701799892 | 0.5686403161930392 |
| 0.4445944615730962 | 0.4575075975667956 | 0.4338940549593610 |
| 0.5080783658990606 | 0.4395457540832449 | 0.6916323307204231 |
| 0.4045950942585204 | 0.6062416840776416 | 0.4326462880767854 |
| 0.4223829935520367 | 0.3827117218532172 | 0.5713628788570786 |

Template Cluster: Na-15-1
1.00000000000000
17.1225094045466584 0.0000000000000000 0.0000000000000000
0.0000000000000000 17.1225094045466584 0.0000000000000000
0.0000000000000000 0.0000000000000000 17.1225094045466584
Na
15
Direct
0.3931820401640386 0.3864551767770027 0.6099408766564711
0.4169300838200083 0.4564659743515918 0.4184142345551283
0.5661114886663254 0.4932244494267701 0.5725118877301000
0.3475474609931123 0.6492841715598276 0.3633203001094538
0.4880523142562329 0.5376515042058859 0.2436947531987713
0.5670956470358587 0.3265718027179111 0.7013556239162211
0.5361615252872172 0.6938932738210715 0.6408571851342083
0.4848077848462253 0.5190518554205908 0.7558529947226571
0.7128746403706614 0.3521814100668385 0.5535960172942272
0.4213636738748353 0.7916107690564707 0.5012429720185941
0.6214293401425617 0.4473506211284681 0.3846607668648595
0.3770394495573410 0.5957655321307298 0.5655552806172884
0.5425075410929362 0.6385155272411124 0.4274926753298181
0.5264112626241118 0.2884592698604668 0.4858876188376631
0.4984857472685474 0.3235186622352684 0.2756168130145305

Template Cluster: Na-20-1
1.00000000000000
17.4327374952984684 0.0000000000000000 0.0000000000000000
0.0000000000000000 17.4327374952984684 0.0000000000000000
0.0000000000000000 0.0000000000000000 17.4327374952984684
Na
20
Direct

| | | |
|---|---|---|
| 0.5300415444102774 | 0.7688725767827760 | 0.4850355674177873 |
| 0.6607739692636212 | 0.6200815290285061 | 0.3995572847947069 |
| 0.6621785356228810 | 0.4565157471402402 | 0.5227440851489187 |
| 0.7373645005959966 | 0.4389588010925441 | 0.3312976241793041 |
| 0.6336927616130283 | 0.6356448367859822 | 0.6183200034340181 |
| 0.6895830791419307 | 0.4668608914475034 | 0.7268073770198747 |
| 0.4918294787949792 | 0.5738940931993221 | 0.4934575105703758 |
| 0.6053712146413482 | 0.2982925194308840 | 0.4127810601319826 |
| 0.5403971100818392 | 0.4677717597714139 | 0.3139988455004813 |
| 0.4735575202340955 | 0.6608636778551131 | 0.3118091126995810 |
| 0.5783066261435205 | 0.3140454835159677 | 0.6369781765963880 |
| 0.4054877614557361 | 0.3161638432536821 | 0.3242972267158343 |
| 0.4551468935722080 | 0.3865682119697644 | 0.5025763479256646 |
| 0.4315479831497153 | 0.6847477088309860 | 0.6523255287189175 |
| 0.3326205404095607 | 0.6958932644530019 | 0.4657363008524387 |
| 0.3398713126339329 | 0.5075843839454618 | 0.3733385723376559 |
| 0.4947723425416600 | 0.4948162687463873 | 0.6923807751720902 |
| 0.3141542498213150 | 0.5219031857465560 | 0.5828660974252402 |
| 0.3642077163501123 | 0.3400944387200241 | 0.6721703288257463 |
| 0.2590948595222496 | 0.3504267782839037 | 0.4815221745329938 |

Template Cluster: Na-5-1

```
   1.00000000000000
    18.5858386328242311    0.0000000000000000    0.0000000000000000
     0.0000000000000000   18.5858386328242311    0.0000000000000000
     0.0000000000000000    0.0000000000000000   18.5858386328242311
   Na
     5
Direct
  0.4066093296652613  0.4323420376879591  0.5699732725001279
  0.4871474568364066  0.4079499044965365  0.4012929401940449
  0.4593144164842857  0.5721019886003105  0.6760400288319133
  0.6097175197228824  0.5311222969106433  0.3426274994193516
  0.5372112772911714  0.5564837723045433  0.5100662590545632
```

Template Cluster: Si-10-1

```
   1.00000000000000
    15.2292551479609699    0.0000000000000000    0.0000000000000000
     0.0000000000000000   15.2292551479609699    0.0000000000000000
     0.0000000000000000    0.0000000000000000   15.2292551479609699
   Si
    10
Direct
  0.6018049260523831  0.4783883350687850  0.5588821563205808
  0.5835429665789702  0.6289431677677211  0.5870453556315571
  0.6208138025067780  0.4645415936287538  0.3994281593181307
  0.5197004831201114  0.3626168596606734  0.4772559264983898
  0.5395421027008100  0.5971779683994818  0.4425471797054497
  0.4460689247162635  0.5590975629326997  0.5885729246811493
  0.4614027005261265  0.4743047444995210  0.3674678985510542
  0.4754073761636912  0.4057343132744849  0.6281317811179944
  0.3782842809922700  0.5906481829001294  0.4458017061113678
  0.3734324366426027  0.4385472718677499  0.5048669120643317
```

```
Template Cluster: Si-15-1
   1.00000000000000
    16.1625887318440995    0.0000000000000000    0.0000000000000000
     0.0000000000000000   16.1625887318440995    0.0000000000000000
     0.0000000000000000    0.0000000000000000   16.1625887318440995
   Si
    15
Direct
  0.6191108154612415  0.5839149579050928  0.3770434023591026
  0.5877752596186423  0.5173342587602804  0.5192586559747158
  0.4729087993746831  0.6324803125899717  0.3661243012395383
  0.6223250866522081  0.4311467085268135  0.3993765758331083
  0.3767469122904137  0.5124407968782518  0.3801214670144171
  0.5550128928541975  0.6632623540991062  0.4931162318545814
  0.5129302967495514  0.4941040864303713  0.3100523510853321
  0.4796056339410806  0.4254563167198095  0.4506798015790650
  0.4384801511423563  0.5669628826087342  0.5076136993469906
  0.6320359268855696  0.4748445361293154  0.6554046590772404
  0.3591775987387673  0.5666729019778983  0.6343999149641255
  0.4928807898223058  0.5173062387309885  0.6662284717902320
  0.4331890967338902  0.3077863333235724  0.5301550699993278
  0.5316025487345623  0.3783180080632406  0.6117883151514165
  0.3862181910004941  0.4279693072565677  0.5986370827308120
```

```
Template Cluster: Si-20-1
   1.00000000000000
    16.8186754177567686    0.0000000000000000    0.0000000000000000
     0.0000000000000000   16.8186754177567686    0.0000000000000000
     0.0000000000000000    0.0000000000000000   16.8186754177567686
   Si
    20
Direct
  0.4855581126288750  0.6940862967520400  0.5092432747237456
  0.5929182415213873  0.6744236694961709  0.6110016797112712
  0.6293720215092137  0.6836908675689732  0.4696273349437242
  0.4607988381416332  0.6101940797038482  0.6279167955882466
  0.5230769937106579  0.6263373774984246  0.3839722852602882
  0.6999348303186316  0.5855579063620510  0.5571063233453056
  0.4635776713797559  0.5357296979359746  0.4929021213685292
  0.5764104389086377  0.5222669937480374  0.6000776754901332
  0.6142581958254542  0.5320595903314805  0.4525675334091958
  0.3523676096613856  0.4959328337608533  0.5911191040918223
  0.3117691761931597  0.5053724177797645  0.4499089867510723
  0.4176669011273443  0.5096654214015043  0.3479878246114368
  0.4583340880333335  0.3964015676739160  0.5625844704976313
  0.5701540901429153  0.3891677133162429  0.6518341212016313
  0.6175279813311680  0.3846699687778524  0.5227988944062609
  0.4972393200919162  0.4051051550445957  0.4154354707896188
  0.6375748539871752  0.4061534408902248  0.3886468568627852
  0.3131102198915781  0.3729056136234384  0.5186457503884974
  0.3515669855111048  0.3804431203703325  0.3775565299168726
  0.4267834300846726  0.2898362679642533  0.4690669666419515
```

```
Template Cluster: Si-5-1
   1.00000000000000
    12.8453868781241098    0.0000000000000000    0.0000000000000000
     0.0000000000000000   12.8453868781241098    0.0000000000000000
     0.0000000000000000    0.0000000000000000   12.8453868781241098
   Si
     5
Direct
  0.5893665816259935  0.5114674605182834  0.5730710527274692
  0.4287629413088788  0.5932128438984035  0.5724742705092564
  0.5801257229309676  0.5409932385212615  0.3954992521926883
  0.4911989324542617  0.3659782772629846  0.5318837821209284
  0.4105458216799052  0.4883481797990532  0.4270716424496579
```

```
Template Cluster: Ta-10-1
   1.00000000000000
    15.1688356132892803    0.0000000000000000    0.0000000000000000
     0.0000000000000000   15.1688356132892803    0.0000000000000000
     0.0000000000000000    0.0000000000000000   15.1688356132892803
   Ta
    10
Direct
  0.5217397507838961  0.4971262060582857  0.3261183609096984
  0.6316563455139018  0.4635779823185177  0.4467976915571917
  0.5437619727474006  0.6229885425661628  0.4328698723704555
  0.4735614961027237  0.3747525225274354  0.4283074439199205
  0.3856303813830137  0.5340725741523137  0.4145332708096641
  0.5534226058761325  0.3884650191501601  0.5791312535722343
  0.3794653484737240  0.4383361588041931  0.5564365816064486
  0.6032649616203084  0.5639854920270727  0.5823148822729295
  0.4292022433572016  0.6137964828069171  0.5596514077558377
  0.4782948941416832  0.5028990195889422  0.6738392352256266
```

```
Template Cluster: Ta-15-1
   1.00000000000000
    15.1711193809718008    0.0000000000000000    0.0000000000000000
     0.0000000000000000   15.1711193809718008    0.0000000000000000
     0.0000000000000000    0.0000000000000000   15.1711193809718008
   Ta
    15
Direct
  0.5566170372598939  0.3340396369647785  0.4710185590419531
  0.5666855951330122  0.3739254198073383  0.6392788054840641
  0.6524952978704045  0.4479338585777648  0.3826034560036061
  0.6648427244112503  0.4830625884325346  0.5501123125057766
  0.3917778711902949  0.3524537791005392  0.4210539943386182
  0.4033096546396352  0.3871359149537615  0.5887481890228660
  0.4891928735856924  0.4616913643684629  0.3316268195375779
  0.5001045739480193  0.4999816465951782  0.5000700942878566
  0.5965905003984325  0.6127517341356797  0.4110652385774639
  0.3351235670720603  0.5168031490559306  0.4499621772954443
  0.4329312066918970  0.6260006527327535  0.3608769280751299
  0.5108416662628579  0.5385353340430863  0.6683568547884474
  0.6084362468641439  0.6476592451113338  0.5785881829037488
  0.3475665401579812  0.5519887277965739  0.6174824516218583
  0.4434846445144317  0.6660369483242905  0.5291559365155963
```

```
Template Cluster: Ta-20-1
   1.00000000000000
    16.5073565353740399    0.0000000000000000    0.0000000000000000
     0.0000000000000000   16.5073565353740399    0.0000000000000000
     0.0000000000000000    0.0000000000000000   16.5073565353740399
   Ta
    20
Direct
  0.6164071554986977  0.4356345885185880  0.6718067776395200
  0.4612260993814520  0.4102478407983455  0.6938975511390497
  0.5424592655999086  0.3081219556341295  0.5762769117608206
  0.5130856117788726  0.5634436172666759  0.6658209880541673
  0.6811871394075889  0.4039639442666515  0.5341461919672138
  0.3714361892811834  0.5256760806292201  0.6058939607048227
  0.6136832426582515  0.6819047354210231  0.4400754609746489
  0.6373520246460562  0.5698309724110309  0.5686431394898114
  0.3747869443023263  0.3533836898439102  0.5778102036797885
  0.5127303535406186  0.4504272679394205  0.5316952245738841
  0.4825569038801428  0.5985745380092387  0.4933419258084427
  0.6282032869807403  0.5163552850468461  0.4157939759310239
  0.5960553091466186  0.3465296884292952  0.4184422584827537
  0.4490435268517258  0.7264977307341800  0.4082456324416675
  0.4391796031449590  0.3204607286676684  0.4407653005243169
  0.3221908506269834  0.6328477155512637  0.4801891934432810
  0.5365127898376302  0.6231629659165617  0.3202321513506594
  0.3557404131993040  0.4707076174491755  0.4540132866674877
  0.4863842292011067  0.4657503663164285  0.3610791440467289
  0.3797790610358469  0.5964786711503496  0.3418307213199392
```

```
Template Cluster: Ta-5-1
   1.00000000000000
    13.4786745792522709    0.0000000000000000    0.0000000000000000
     0.0000000000000000   13.4786745792522709    0.0000000000000000
     0.0000000000000000    0.0000000000000000   13.4786745792522709
   Ta
     5
Direct
  0.5709774620477225  0.5747061117475544  0.5530616432356424
  0.5472849207148509  0.3964719296083772  0.5885070512104728
  0.4652210849986085  0.6042935430851183  0.4068029705516196
  0.5225235020438197  0.4303153461499842  0.4102829539408783
  0.3939930301949915  0.4942130694089444  0.5413453810613876
```

```
Template Cluster: Ti-10-1
   1.00000000000000
    15.2583201403746997    0.0000000000000000    0.0000000000000000
     0.0000000000000000   15.2583201403746997    0.0000000000000000
     0.0000000000000000    0.0000000000000000   15.2583201403746997
   Ti
    10
Direct
  0.5244498809641637  0.5069827198924781  0.6917220235760955
  0.3756659883234286  0.5400381347720570  0.5961908287414319
  0.4115744688181366  0.6103746429988326  0.4201619696045363
  0.5205292727169083  0.5935091898679670  0.5537942999076271
  0.4856534686145561  0.4013541953101586  0.5830854937024823
  0.4136343794323987  0.4530077458842894  0.4516972362688221
  0.6397796718141893  0.4701536162157639  0.5582950995599364
  0.5793405322367020  0.5528736847508832  0.4118842595949139
  0.4813194658393796  0.4885087711502039  0.3079461881941359
  0.5680528712401444  0.3831972991573525  0.4252226008500266
```

```
Template Cluster: Ti-15-1
   1.00000000000000
    15.3155353988418899    0.0000000000000000    0.0000000000000000
     0.0000000000000000   15.3155353988418899    0.0000000000000000
     0.0000000000000000    0.0000000000000000   15.3155353988418899
   Ti
    15
Direct
  0.6561973133353634  0.5689168351924062  0.5629386886389918
  0.5187192861607723  0.6278829567795587  0.6287414442217051
  0.5415718632911402  0.6523920575971034  0.4503215674015865
  0.6546986973666424  0.5120064380965196  0.4090100979628684
  0.6428422469691006  0.3963616997042423  0.5387938696512943
  0.5614186459860850  0.4613060978376726  0.6650047007551027
  0.3818061849749186  0.6299930721034069  0.5388138423762494
  0.5002256704247823  0.5001728095549847  0.5002333777903570
  0.3767707212039715  0.5691636511371676  0.3842946121974912
  0.5163454323603631  0.5071205234827038  0.3168376936293779
  0.4024210041603928  0.4984455707691027  0.6517700284901786
  0.5635716198294514  0.3626943558246669  0.3993890635964968
  0.3215111966460663  0.4670796149297854  0.5136189694520376
  0.4591521663954429  0.3482429812090265  0.5523931183959199
  0.4027479508954991  0.3982213357816448  0.3878389254403422
```

Template Cluster: Ti-20-1
1.00000000000000
19.7448815559407400 0.0000000000000000 0.0000000000000000
0.0000000000000000 19.7448815559407400 0.0000000000000000
0.0000000000000000 0.0000000000000000 19.7448815559407400
Ti
20
Direct

| | | |
|---|---|---|
| 0.4023818871786118 | 0.4046265580840943 | 0.4273973441394100 |
| 0.3209083450194627 | 0.5005157993151907 | 0.4614895339354397 |
| 0.4079351414235782 | 0.5180950218456919 | 0.3576292669201257 |
| 0.3875083278360639 | 0.4319704842784300 | 0.5608044911326304 |
| 0.4982919050678509 | 0.3596827804932285 | 0.5033896552506377 |
| 0.5214674645250883 | 0.4500753284756843 | 0.3762836291043356 |
| 0.3798185140467033 | 0.6223090590655823 | 0.4376295533102471 |
| 0.4469019464804345 | 0.5206783452177410 | 0.4826149193297807 |
| 0.3627316723964720 | 0.5756414724021964 | 0.5662786007488020 |
| 0.6252754600452994 | 0.3942003749665513 | 0.4476521199336858 |
| 0.5175319204816304 | 0.5965685901123701 | 0.4011007361444757 |
| 0.5538886894458506 | 0.4782612884650401 | 0.5173137080423761 |
| 0.4678545026735186 | 0.6479499100938449 | 0.5242600679379305 |
| 0.6268257510210221 | 0.5241294698813050 | 0.4147359040160576 |
| 0.6820452927123339 | 0.4936663181270384 | 0.5262243653034402 |
| 0.4888351026553856 | 0.5618766068079613 | 0.6220417607172229 |
| 0.5001568988324042 | 0.4239188212354171 | 0.6301013421500328 |
| 0.6135132229073724 | 0.3837466517188557 | 0.5814413730896053 |
| 0.5919678705802212 | 0.6038057363913959 | 0.5274134563776618 |
| 0.6041600846706955 | 0.5082813830223663 | 0.6341981724161094 |

Template Cluster: Ti-5-1

```
   1.00000000000000
    15.4423755336065902    0.0000000000000000    0.0000000000000000
     0.0000000000000000   15.4423755336065902    0.0000000000000000
     0.0000000000000000    0.0000000000000000   15.4423755336065902
   Ti
     5
Direct
  0.5379483679771891  0.3863126595548064  0.5510696865679697
  0.4142228471294765  0.4853975740818610  0.5388821816482874
  0.5673631917619577  0.5418545597971074  0.5483247428205960
  0.5155985236909649  0.4707969030483548  0.4104114457193196
  0.4648670694404118  0.6156383035178631  0.4513119432438274
```

## Supplementary Note 6. Template Structures Set 2

The following structures are the more diverse set of low-energy clusters used in the second step of the two-step process for determining the chemical correlations among elements, as laid out in the main text section titled “Clusters built from low-energy structures of chemically similar elements”. They include clusters of 10, 15, 20, 25, and 30 atoms for the elements of B, Ba, Be, Ca, Cr, Cs, K, Li, Mg, Na, Rb, Sr, and Zn. All structures are provided in the POSCAR format used by the Vienna Ab initio Simulation Package (VASP).

```
Template Cluster: B-10-1
   1.00000000000000
    19.3925122710487088    0.0000000000000000    0.0000000000000000
     0.0000000000000000   19.3925122710487088    0.0000000000000000
     0.0000000000000000    0.0000000000000000   19.3925122710487088
   B
    10
Direct
  0.4875647914128294  0.4402708446251609  0.4090858446476773
  0.5093533559595116  0.5198898130043474  0.4225908128850833
  0.5263696800319977  0.5959069915334112  0.4533093616122912
  0.4757188242769317  0.3836394120334319  0.4673029047626827
  0.5083834214042113  0.4588520831801688  0.4902908520259928
  0.5211400191183131  0.6170242803072412  0.5325703407305724
  0.4955698256894507  0.5393982894759972  0.5096450044626195
  0.4724317126376221  0.4048578021279212  0.5465397403589520
  0.5110410791839612  0.5603209475304070  0.5911359033963492
  0.4924272902851925  0.4798395361819203  0.5775292351177796
```

```
Template Cluster: B-15-1
   1.00000000000000
    20.6387816095120584    0.0000000000000000    0.0000000000000000
     0.0000000000000000   20.6387816095120584    0.0000000000000000
     0.0000000000000000    0.0000000000000000   20.6387816095120584
   B
    15
Direct
  0.5288469494700365  0.5345693511352394  0.3550675862642265
  0.4694504053060484  0.5591766016135864  0.3987874565326521
  0.5853658396846626  0.4898345740750827  0.3746309834326809
  0.5346438487953407  0.5130116985306401  0.4309250418101414
  0.4163060913923431  0.5729371356688517  0.4522833800442783
  0.4733843300955130  0.5270181353840968  0.4851306505091415
  0.5984966620417609  0.4564162171089876  0.4435889161587312
  0.5340228966327984  0.4783481077218070  0.5067082054626084
  0.5966323609518795  0.4287065362182290  0.5152622047206776
  0.4059040329965811  0.5530982165089497  0.5252907044988930
  0.4689303483134069  0.4914361810638823  0.5643664639596082
  0.5521430976104824  0.4368887358753497  0.5763824003971934
  0.3988820788529281  0.5285411233919177  0.5976230870787802
  0.5011688929162119  0.4482234397199534  0.6324771249649430
  0.4358221649400278  0.4817939459834193  0.6414757941654372
```

```
Template Cluster: B-20-1
   1.00000000000000
    21.1888397564775097    0.0000000000000000    0.0000000000000000
     0.0000000000000000   21.1888397564775097    0.0000000000000000
     0.0000000000000000    0.0000000000000000   21.1888397564775097
   B
    20
Direct
  0.5779260017926191  0.4900464884978817  0.3725484037701605
  0.5169099080320265  0.4549793988099822  0.3505201749403994
  0.4497420933890268  0.4316654652008580  0.3657831554848769
  0.6295260183331095  0.5307440114428985  0.4097683737354032
  0.4112329918605582  0.4341178176892768  0.4306790154241824
  0.5970241528974682  0.5162981668374513  0.4810320868019176
  0.4930424856993625  0.4592230732496319  0.4260077130459479
  0.6506654130115805  0.5714545632980901  0.4662945353870924
  0.3713119731923626  0.4456141250884187  0.4952896099404427
  0.5311807255636083  0.4773159219323020  0.4964297283225818
  0.4553696363554423  0.4566443928921515  0.4953614018870525
  0.3370853002526317  0.4670269919185372  0.5599169246656217
  0.4892587925198271  0.4946906015374921  0.5609656398437224
  0.5525884952041480  0.5377346585152765  0.5446744099883034
  0.6210251151407533  0.5802427739844518  0.5362386422137178
  0.4127951697079482  0.4813946013784403  0.5577774806575064
  0.3686732814510592  0.5033422429147407  0.6172118508279070
  0.5795525178207512  0.5872084371342297  0.5982892034469813
  0.5135019396766580  0.5551179405854825  0.6142200559994606
  0.4415879880990587  0.5251383270924208  0.6209915936167434
```

Template Cluster: B-25-1
1.00000000000000
16.1779278591519784 0.0000000000000000 0.0000000000000000
0.0000000000000000 16.1779278591519784 0.0000000000000000
0.0000000000000000 0.0000000000000000 16.1779278591519784
B
25
Direct
0.4927165248494703 0.4712344652720546 0.3812732565586145
0.4391016495812892 0.5620888002719866 0.3981423246376465
0.4082255346530772 0.6345389521042932 0.4713645956077457
0.5515445892703639 0.3870872828020850 0.4126123857693855
0.4942564607779332 0.6458343697393900 0.4219879969082072
0.5513535806549257 0.5641188413041434 0.4062258399853675
0.3903235106286118 0.4724175576352891 0.3973884388871483
0.5961365483844929 0.4745382894798951 0.3929745780959450
0.3576116760578714 0.5389240739829049 0.4676435818525917
0.4855177998076599 0.3163391839119569 0.4590561480395792
0.5761242426593611 0.3177903909075184 0.4933380910632934
0.4370813176452160 0.3846727957941027 0.4090616234809232
0.5515222608257133 0.6130453346376044 0.5041066089322896
0.6145218829334027 0.5200136963456927 0.4846660266747307
0.6179675870204164 0.3855154640868719 0.5514489328184169
0.5031078031752969 0.3703424139903504 0.5510019280627247
0.3440857890750237 0.6051028957192628 0.5428401935515907
0.4316849927171518 0.6210065926118915 0.5903318138685449
0.5296951720789664 0.6303766149587133 0.6067484616413523
0.3881733918396364 0.5209886136246240 0.5787166942635977
0.6514364211484676 0.4757282779354526 0.5748373255743715
0.5990371887785813 0.5596430286885905 0.5896343392422665
0.4486285282766218 0.4417282931823934 0.5991389681231851
0.5506743947664887 0.4523740641749896 0.5997423663079857
0.4894711523939392 0.5345497068379501 0.6157174800525169

```
Template Cluster: B-30-1
   1.00000000000000
    15.8925867069476308    0.0000000000000000    0.0000000000000000
     0.0000000000000000   15.8925867069476308    0.0000000000000000
     0.0000000000000000    0.0000000000000000   15.8925867069476308
   B
    30
Direct
  0.4037046354363270  0.5122554513171309  0.3524668674106834
  0.4003545234092911  0.4143877032385319  0.3717993649824010
  0.5818744108868970  0.5681520265566391  0.3772084969549212
  0.4804137769995265  0.5819813421220630  0.3773959227737791
  0.5069148142246220  0.3443945894774103  0.4067170368601793
  0.3782620622292328  0.5912879130302215  0.4098390288430593
  0.4086122447192011  0.3213524122416800  0.4106923001617986
  0.5991409456515697  0.3830790431102571  0.4134831422739396
  0.6225958775286173  0.4851091159896797  0.4234160580985673
  0.3493535433243693  0.4961901016907717  0.4448658026247898
  0.5436909015429308  0.6344993808938568  0.4486663689355481
  0.4368442127322680  0.6560167206842280  0.4589204412611281
  0.6409969234666733  0.5859014510910967  0.4602932752098567
  0.3569617831091634  0.3902556487018387  0.4711577937033624
  0.6527032532343000  0.4122448897002116  0.4958259174444777
  0.4546209533777628  0.3365804057914517  0.5050442312391681
  0.5587060790307916  0.3673687563009977  0.5112841473234369
  0.6795648443243465  0.5040681014379373  0.5260401309251392
  0.5012202337843519  0.6891748097545829  0.5287890793712009
  0.3168286706109384  0.4586092351373097  0.5370383330422968
  0.5946203083614705  0.6492213458285789  0.5462263971728376
  0.6876044804652111  0.6008379673906674  0.5543403521092429
  0.4035944059472385  0.4080353588342405  0.5704057690718329
  0.4971475112711801  0.3674455013076283  0.5942850597637488
  0.5083053181829911  0.6194853743377919  0.6065933751229906
  0.3677092286459285  0.5028579657682002  0.6117308426663683
  0.6179870747318716  0.5707942385036731  0.6212992240261883
  0.4728223443882626  0.4559117021419468  0.6394075399946502
  0.4394407147001971  0.5568511092442918  0.6560861829653675
  0.5374039236824828  0.5356503383750919  0.6686815176669967
```

Template Cluster: Ba-10-1
1.00000000000000
27.3870349506879087 0.0000000000000000 0.0000000000000000
0.0000000000000000 27.3870349506879087 0.0000000000000000
0.0000000000000000 0.0000000000000000 27.3870349506879087
Ba
10
Direct

| | | |
|---|---|---|
| 0.5547205043800011 | 0.5688329429714170 | 0.3918055213556685 |
| 0.4249113602020947 | 0.4266932935731093 | 0.4083338378023575 |
| 0.5864811598893286 | 0.4116764670205226 | 0.4183429394711826 |
| 0.3985695187853317 | 0.5852583712968172 | 0.4345723750536881 |
| 0.5072719887630448 | 0.6315330910307675 | 0.5463136305204322 |
| 0.4998307490940640 | 0.5002247772835168 | 0.5002439180922783 |
| 0.5048233940008856 | 0.3712447420946932 | 0.5531268589588008 |
| 0.6338023312519933 | 0.5122578983852732 | 0.5371333335566287 |
| 0.3758505905656710 | 0.4881206177302300 | 0.5628375886598902 |
| 0.5137384030675924 | 0.5041577986136817 | 0.6472899965290801 |

```
Template Cluster: Ba-15-1
   1.00000000000000
    30.8607183919292503    0.0000000000000000    0.0000000000000000
     0.0000000000000000   30.8607183919292503    0.0000000000000000
     0.0000000000000000    0.0000000000000000   30.8607183919292503
   Ba
    15
Direct
  0.4706369329689052  0.4796442998637641  0.3648342747915197
  0.5215734635064884  0.6007714127661182  0.4282642086060023
  0.3797649181689228  0.5663695174945059  0.4312016526346704
  0.5944846882203968  0.4779412415924756  0.4403399926604289
  0.6665645967948004  0.5997389773472639  0.4538306804638360
  0.3677428522699948  0.4235026973433166  0.4453245357100937
  0.4988459970902541  0.3687192980277932  0.4514466328588940
  0.4687677340659819  0.4923645577719421  0.5005278352168453
  0.5676902288937533  0.4204347857412610  0.5694717433411479
  0.4255996617015950  0.3867912382405631  0.5726815161533294
  0.5752732650292073  0.5626526598639825  0.5556728430656028
  0.3497317803057518  0.5091097134364155  0.5609987370576344
  0.6948349627134398  0.4882724721729097  0.5401907854434573
  0.4447657633025636  0.6179290096408571  0.5487281567525294
  0.4737231549679733  0.5057581186968243  0.6364864052440156
```

```
Template Cluster: Ba-20-1
   1.00000000000000
    26.3312507681086316    0.0000000000000000    0.0000000000000000
     0.0000000000000000   26.3312507681086316    0.0000000000000000
     0.0000000000000000    0.0000000000000000   26.3312507681086316
   Ba
    20
Direct
  0.4814031051321753  0.4175555422646516  0.3962708419693794
  0.4308299097941721  0.5616786386285524  0.3301033368467248
  0.3187802657598481  0.4633962060685618  0.4153814091649582
  0.3913108755590352  0.6634014119862598  0.5852934810253346
  0.5771066453787824  0.5628339097960050  0.4102937846656075
  0.6473399355291075  0.4117436575054146  0.4108944406323854
  0.4348503191414679  0.5434889490868360  0.4909455085977756
  0.3125905471839912  0.6293596050446360  0.4405376409393859
  0.6999365129934902  0.5302390235088477  0.5240150645392403
  0.4682697441406107  0.6909106803837690  0.4374709769853899
  0.5495046926611051  0.3029265131592878  0.5021311616861432
  0.6817550369827134  0.3725989073281523  0.5782279799740416
  0.5692473081992114  0.4766393593158273  0.2705370281055212
  0.2996267201715253  0.5234387147550768  0.5719199583954471
  0.5572090549824481  0.4590537825047977  0.5364514395920045
  0.5582491739097450  0.6210529266968352  0.5686020687002465
  0.4071624061395470  0.3909417685929472  0.5468026181490874
  0.6262594021628962  0.5015942780129263  0.6729366139398899
  0.4542649421622724  0.5153613070768863  0.6514681303204103
  0.5343034020158339  0.3617848182837367  0.6597165157710480
```

Template Cluster: Ba-25-1
1.00000000000000
32.1222871241219821 0.0000000000000000 0.0000000000000000
0.0000000000000000 32.1222871241219821 0.0000000000000000
0.0000000000000000 0.0000000000000000 32.1222871241219821
Ba
25
Direct

| | | |
|---|---|---|
| 0.3849449877420945 | 0.4935151432781382 | 0.3131079464667526 |
| 0.5016543775951592 | 0.5685082242001334 | 0.3466655634887711 |
| 0.4972345038552204 | 0.4324274527411274 | 0.3732861005016547 |
| 0.3695544770374031 | 0.6174909168368066 | 0.3679326560040981 |
| 0.5996854414373065 | 0.4005242846883127 | 0.4743959696946461 |
| 0.3637184641650769 | 0.3984200931017108 | 0.4121928066860265 |
| 0.2880393240789442 | 0.5138838098217301 | 0.4099626860633390 |
| 0.6235486910601962 | 0.4973406259693610 | 0.3719363918197461 |
| 0.4725090656887490 | 0.6369344237277835 | 0.4633662858708514 |
| 0.4139644095547316 | 0.5171918054977004 | 0.4383059058820154 |
| 0.5400527276234862 | 0.5205679136045127 | 0.4657479534173250 |
| 0.6091941507778509 | 0.6201751673060519 | 0.4268657544981015 |
| 0.6677422193345753 | 0.5160824609464761 | 0.5074686243938411 |
| 0.4651356710401609 | 0.4142243792326583 | 0.5080391509811297 |
| 0.3387422609706799 | 0.6036142830761819 | 0.5023984992919963 |
| 0.5768253399105472 | 0.6034721656363886 | 0.5637215629490417 |
| 0.3402650582520164 | 0.4655421109760635 | 0.5354625302356282 |
| 0.4498683793676104 | 0.5444116609077208 | 0.5656066844351583 |
| 0.6709652616339578 | 0.4021773780128096 | 0.5960077260934962 |
| 0.5616292588153025 | 0.4705738067014583 | 0.5832629304076380 |
| 0.5476784958235101 | 0.3402003215360704 | 0.5943488418927967 |
| 0.6580398484320045 | 0.5257411522214259 | 0.6503547684000650 |
| 0.4506338740947001 | 0.4313545020878578 | 0.6440929969741060 |
| 0.5252381439311915 | 0.5452382395964332 | 0.6849542908667106 |
| 0.5831355677775389 | 0.4203876782950726 | 0.7005153726850575 |

Template Cluster: Ba-30-1
1.00000000000000
27.1940672191650599 0.0000000000000000 0.0000000000000000
0.0000000000000000 27.1940672191650599 0.0000000000000000
0.0000000000000000 0.0000000000000000 27.1940672191650599
Ba
30
Direct
0.5059816968745435 0.6636248374561987 0.2961925068997559
0.6250152559506512 0.3950687520976930 0.2944108424944495
0.4869990772762929 0.4948985541806726 0.3098940416600143
0.6404996815085328 0.5621759699283361 0.3266964452026800
0.3352674504187194 0.4292802306600734 0.3583691764539007
0.3726764301234999 0.5942920471476053 0.3808696684462882
0.4823746836432803 0.3434887362804393 0.3795779864345647
0.6192359047637669 0.7127853872104258 0.4042115442788763
0.7375895048819096 0.4453843098781507 0.4045547705077579
0.5249651972386359 0.5983363340814587 0.4296806491877315
0.5893645229438035 0.4517574809048725 0.4284129130341423
0.6472761984393459 0.3141659858501933 0.4323257046665621
0.4607392361355621 0.7336250946403869 0.4347591788639373
0.4380956539328629 0.4730891081909890 0.4583420453367582
0.6738410058913388 0.5764789048880012 0.4908172333870931
0.2923724044975274 0.4998221989092030 0.5083397418772262
0.5222178113446594 0.3577553500098655 0.5395751337418351
0.3589155169465539 0.3485075201160299 0.5082635303670132
0.4106328977703296 0.6121277305053275 0.5415849573534185
0.6722580202351316 0.4261697752000265 0.5628902935479951
0.4123866243481654 0.4611475567205138 0.6080825349582996
0.5451640687924076 0.5193085092057156 0.5610879516614079
0.5620148527591499 0.6764761006544479 0.5636402183587036
0.3129922515565445 0.5615610040490208 0.6686606574796213
0.5475818858877983 0.4286518367130754 0.6875806537458881
0.2790566992133178 0.4039647078833506 0.6486525670279176
0.4180075230797987 0.3183734460448624 0.6655169833507656
0.4819872836195997 0.5809572099776065 0.6891342691469071
0.6488933446153854 0.5639297930596491 0.6603406179410073
0.3955973153108354 0.4527955275558089 0.7575351825874748

Template Cluster: Be-10-1
1.00000000000000
13.3726471265528293 0.0000000000000000 0.0000000000000000
0.0000000000000000 13.3726471265528293 0.0000000000000000
0.0000000000000000 0.0000000000000000 13.3726471265528293
Be
10
Direct
0.5527956632585216 0.6139816789628441 0.4599687024870249
0.5407879698467895 0.4877813856732445 0.3753186511431039
0.4038907778242447 0.4195824477844852 0.3956146361381698
0.5174656466198897 0.3699058777731281 0.4875045311096855
0.4097053576716689 0.5690376502146464 0.4332075339689823
0.4437829686043955 0.5875520388242634 0.5806593440533518
0.6289997985352898 0.4732931966279850 0.4987972481840899
0.5201012132231781 0.4472294790684117 0.6190626831745476
0.5960439956058181 0.5809053027389623 0.6042769488920925
0.3864266088101900 0.4507309423320081 0.5455897208489304

Template Cluster: Be-15-1

```
   1.00000000000000
    15.2383135772140399    0.0000000000000000    0.0000000000000000
     0.0000000000000000   15.2383135772140399    0.0000000000000000
     0.0000000000000000    0.0000000000000000   15.2383135772140399
   Be
    15
Direct
  0.4994788388258584  0.5000941715783185  0.4995465409562811
  0.6065545397042627  0.5532774568406538  0.4085565154118029
  0.5143036166752566  0.4600591503921976  0.3739628591980306
  0.4737835378246729  0.5957297714515377  0.4075694387108655
  0.5219778717430330  0.3565184876921919  0.4613341685584089
  0.5763048317856416  0.6751050669288361  0.4534642768707045
  0.3990397908097023  0.4218099349033579  0.4543113523913718
  0.6205096743073710  0.4453249984479015  0.4892777191074202
  0.4255142177182023  0.3244476220544357  0.5478353619843332
  0.6005161231462139  0.5769087200080136  0.5463077325964321
  0.3792923908770049  0.5547182474155576  0.5106266345362727
  0.4778761481156550  0.6435658054960385  0.5381757975614434
  0.5267679160015462  0.4056252950600836  0.5924184973073849
  0.4847175689492289  0.5402947772704536  0.6253804563562326
  0.3933629335163431  0.4465204944604366  0.5912326484530297
```

Template Cluster: Be-20-1
1.00000000000000

| | | |
|---|---|---|
| 15.1611250160440107 | 0.0000000000000000 | 0.0000000000000000 |
| 0.0000000000000000 | 15.1611250160440107 | 0.0000000000000000 |
| 0.0000000000000000 | 0.0000000000000000 | 15.1611250160440107 |

Be
20
Direct

| | | |
|---|---|---|
| 0.4562924573056648 | 0.4868268037117787 | 0.3598561646031015 |
| 0.5526920367608454 | 0.3896181202848581 | 0.3714950068406679 |
| 0.3835546940947125 | 0.5886430591422354 | 0.4169120326878826 |
| 0.5888054108776259 | 0.5239400429194419 | 0.3590056072321062 |
| 0.3427947917969831 | 0.4561300959579384 | 0.4307738509252133 |
| 0.5135338819053175 | 0.6213635160461166 | 0.4119815360026493 |
| 0.4464417626268897 | 0.3697900155526170 | 0.4562375488447827 |
| 0.4984339017718824 | 0.4978529540738919 | 0.4972386543932004 |
| 0.6250137174718660 | 0.5713258738365425 | 0.4871772304507189 |
| 0.5740560728387294 | 0.3488912549736654 | 0.5033098563210459 |
| 0.6531017376313812 | 0.4408616941948011 | 0.4438603554282707 |
| 0.5651018861958761 | 0.6898571644920111 | 0.5174694757749378 |
| 0.3350567320028617 | 0.5415566653294945 | 0.5338051598505658 |
| 0.4450891721821293 | 0.6285011386414823 | 0.5378597963996157 |
| 0.3689105924456371 | 0.4137339571176923 | 0.5609751832145804 |
| 0.6159381092407727 | 0.4616235110777041 | 0.5775256739450683 |
| 0.4941748340702290 | 0.3900200816927324 | 0.6028697073591616 |
| 0.5577816983926809 | 0.5852126309884582 | 0.6090832980916900 |
| 0.4359845284852781 | 0.5186558144120781 | 0.6283579686608434 |
| 0.5472419819026371 | 0.4755956055544601 | 0.6942058929739048 |

```
Template Cluster: Be-25-1
   1.00000000000000
    19.6547432930244916    0.0000000000000000    0.0000000000000000
     0.0000000000000000   19.6547432930244916    0.0000000000000000
     0.0000000000000000    0.0000000000000000   19.6547432930244916
   Be
    25
Direct
  0.6561483739477936  0.4934316205827045  0.5856841448739729
  0.6189772814460568  0.4707402189744840  0.4773316713339355
  0.5593071970360342  0.4664183990751383  0.6430459270469271
  0.4632573974524732  0.4991664069090263  0.4623480662658324
  0.6315915830150658  0.5709360083070791  0.5231253922559489
  0.4436476296292753  0.4718982073588478  0.3399861920896967
  0.5995730986952807  0.5708518236522266  0.6209970955868656
  0.4429758754523689  0.4714799991700076  0.5994583994909704
  0.5286165722742240  0.6080072340445213  0.5353773752648934
  0.4316711695252309  0.5657717509095548  0.5485064538386979
  0.5454881929120745  0.4644014112511528  0.3978938551481832
  0.5339435294773835  0.4005223926881740  0.4824027545336946
  0.5621518221072535  0.5606594808568701  0.4441112396805522
  0.5341681164914078  0.4981510403631049  0.5400548691822635
  0.4310077542233302  0.4049867183450502  0.5139226111871977
  0.4988034100647454  0.5565142534481566  0.3596660209689944
  0.4987046904095322  0.5554134874372991  0.6296947016800445
  0.3649468858881662  0.4835300983306254  0.5299316736347919
  0.4552080910037191  0.3995365081149771  0.4116800820233095
  0.3633985661342152  0.5625328565559897  0.4634769524163929
  0.5063973243330366  0.3903468435436324  0.5858710866692292
  0.3667013909741428  0.4614332669008760  0.4233698522363269
  0.6057382188707408  0.4074319373241551  0.5642382790150222
  0.4611261140413205  0.6074999448472340  0.4506744602128236
  0.3964497145951711  0.5583380910090980  0.3671508433634258
```

```
Template Cluster: Be-30-1
   1.00000000000000
    20.1214177847147297    0.0000000000000000    0.0000000000000000
     0.0000000000000000   20.1214177847147297    0.0000000000000000
     0.0000000000000000    0.0000000000000000   20.1214177847147297
   Be
    30
Direct
  0.4896620246333541  0.5106967487478116  0.3042021348707280
  0.4421475980823004  0.4439530694361267  0.3647861205799572
  0.5493635231932572  0.4492029390181287  0.3593537864098266
  0.4353240594445609  0.5557181682311452  0.3774039149171244
  0.5461656742355459  0.5613973238485883  0.3718415635153960
  0.5018570602065182  0.3706649511263365  0.4086626257983568
  0.3817162849819908  0.4859146147198743  0.4336505343974366
  0.4909177852721953  0.6206664797151475  0.4416072721731368
  0.4974066619543365  0.4992393115568703  0.4475949068405782
  0.6118382053591560  0.4975251584088483  0.4219882650032005
  0.5809077651557857  0.4071348507007073  0.4595447757273519
  0.4251282234674179  0.3993241516640111  0.4674919601397676
  0.5806559903940638  0.5794761664450832  0.4743332003951437
  0.4095287904442699  0.5709814852144177  0.4829272487216372
  0.5087729097888953  0.3411514673249201  0.5148336620618181
  0.5439775459394696  0.6573383921868157  0.5295070226643404
  0.4260760415739853  0.3832827775548395  0.5698779171137218
  0.4438266081930706  0.6521939938617720  0.5346531974401145
  0.5044347906598432  0.4432162934962989  0.5326986441352517
  0.4047640051764319  0.4820260561777872  0.5339240813812012
  0.5993807845450192  0.4917797784210620  0.5239730036142660
  0.5917966201087808  0.3916350757242952  0.5614378732798926
  0.4997484425827565  0.5453948266192086  0.5413000080771369
  0.4989854725775942  0.6425221665377876  0.6188667805301762
  0.4188499170813742  0.5686192729419020  0.6009980818961580
  0.5121485808999751  0.3791494799951636  0.6213737328298734
  0.5836355752698879  0.5769706585701257  0.5924989882648077
  0.4528218260499344  0.4683451512189407  0.6271925179586759
  0.5625832775063770  0.4739218939490328  0.6215555691696508
  0.5055779552218169  0.5505572965869590  0.6599206100932236
```

Template Cluster: Ca-10-1

```
   1.00000000000000
    24.2742499665899594    0.0000000000000000    0.0000000000000000
     0.0000000000000000   24.2742499665899594    0.0000000000000000
     0.0000000000000000    0.0000000000000000   24.2742499665899594
   Ca
    10
Direct
  0.4455499896573301  0.5301047982015489  0.3783740308422594
  0.5800525039792318  0.5983565665163723  0.4381094624997885
  0.5803299518089632  0.4420699818691176  0.3979969899399479
  0.4554392042001609  0.3733763501958194  0.4757213838973585
  0.5028200977983489  0.4998114773079578  0.4992221157839509
  0.3574027099485235  0.4940342308373005  0.5036255543511314
  0.5909673095601419  0.4082072657651494  0.5564324145563688
  0.4470970681689010  0.6208370812617829  0.5314205093998509
  0.5839695282912133  0.5637056869525808  0.5946956166369900
  0.4563716365872069  0.4694965610923916  0.6244019220923605
```

```
Template Cluster: Ca-15-1
   1.00000000000000
    26.2694138982467607    0.0000000000000000    0.0000000000000000
     0.0000000000000000   26.2694138982467607    0.0000000000000000
     0.0000000000000000    0.0000000000000000   26.2694138982467607
   Ca
    15
Direct
  0.4297082111755432  0.5999220755816075  0.4315502127741013
  0.5571773641419336  0.6063921594695652  0.5044310065973262
  0.5468581261484118  0.5415397180246183  0.3732963173168228
  0.4109436261244884  0.3619798393118384  0.4598219723468916
  0.4163476347486935  0.4717176227186632  0.3596523370233267
  0.4336584902015211  0.5740403344658563  0.5762235270137833
  0.5474124448823329  0.6325804026371978  0.6427345760400477
  0.5368331172297878  0.3962228973598820  0.3889363399992381
  0.4816358899540426  0.4818733734732344  0.4800419464780811
  0.3446221201441459  0.4915558564154046  0.4851980453973629
  0.6238787776856050  0.4808832357338699  0.4836936659209330
  0.5488286169125950  0.4964985253509372  0.6056535916237000
  0.5373394838338943  0.3674448968293832  0.5311675427857852
  0.4183656195927943  0.4253461296000788  0.5899099119135878
  0.6663904772241964  0.5720029330278631  0.5876890067690191
```

```
Template Cluster: Ca-20-1
   1.00000000000000
    24.1053690895916901    0.0000000000000000    0.0000000000000000
     0.0000000000000000   24.1053690895916901    0.0000000000000000
     0.0000000000000000    0.0000000000000000   24.1053690895916901
   Ca
    20
Direct
  0.4931374795108884  0.3571491934023588  0.3228660670332381
  0.5164231669453551  0.5188011540232100  0.3180894271249645
  0.3933210526751650  0.6021019799820412  0.3792699461370078
  0.5799495693186014  0.4278261110802272  0.4286430355593631
  0.5313024600374190  0.6789162314578003  0.3661012060625573
  0.4175316177808475  0.4484565660071861  0.4178233760966499
  0.6445216603407681  0.5734351798930966  0.4013532593763018
  0.5108108801767304  0.5673077244273069  0.4637682678201737
  0.4810266314661171  0.3183473246569715  0.4807875415534869
  0.5980474722151984  0.6750467613507173  0.5165534618488568
  0.4408442076875899  0.6922873399362336  0.5030558211805587
  0.6275456005059034  0.5204646838971176  0.5536302492873145
  0.6074127786389432  0.3605753106501721  0.5725823440544069
  0.4900886426827972  0.4501534545398837  0.5587711722158799
  0.3556170920833813  0.3890078629627269  0.5530759197554197
  0.3737990531918807  0.5500652850935905  0.5340886258219332
  0.5015810138579480  0.5954002696993359  0.6184568003143478
  0.4704575025482219  0.3312268889072146  0.6467107922465769
  0.5618384512908059  0.4629904883496754  0.6882455862467793
  0.4047436670454455  0.4804401896831407  0.6761271002641834
```

```
Template Cluster: Ca-25-1
   1.00000000000000
    27.0598618499230312    0.0000000000000000    0.0000000000000000
     0.0000000000000000   27.0598618499230312    0.0000000000000000
     0.0000000000000000    0.0000000000000000   27.0598618499230312
   Ca
    25
Direct
  0.5445246696556175  0.4736702266430620  0.3320118416937472
  0.4481636877681404  0.3723972892138505  0.3530534375201593
  0.4059599233800817  0.5085725324852154  0.3643513576803754
  0.5170376207563666  0.5816923789753614  0.4209408657274922
  0.5931580825389506  0.3634468365987227  0.4153676942084357
  0.6232316513264030  0.4932905715426902  0.4538338291727300
  0.3865812880762588  0.6399980787527410  0.4213197584092479
  0.4947347027025509  0.4463648917055100  0.4505507466839273
  0.6251582668127870  0.6176288135145449  0.5168349794148908
  0.2904649827853248  0.5390617352708447  0.4434555171380608
  0.3611027166215682  0.4144324454399619  0.4589657711618182
  0.4084933167446795  0.5366330301482027  0.4993705123744303
  0.3128445716361297  0.4889639437803425  0.5758531314869226
  0.7454441463927018  0.5420334623744231  0.4881853146624653
  0.5328193496423190  0.5257368483787339  0.5521002959213557
  0.4774341874698365  0.6482940861786128  0.5394593875004132
  0.7102057161602757  0.4043753918533795  0.5012576028888660
  0.5791255211902646  0.3960334195923077  0.5511493542300835
  0.4722627132707120  0.3225001031074743  0.4837705248528185
  0.4404644415919767  0.4241506743077464  0.5798981440113468
  0.6595217653898141  0.5032491096203890  0.5919143757249901
  0.3328759974785108  0.6269447718518462  0.5523689778407228
  0.5676009111043790  0.6060806458257455  0.6482021116573564
  0.4295298157348653  0.5544696523163199  0.6350875732970132
  0.5412599537694999  0.4699790605219648  0.6706968947403453
```

Template Cluster: Ca-30-1
1.00000000000000
26.2949945074054909 0.0000000000000000 0.0000000000000000
0.0000000000000000 26.2949945074054909 0.0000000000000000
0.0000000000000000 0.0000000000000000 26.2949945074054909
Ca
30
Direct
0.4535064557176048 0.5220515549641136 0.3304023186680137
0.5032666898574810 0.3834864244376811 0.2986752971054114
0.5971824448220702 0.4858396749021353 0.3585622574466538
0.5566551533143183 0.6312303842657018 0.3549885947574505
0.3691523777667551 0.3937275124313472 0.3575124913470570
0.6027867374640846 0.3480820791520127 0.4121649193050404
0.4616029873305851 0.2903772283923915 0.4067620361849563
0.4835787162757282 0.4206833553736539 0.4243851996497849
0.4165206081885960 0.6441784878469868 0.4219676591734688
0.7176822785887942 0.4449213904599778 0.4420833316946891
0.5154633965088913 0.5525472145017899 0.4525734402858765
0.6534849164875161 0.5802970176278749 0.4604282488746924
0.3721496067955972 0.5066701187500842 0.4535289261523050
0.6003613198760765 0.4558642355363467 0.5019395356192088
0.5389011968447021 0.6804091260821988 0.5008610840710775
0.3822636996469131 0.3692520096108471 0.5077213174604668
0.3049587149318649 0.6161376743732803 0.5233602288651824
0.5249329261726419 0.3366077429334967 0.5363451757239323
0.6727626304767359 0.3554690286690807 0.5516385524963467
0.4019138147942233 0.7240008008612726 0.5520162749758607
0.4732540843237738 0.4658239812624234 0.5541953558597871
0.4357595555223058 0.5952450801380683 0.5546049069439983
0.7036421995249958 0.4963107585065435 0.5804404618288008
0.5768163361769878 0.5680117060662293 0.5868183342847494
0.3450314574610639 0.4918895665249157 0.5989888195721362
0.5792711232962908 0.4303466774409163 0.6397130605040789
0.4984649787321777 0.6718048070087503 0.6483840271575159
0.4337780877325014 0.3838055894745750 0.6510499984894721
0.3582918686813270 0.6271658042282368 0.6599645865292345
0.4665636366873891 0.5277629681770823 0.6779235589727373

Template Cluster: Cr-10-1

```
   1.00000000000000
    15.0753659406382408    0.0000000000000000    0.0000000000000000
     0.0000000000000000   15.0753659406382408    0.0000000000000000
     0.0000000000000000    0.0000000000000000   15.0753659406382408
   Cr
    10
Direct
  0.6048948939328974  0.5537214363164809  0.3612838015418336
  0.4718220234296503  0.4410352417157222  0.3799323322734349
  0.6513695084973173  0.4547253076131043  0.3653831412003496
  0.4577982237552692  0.5772336053174210  0.4748638874149576
  0.5241195837043177  0.3696259483591015  0.4592290100518875
  0.5758853823678175  0.5138281102280690  0.5251543455658012
  0.3782391308609749  0.4435585151105632  0.5343476578392975
  0.4586740726134428  0.4362886762789689  0.6164512953750371
  0.3850860698647279  0.5962884079920410  0.6367265445924142
  0.4921111109735851  0.6136947510685353  0.6466279841450010
```

Template Cluster: Cr-15-1
1.00000000000000
19.8718709208916806 0.0000000000000000 0.0000000000000000
0.0000000000000000 19.8718709208916806 0.0000000000000000
0.0000000000000000 0.0000000000000000 19.8718709208916806
Cr
15
Direct
0.4255024531917372 0.5287874427106486 0.4018937871986239
0.5427643073428642 0.5563262675634244 0.3835411045071579
0.4937984027673027 0.4294819812174213 0.3833014312523665
0.3797673618969074 0.4436290280818369 0.4692551679522727
0.5978222745879981 0.4623895583227282 0.4487429699011537
0.4488356495706063 0.6224349104415149 0.4693734848847769
0.5684029918101393 0.6055702963206078 0.4873140030017956
0.4796145775281953 0.3760120811209824 0.4875184159793240
0.4998762622364367 0.4999139343780202 0.5000263438512711
0.6217146301010934 0.5257229725173614 0.5556425938949238
0.5726187495142766 0.3988594517709827 0.5558882729672385
0.4023765791524828 0.5379714588515971 0.5513715551430078
0.5048027089638892 0.6009157381688627 0.5914072924369435
0.4355385111023125 0.4220919164780187 0.5915242530389092
0.5265645402337441 0.4898929620559783 0.6231993239902348

Template Cluster: Cr-20-1
1.00000000000000
20.9883781889631891 0.0000000000000000 0.0000000000000000
0.0000000000000000 20.9883781889631891 0.0000000000000000
0.0000000000000000 0.0000000000000000 20.9883781889631891
Cr
20
Direct
0.5819618279337926 0.6056125816210181 0.3985210133324947
0.4675811891404958 0.4773359469436719 0.3448033139429234
0.4649952344634741 0.5547591136210416 0.3304810559736684
0.5938825905535016 0.5334888213692324 0.3704400198709999
0.4982185460116135 0.5296037012729324 0.4468093542432583
0.3941920755443934 0.5095161403633178 0.4623217560310622
0.4541793895373636 0.6345500028876618 0.4321130148122491
0.5569421236879015 0.4298505615210937 0.4357963396096999
0.4469217136062710 0.4077988773905348 0.4534139721522790
0.6124531833332313 0.5090110770857923 0.5041243630933402
0.5449196389016738 0.6014598630885220 0.5243602580544052
0.5530908299933190 0.3842215207058277 0.5385894600166941
0.4467411660809004 0.3629391792878917 0.5667178325164932
0.4956908751218639 0.4787620841826581 0.5439911313709844
0.4330988308823873 0.5867720503233881 0.5436158423512319
0.5989894438464154 0.4510843304042734 0.6133869230285047
0.3887853638577005 0.4576462656843192 0.5674848192018374
0.5409616476966990 0.5437734663717871 0.6220281293404073
0.4922943743829253 0.4218694581684244 0.6534768146035590
0.4340999554241345 0.5199449577065967 0.6475245864539074

Template Cluster: Cr-25-1
1.00000000000000
22.1108912946558185 0.0000000000000000 0.0000000000000000
0.0000000000000000 22.1108912946558185 0.0000000000000000
0.0000000000000000 0.0000000000000000 22.1108912946558185
Cr
25
Direct
0.4810715133660395 0.5325986252236063 0.3251559132076238
0.4643661084217001 0.4370665257813472 0.3701141429750550
0.5650067391367058 0.5106957851495652 0.3964148303805288
0.5509921684770304 0.4213186094791936 0.4488865813116191
0.3876729037606808 0.5409095525910386 0.3748301734818896
0.4896890386291233 0.6160341133818433 0.3983969589444031
0.6565123374231447 0.4975467642964019 0.4652432430004509
0.4690934002142911 0.5183011111620174 0.4469612282737998
0.4537921668046323 0.4208025447107673 0.4970201614881163
0.3675800492785939 0.4437990400522765 0.4319792518058633
0.5712837210130247 0.5900015477071037 0.4756373891615339
0.3978413036258815 0.6191017355285524 0.4495234546095334
0.4748701670355189 0.5973587105674768 0.5247391337436795
0.6674242548782985 0.5360899056711259 0.5307583340832024
0.3530484879879694 0.4184597016373673 0.5622117086128070
0.5492315908811704 0.4975940402029409 0.5267887746521677
0.3701580153876378 0.5237895594539311 0.5009805291961342
0.4466202031609968 0.4959345807075763 0.5717920971658755
0.6316319358385413 0.4086446027158033 0.5383020588523660
0.5272397556885124 0.3900016860333083 0.5721841031349869
0.4063986116298395 0.3858550117452210 0.5993327208221614
0.5816889013205824 0.5975861256319276 0.5956234190169454
0.5216700514023386 0.5696493294530713 0.6293087776266832
0.6097002706007969 0.4791302819983513 0.6134516175052127
0.5054163040369346 0.4517305091181924 0.6543633969473672

```
Template Cluster: Cr-30-1
   1.00000000000000
    22.0745156950589916    0.0000000000000000    0.0000000000000000
     0.0000000000000000   22.0745156950589916    0.0000000000000000
     0.0000000000000000    0.0000000000000000   22.0745156950589916
   Cr
    30
Direct
  0.4335099594388785  0.4634969220836719  0.3330406705838800
  0.4599238376721572  0.5818487872418809  0.3460982288025337
  0.3918860462569201  0.4193807490858693  0.3775435743435196
  0.5333861802871906  0.5094595096542438  0.3855137435413363
  0.5671712816811755  0.6287434205066197  0.3960107142962286
  0.3702000708463692  0.5446394858217355  0.3977611096707266
  0.5118186303850344  0.3928318850867998  0.4032520248093790
  0.4674908894952040  0.6058442394654301  0.4511634057317173
  0.6241910219992599  0.4589202050104198  0.4270450951869952
  0.4492594648845790  0.4889350414535698  0.4521270114391912
  0.5803727921815195  0.6581203138755257  0.4640538946597127
  0.6093878584476021  0.3544480434830218  0.4459292226858744
  0.4423157385761877  0.3736220584078936  0.4781618119784986
  0.5593177632318397  0.5509102547144755  0.4860630737937366
  0.3805426882153031  0.5760242271127688  0.5104391901880951
  0.6498106487519609  0.5067562639873487  0.5214260106204814
  0.4733841453479847  0.5328973941273655  0.5565375148239909
  0.5440757934744679  0.4422894025465880  0.4997730945097444
  0.6386866114058320  0.3972169611183979  0.5409425929682770
  0.5319917720775960  0.3342546757036828  0.5175478361229252
  0.4571710021259849  0.4282588306803226  0.5723049236142611
  0.3656329736141288  0.4743068697881263  0.5102444828373610
  0.5830910304057849  0.6007482207276574  0.5803661292867137
  0.4903305230153180  0.6389066629135065  0.5534371544953312
  0.3948418835402342  0.5873254120370799  0.6294142797398325
  0.5078472685866799  0.5673213854800552  0.6634225604476666
  0.5719592836812364  0.4912075123133082  0.5957402914723751
  0.5527480074412620  0.3874098788696981  0.6138017889870074
  0.4832597535147752  0.4912660509315434  0.6713610972830550
  0.3743950794175474  0.5126093357713720  0.6194774710795595
```

Template Cluster: Cs-10-1

```
   1.00000000000000
    31.5426697906026199    0.0000000000000000    0.0000000000000000
     0.0000000000000000   31.5426697906026199    0.0000000000000000
     0.0000000000000000    0.0000000000000000   31.5426697906026199
   Cs
    10
Direct
  0.4815799102440547  0.4384517667878591  0.3542848310675933
  0.4853985715902195  0.5957380916339143  0.3727075309195739
  0.3875823076068448  0.4871399890933661  0.4688149294922647
  0.6163156378562124  0.5216483544638539  0.4743371920763413
  0.3912483738731671  0.3186113607658537  0.4950506034741361
  0.6203536807468955  0.6868269457021824  0.5027358504525143
  0.5360325599348507  0.3963557782612604  0.5098800252109108
  0.4767937045929344  0.6018193533508401  0.5437866462681615
  0.4243622189363639  0.4575365180452109  0.6337797168704375
  0.5803330346184707  0.4958718418956589  0.6446226741680665
```

Template Cluster: Cs-15-1

```
   1.00000000000000
    32.0332126225528100    0.0000000000000000    0.0000000000000000
     0.0000000000000000   32.0332126225528100    0.0000000000000000
     0.0000000000000000    0.0000000000000000   32.0332126225528100
   Cs
    15
Direct
  0.5006709535559788  0.4536401520573471  0.3350349638536194
  0.5531135246644545  0.6033372599880398  0.3665144815416640
  0.6432990148716423  0.4716314177058179  0.4152057880821806
  0.3917409491871618  0.5611271551165662  0.4199380989205803
  0.3944514104355478  0.3762346987891277  0.4259236484750313
  0.5525867950524991  0.3393860338295391  0.4439332117557841
  0.5019602356182161  0.6822816348502031  0.4997958195355653
  0.5007533161874734  0.5010950987007434  0.5001326627501114
  0.6453953865311375  0.6076260640848562  0.5001456975005657
  0.3427658454813238  0.4557086057973425  0.5545700789755184
  0.4537775716377310  0.3373895165085920  0.5711357198876325
  0.6075194066366314  0.4363445931539290  0.5804047236678923
  0.3951583909657061  0.6049334988307620  0.5903682962370255
  0.5541958657936974  0.5995181363558540  0.6325317373472288
  0.4626113333807850  0.4697461342312799  0.6643650714696152
```

```
Template Cluster: Cs-20-1
   1.00000000000000
    33.8497366188743101    0.0000000000000000    0.0000000000000000
     0.0000000000000000   33.8497366188743101    0.0000000000000000
     0.0000000000000000    0.0000000000000000   33.8497366188743101
   Cs
    20
Direct
  0.4131571826808330  0.4238850459497868  0.3474236860753718
  0.5152827493456154  0.5425132928111578  0.3614052207199821
  0.6615542559263474  0.5052856225725416  0.3978722739742046
  0.4652823804047018  0.6869211809175988  0.3887753923135906
  0.5535087109659386  0.3875255073371173  0.4077546292462281
  0.3630129238216439  0.5700699519488334  0.4085969341810606
  0.5957640177151190  0.6454616212029233  0.4626686876388382
  0.3035075077852925  0.4296749221447839  0.4661272623115562
  0.4218958939292229  0.3214288504510615  0.4672608333105022
  0.4397579902747301  0.4746593793052024  0.4918087659498335
  0.6688935671101091  0.3980680018525463  0.5160162244606016
  0.5624388183519010  0.5116544591798278  0.5164964059574767
  0.6997448156621906  0.5513386439973229  0.5453330672834587
  0.4509492957447465  0.6351324551670265  0.5341579338657566
  0.5245804067581699  0.3704886167806251  0.5752786144477015
  0.3300825063171615  0.5463819242947962  0.5716913749202953
  0.3727436311223535  0.3965188898260819  0.6079862157133645
  0.5778899961066956  0.6195940997555323  0.6265930908055615
  0.4642444910051448  0.5156391253968685  0.6519708509731041
  0.6157088589720761  0.4677584091083657  0.6547825358514904
```

Template Cluster: Cs-25-1
1.00000000000000
38.0390969582983800 0.0000000000000000 0.0000000000000000
0.0000000000000000 38.0390969582983800 0.0000000000000000
0.0000000000000000 0.0000000000000000 38.0390969582983800
Cs
25
Direct
0.6111730098264315 0.4807874409052686 0.3416018255687097
0.3456190842005814 0.5349848022175601 0.3850887399501018
0.4812812474704628 0.4978409716063397 0.3929284245512558
0.5340157864574815 0.3680899221057898 0.4045637899609701
0.5810916824029276 0.5986173130742072 0.4228597507825173
0.6898535155877471 0.3894842895298130 0.4113947107008707
0.3905241946638598 0.3964257087077422 0.4320026043676474
0.4414283793860100 0.6271816018444087 0.4433393465460970
0.7091730402006117 0.5309004549725298 0.4269337352940620
0.2673442044151396 0.4719738830578393 0.4820333449889437
0.3054431606643961 0.6122508367537340 0.4932434780744996
0.5939204916018094 0.4655429757651374 0.4742651743119808
0.3966628993208871 0.5061432943248337 0.5067522981867044
0.5120637995690168 0.5389857770364291 0.5291603950644763
0.5395012388179371 0.6694926001636193 0.5397552525494961
0.4840287486820753 0.3886417952332341 0.5353321118794799
0.6185445119395744 0.3428815591127799 0.5215261914518872
0.7140228673188099 0.4469394044278889 0.5370382351581019
0.6475439082263604 0.5718338887240170 0.5508576471102438
0.3468023721892508 0.3990291714827794 0.5722876901192420
0.4130206501509133 0.6195945683776444 0.5880329047767247
0.3044080634367513 0.5291296237355356 0.6043959752364060
0.5853537194718037 0.4512425349604711 0.6139342207777108
0.4445572628625257 0.4799001608479636 0.6385331223038716
0.5426221611366429 0.5821054210324270 0.6521390302879780

Template Cluster: Cs-30-1
1.00000000000000
37.2825642353942115 0.0000000000000000 0.0000000000000000
0.0000000000000000 37.2825642353942115 0.0000000000000000
0.0000000000000000 0.0000000000000000 37.2825642353942115
Cs
30
Direct

| | | |
|---|---|---|
| 0.5382917129100131 | 0.4824130193940494 | 0.2755930735455301 |
| 0.5080182520330263 | 0.6079064547286451 | 0.3291716206661147 |
| 0.4090585246420347 | 0.4991771280574402 | 0.3363922005665073 |
| 0.4767685843199601 | 0.3771732275572418 | 0.3562674151930210 |
| 0.6206766813239273 | 0.4084490321134229 | 0.3608398206602779 |
| 0.6386924724770412 | 0.5555322042873900 | 0.3649587690282315 |
| 0.3292776632359771 | 0.3868898214223485 | 0.4080835285495229 |
| 0.5261506994718622 | 0.4972079327393896 | 0.4177084953756943 |
| 0.3143314666471021 | 0.5366886164301214 | 0.4264422553026084 |
| 0.5755824063493277 | 0.6642559050895332 | 0.4461779319764492 |
| 0.4422505505413391 | 0.5989804607806108 | 0.4538201170240219 |
| 0.4368558409196110 | 0.3060528466830320 | 0.4687357717667323 |
| 0.7078254115313776 | 0.6124062178852797 | 0.4760964223930036 |
| 0.6791274315087211 | 0.4675674748169376 | 0.4778450623584025 |
| 0.5577677223301399 | 0.3837863171595434 | 0.4881875538983249 |
| 0.4156512355127165 | 0.4491385276150802 | 0.4854227750966194 |
| 0.5853336796060079 | 0.5533640362110094 | 0.5214608972349556 |
| 0.2749858731681501 | 0.4603864606518275 | 0.5311672323597090 |
| 0.3390338271339334 | 0.3434211498774142 | 0.5560854741214803 |
| 0.3591203392449673 | 0.5760336715829222 | 0.5624107701805748 |
| 0.4822519879855833 | 0.5049889386848407 | 0.5782803400884251 |
| 0.6342146325548335 | 0.6710428949302559 | 0.5825448214698048 |
| 0.4924914640556584 | 0.6514306913752435 | 0.5763124727404545 |
| 0.7024761291961082 | 0.5431435269067689 | 0.6004432939079318 |
| 0.4757306039008617 | 0.3629977900690036 | 0.6007548627012320 |
| 0.3672451804848699 | 0.4545054239264826 | 0.6429877632700058 |
| 0.5760439947266793 | 0.5704984818883031 | 0.6693562397075751 |
| 0.6054496429851475 | 0.4380067878913154 | 0.6108749833531665 |
| 0.4982526692777911 | 0.4553197581803882 | 0.7093452749730554 |
| 0.4310433199252028 | 0.5812352010641663 | 0.6862327604905741 |

Template Cluster: K-10-1

```
   1.00000000000000
    27.0075030256539215    0.0000000000000000    0.0000000000000000
     0.0000000000000000   27.0075030256539215    0.0000000000000000
     0.0000000000000000    0.0000000000000000   27.0075030256539215
   K
    10
Direct
  0.4584900212937625  0.6389281640323867  0.3520268247239046
  0.4631664270381858  0.4669498877154943  0.3800381110778939
  0.5922109397803861  0.5656675693814586  0.3968275890436354
  0.3632589675677418  0.5419851486842954  0.4720829998788690
  0.6035633498105990  0.4158803005634008  0.4584632322633576
  0.4528051423770950  0.3806254978978312  0.5208647896553255
  0.4946954576934807  0.6155944240101380  0.5293436261999008
  0.5956902135935128  0.5125441796207298  0.5928836431605745
  0.4321308121277785  0.4946641564601919  0.6304698235986209
  0.5439886687174508  0.3671606716340802  0.6669993603979181
```

Template Cluster: K-15-1
1.00000000000000
24.3768177084362989 0.0000000000000000 0.0000000000000000
0.0000000000000000 24.3768177084362989 0.0000000000000000
0.0000000000000000 0.0000000000000000 24.3768177084362989
K
15
Direct
0.4383371201312219 0.5322103694870393 0.3148135187584412
0.5960778175434114 0.4379235106946592 0.3453054097190817
0.5782675502028998 0.6233848315590425 0.4102628273774256
0.4383814484959885 0.3596423083935310 0.3929721264635266
0.3788261638922858 0.6574314282652999 0.4366332385235284
0.3126923343879384 0.4870053220086134 0.4389626238276637
0.5923032162886755 0.3020774271222327 0.4704459586075199
0.7021751423419373 0.4399650449656013 0.4992424725566957
0.5015304264660619 0.5013022603171653 0.5001370000461237
0.4945602499973404 0.6851194023650985 0.5778964257642131
0.3387835366616953 0.5817253914517118 0.5939433916206518
0.5949869547857833 0.3930676881479896 0.6368484508849922
0.4069432143167281 0.3905337539765787 0.5887127609493439
0.6414671195052229 0.5760063001002796 0.6046597654165123
0.4846677049828023 0.5326049611451503 0.6891640294842806

```
Template Cluster: K-20-1
   1.00000000000000
    26.5328496187824001    0.0000000000000000    0.0000000000000000
     0.0000000000000000   26.5328496187824001    0.0000000000000000
     0.0000000000000000    0.0000000000000000   26.5328496187824001
   K
    20
Direct
  0.5420598243835364  0.5427843027939223  0.3001660371972559
  0.4132441729938693  0.4424981505184562  0.3187973031964673
  0.5685279141617045  0.3761883076943823  0.3321687654200609
  0.4018170600442266  0.5994306854037179  0.3849413835219738
  0.5578877137202520  0.6429532280024089  0.4407494242733215
  0.6560950472252040  0.4996240567022456  0.4150326682590740
  0.4411585718886549  0.3421670271354313  0.4534790421095676
  0.4301040367022428  0.7509323776897332  0.4591165720349802
  0.3301457150709989  0.4706826522912007  0.4723272058204593
  0.6002092192031279  0.3596462145351408  0.5012665579167603
  0.4995627129177102  0.4972304903303850  0.4968503567922969
  0.5284214361064512  0.6982700645794925  0.5968783872044194
  0.7456195847942092  0.4317760674254830  0.5424724007873152
  0.3149734587078824  0.3310763291895138  0.5707642878553278
  0.3428862015114547  0.4825731111334827  0.6425749922807015
  0.3868845334450733  0.6157268605426127  0.5551147472864941
  0.6263814349739969  0.4285619023044103  0.6571596231822702
  0.6430362840379820  0.5713266159074879  0.5707744630739799
  0.4709640099007019  0.3737382772557790  0.6228078098842755
  0.5000210682107351  0.5428132785647065  0.6665579719030269
```

```
Template Cluster: K-25-1
   1.00000000000000
    30.0149444182593612    0.0000000000000000    0.0000000000000000
     0.0000000000000000   30.0149444182593612    0.0000000000000000
     0.0000000000000000    0.0000000000000000   30.0149444182593612
   K
    25
Direct
  0.5732220881023241  0.4765810646491538  0.3235150036696005
  0.4245240513555203  0.4624153022108532  0.3635306833345470
  0.6679747368360075  0.5991803924163405  0.3787294292380420
  0.4995274227777469  0.5888642020174448  0.3761808310720328
  0.6821063829808953  0.4379064894952614  0.4056142423964698
  0.3502512524538579  0.5864148351375282  0.4001422356809171
  0.5383607007673931  0.3869593629805227  0.4364403829791612
  0.5680348727116417  0.6993308625934835  0.4502277374064517
  0.2763342500113357  0.4507480456365260  0.4137469679610205
  0.7370770276747595  0.5336351004835022  0.5015395139117577
  0.5855632202552336  0.5488534416055433  0.4883281671776070
  0.3858160498711600  0.3643345357495479  0.4750662385997735
  0.4376186218378166  0.6373400230332598  0.5083906334367535
  0.4635130099985473  0.4864833778237287  0.5117513016299533
  0.3250578816806872  0.5242954625136900  0.5370722949065871
  0.2551570305011268  0.3878999633133610  0.5495677753711262
  0.3505163954520648  0.2758131193477795  0.5878768075857838
  0.6866740851967033  0.6640324757437193  0.5258170877850763
  0.4994940365034827  0.3306321957452604  0.5739802951227745
  0.6311521008517358  0.4147569483327280  0.5491753039418161
  0.3871230368580464  0.4162642829484910  0.6254147206133576
  0.4495894583287706  0.5629016697039336  0.6376965319499391
  0.5505744379981726  0.6644969090292245  0.5990718934098769
  0.6384096243065420  0.5505288301231162  0.6242749426064353
  0.5363282246884141  0.4493311073659648  0.6568489782131396
```

Template Cluster: K-30-1
1.00000000000000
30.0880677634890006 0.0000000000000000 0.0000000000000000
0.0000000000000000 30.0880677634890006 0.0000000000000000
0.0000000000000000 0.0000000000000000 30.0880677634890006
K
30
Direct

| | | |
|---|---|---|
| 0.4742765549737480 | 0.5376304110753181 | 0.2930984955502961 |
| 0.3877138847361244 | 0.4249012831486962 | 0.3295151166203522 |
| 0.5492547570887530 | 0.4142389195769399 | 0.3419755412907906 |
| 0.4627897826880178 | 0.2978082087361071 | 0.3404269767508923 |
| 0.6019148113190029 | 0.5603131242357423 | 0.3794955610338940 |
| 0.4778765299838506 | 0.6599382081181177 | 0.3990815726463412 |
| 0.3510866154818686 | 0.5688049562513892 | 0.4012434219085362 |
| 0.3394534537402640 | 0.3007513209971652 | 0.4184559010206217 |
| 0.6325311529636937 | 0.6969214995190398 | 0.4264766451312824 |
| 0.2919811941429160 | 0.4378446415447309 | 0.4409826403337285 |
| 0.4763851412740048 | 0.5139205683032634 | 0.4376971816816196 |
| 0.5712983029963240 | 0.2993934037624701 | 0.4415275893003150 |
| 0.4514814813247265 | 0.3856164134788648 | 0.4755514655316982 |
| 0.6230463661200345 | 0.4383778340539234 | 0.4730889158531332 |
| 0.5138350499226588 | 0.7615473478105280 | 0.5011344251439156 |
| 0.6878285832339884 | 0.5751512538480260 | 0.5130084174412434 |
| 0.5491030731393278 | 0.6139706867556980 | 0.5244342836438228 |
| 0.4502817665854701 | 0.2587595219382307 | 0.5368345637883077 |
| 0.4049724122290829 | 0.6486123897427103 | 0.5363608127081789 |
| 0.3743814580534111 | 0.5001599913196486 | 0.5499869955483327 |
| 0.5700079425117828 | 0.3419433020770119 | 0.5866769534234956 |
| 0.5292056193407328 | 0.4848746612682875 | 0.5747133313204651 |
| 0.3272333314836255 | 0.3507400243541292 | 0.5613415419887400 |
| 0.6493893420132261 | 0.7125103097563781 | 0.5739990696937698 |
| 0.6733175306318492 | 0.4647510144335085 | 0.6133958545621794 |
| 0.5115141618790153 | 0.7071955350868729 | 0.6379290347233830 |
| 0.4382112341185567 | 0.3988841229655045 | 0.6467358053440188 |
| 0.4540153175973885 | 0.5727649843596910 | 0.6632430105140342 |
| 0.6137704152065847 | 0.5974143098231343 | 0.6626123411186551 |
| 0.5618427332199704 | 0.4742597516588654 | 0.7189765343839638 |

Template Cluster: Li-10-1
1.00000000000000
22.6178070760780301 0.0000000000000000 0.0000000000000000
0.0000000000000000 22.6178070760780301 0.0000000000000000
0.0000000000000000 0.0000000000000000 22.6178070760780301
Li
10
Direct
0.3997720531959104 0.4402406468449682 0.4377035033243643
0.5712318852871573 0.5711072678110642 0.4139037168578414
0.5350456556411517 0.4550203056555601 0.4460024855337419
0.6615372623167495 0.4813065542184135 0.4681750737890557
0.3387777421619127 0.5181537875242531 0.5324958505810944
0.4484698778386557 0.5512739993664812 0.4701018831171084
0.5618314489746433 0.5338824456874427 0.5340786188108979
0.4545530769572794 0.4601540350239946 0.5488033680705284
0.4480077089599419 0.5776381407058266 0.5938900180245619
0.5807732886666056 0.4112228171619954 0.5548454818907984

```
Template Cluster: Li-15-1
   1.00000000000000
    18.4525398626503900    0.0000000000000000    0.0000000000000000
     0.0000000000000000   18.4525398626503900    0.0000000000000000
     0.0000000000000000    0.0000000000000000   18.4525398626503900
   Li
    15
Direct
  0.5236522886029492  0.4271932175339622  0.3130451141786906
  0.4038179087996152  0.4890662929974182  0.4128431455961149
  0.5714107536421194  0.5572474453383864  0.4066364925383149
  0.4340356469319367  0.6574329213391443  0.4351659038834317
  0.4556061297931500  0.3136185115806333  0.4271853643867624
  0.6572181902803280  0.3955798055202143  0.4208738130059698
  0.5276502445669843  0.4278663806137086  0.4616619586803466
  0.4760628709417025  0.5603480417813306  0.5312813219226378
  0.5736081180420108  0.3214649333372231  0.5546775795532066
  0.3378583869412214  0.5587941528902177  0.5603368910160087
  0.5792109897814945  0.6571232975094304  0.5525376925606438
  0.4379077876124581  0.4153854705550427  0.5771433081269317
  0.6077299692746524  0.4835119716083855  0.5718957519186445
  0.4284199963076906  0.6859340218210001  0.5994986932216023
  0.4858107184817080  0.5494335355739026  0.6752169694107011
```

Template Cluster: Li-20-1
1.00000000000000
18.9217855454327299 0.0000000000000000 0.0000000000000000
0.0000000000000000 18.9217855454327299 0.0000000000000000
0.0000000000000000 0.0000000000000000 18.9217855454327299
Li
20
Direct

| | | |
|---|---|---|
| 0.4628427054104028 | 0.4760279809662489 | 0.3272252853490521 |
| 0.7319916376257415 | 0.5525106087344056 | 0.4645837235196454 |
| 0.3605880040359850 | 0.6253903077507608 | 0.3926742543986523 |
| 0.3467638604644766 | 0.4640655764414845 | 0.4316761375640413 |
| 0.5363939902224560 | 0.6520834326567464 | 0.3759014806712513 |
| 0.5994153628066774 | 0.5096766558243875 | 0.3951007359186875 |
| 0.4907882784229990 | 0.4202937314412425 | 0.4603656781946218 |
| 0.5928972886961024 | 0.3491080384003395 | 0.3957211354949926 |
| 0.4641753256878740 | 0.5587707365837040 | 0.4563766597367796 |
| 0.4529968282452403 | 0.6864657421005979 | 0.5145375000811604 |
| 0.3824911330837974 | 0.3932094317115654 | 0.5643154708833134 |
| 0.6325524528335505 | 0.4443503923320467 | 0.5194933808287630 |
| 0.5301848117311284 | 0.3426094586974283 | 0.5791134764458484 |
| 0.4182964074008199 | 0.3119939232049549 | 0.4185642926146441 |
| 0.4968397207327795 | 0.4916776882917099 | 0.5816171312088773 |
| 0.4912324150206248 | 0.6097327010333381 | 0.6578869041283416 |
| 0.3636405754602040 | 0.5588250066732989 | 0.5673390720071214 |
| 0.5927348090438479 | 0.5963311799589526 | 0.5266801299142272 |
| 0.4420273296789120 | 0.4599006157906804 | 0.7043498083456065 |
| 0.6111470633963663 | 0.4969767914061216 | 0.6664777426943729 |

```
Template Cluster: Li-25-1
   1.00000000000000
    20.2743004489877805    0.0000000000000000    0.0000000000000000
     0.0000000000000000   20.2743004489877805    0.0000000000000000
     0.0000000000000000    0.0000000000000000   20.2743004489877805
   Li
    25
Direct
  0.5868318301324523  0.4600937070168524  0.2614896755307678
  0.6494446847782970  0.5804000645631562  0.3289303653918360
  0.5149587141314246  0.5966875452955477  0.2670780322114836
  0.4080448742084674  0.5784715137338808  0.3830840311626339
  0.6591901438697836  0.4443922219254212  0.3965878410277013
  0.4244362304878460  0.4443127236351539  0.4420473263952127
  0.5308772823614351  0.3663540064312681  0.3801618362368979
  0.5384222224169720  0.6521017889313110  0.4061078791106199
  0.5398191377281406  0.5144578527849561  0.3758863349549444
  0.4131660842883539  0.6761466279794004  0.5026404408939604
  0.4844158536567801  0.5620046758736951  0.5041811660499362
  0.6323892232599148  0.5606698937919240  0.4855249995653741
  0.5553902224442744  0.4382984216982260  0.5017687841825086
  0.4660605243513027  0.3298568731543249  0.5212393305554058
  0.3453234203433064  0.5411756843315439  0.5219764428025273
  0.3245563652377516  0.3910685731583883  0.5439965775057831
  0.5545193293296756  0.6650204376168972  0.5678932241656378
  0.6803978392959913  0.4361269865388747  0.5609819958637434
  0.4377271961076373  0.4549285932671935  0.5921578064950922
  0.5703217163687599  0.5242447252451160  0.6189009980744637
  0.5515013232769286  0.3804823466824795  0.6359815196442137
  0.4337172501792474  0.5923910726506526  0.6350994006929565
  0.4017641432614328  0.3511325508363277  0.6704840781343359
  0.3285945542491597  0.4847872735922715  0.6691421454500325
  0.4681298342346936  0.4743938392651444  0.7266577679019672
```

```
Template Cluster: Li-30-1
   1.00000000000000
    21.7938455404225415    0.0000000000000000    0.0000000000000000
     0.0000000000000000   21.7938455404225415    0.0000000000000000
     0.0000000000000000    0.0000000000000000   21.7938455404225415
   Li
    30
Direct
  0.5178506968555534  0.5576148731308054  0.3288474999715432
  0.4927073294899159  0.4223008175740664  0.3658478173059594
  0.6336560484993489  0.4593102483785942  0.3350326410001673
  0.3966864273517556  0.6391694652789388  0.3701607991214832
  0.5271389564417293  0.6421623982006843  0.4425396081794459
  0.3760830771082320  0.5048673096530139  0.3225895134109024
  0.6556294389143160  0.5939563396484759  0.3819453323127761
  0.5760210651301324  0.5112525533086405  0.4347888557091451
  0.4881047713247071  0.4321170993040693  0.4971019278277745
  0.6038622353736827  0.3782735875312299  0.4463280700555243
  0.4383858707683033  0.5353766350070759  0.4278474086856527
  0.3661905357222043  0.4196941818848586  0.4346321430830467
  0.7021672439764720  0.4816752256497738  0.4598902480074004
  0.3995813997031431  0.6360938002085069  0.5108012221478702
  0.3075553763292464  0.5502368848936002  0.4415769200719259
  0.2679628807812642  0.4449572511305935  0.5341834118657741
  0.6386486270735037  0.5947067825393980  0.5218442126004270
  0.3814394681552776  0.5034266829544731  0.5432132656343636
  0.5079027975279199  0.5565240621549074  0.5405191287016752
  0.3935528382365212  0.3699780523916340  0.5634522996876327
  0.6086349709465216  0.4641156155334542  0.5541228146856325
  0.6959133882234018  0.3711846601181619  0.5541774251613664
  0.5506043656588244  0.3430472070069425  0.5710306364357415
  0.2889641743634589  0.5822626873580242  0.5822841178203118
  0.7182113585191883  0.5082997722417801  0.6020048528060670
  0.4868242292730674  0.4581531060433968  0.6267418398683539
  0.4268455661142537  0.5869046241796548  0.6395716836036767
  0.3380265914942370  0.4715692080100970  0.6531818852900428
  0.5855062970131434  0.5595440233222536  0.6470035501185638
  0.6293419736306960  0.4212248453628730  0.6667388688297401
```

Template Cluster: Mg-10-1

```
   1.00000000000000
    20.8954140139064286    0.0000000000000000    0.0000000000000000
     0.0000000000000000   20.8954140139064286    0.0000000000000000
     0.0000000000000000    0.0000000000000000   20.8954140139064286
   Mg
    10
Direct
  0.5743470946665781  0.4798568886284187  0.4312046797296603
  0.4484251914354402  0.4195840271769882  0.3894149719662892
  0.4441435455869467  0.5586768912122224  0.4347517272777779
  0.5709308282604724  0.6208385230318925  0.4097759583933396
  0.5135453391965399  0.3761051826716368  0.5121247580933286
  0.3854599606265694  0.4524444203324620  0.5154735198894613
  0.5484147234775634  0.5798967428881809  0.5443570818359902
  0.6214111530925024  0.4558528417985386  0.5673486451142580
  0.4057255696263908  0.5834435207708424  0.5736855788734090
  0.4875965940309968  0.4733009614888175  0.6218630788264718
```

Template Cluster: Mg-15-1
1.00000000000000
18.7015475162498390 0.0000000000000000 0.0000000000000000
0.0000000000000000 18.7015475162498390 0.0000000000000000
0.0000000000000000 0.0000000000000000 18.7015475162498390
Mg
15
Direct
0.5823245721965407 0.3798245996189138 0.3635920252608358
0.3367391978625633 0.5303246491497262 0.4340870490941455
0.6596226072912543 0.3822499639123365 0.5119509254757730
0.4241944622691278 0.3810917502818043 0.3895432635333549
0.4962318156054124 0.5277259182130010 0.3988797891667559
0.4111801607053565 0.6717054820225030 0.4498549344330485
0.6577108917460066 0.5110243761618634 0.4161889446118252
0.5706167771297714 0.6599675570262207 0.4600773277512857
0.5136935036649604 0.3009041361712690 0.4956123669940808
0.4226683973053795 0.4421207763860195 0.5415864096658822
0.3437460653029945 0.5925036870258893 0.5816877129779314
0.4899882236518991 0.6729770737771759 0.5979246589539997
0.5802326128823623 0.5297999154066630 0.5590127716974393
0.5491437240280987 0.3844201168117678 0.6280201372140297
0.4619069883582796 0.5333599980348538 0.6719816831695979

Template Cluster: Mg-20-1

```
   1.00000000000000
    21.2887636785143393    0.0000000000000000    0.0000000000000000
     0.0000000000000000   21.2887636785143393    0.0000000000000000
     0.0000000000000000    0.0000000000000000   21.2887636785143393
   Mg
    20
Direct
  0.4527679211669733  0.4444085772664306  0.3741594452677816
  0.4885532249011543  0.5859860945859923  0.3783026489144015
  0.5585556699801267  0.4968961779495643  0.2937737703372416
  0.3564861670446327  0.5450163786440457  0.4090073353290626
  0.4954932549235080  0.3538448094734586  0.4783325945548844
  0.5897077353155782  0.3946442287933023  0.3829293155612777
  0.5548639776754286  0.6342459316714110  0.4956423566226179
  0.6190056241921040  0.5311103302988139  0.4173333544077329
  0.4034656346160173  0.3273425039389650  0.5834321888039656
  0.4129000378664499  0.6520499839375079  0.4799234312543931
  0.5949313226570000  0.6395082101451370  0.6328647374296001
  0.4952342049762847  0.5028399370543517  0.4990036509418403
  0.6661420109040697  0.5552961176926549  0.5471327606672561
  0.6192831918889606  0.4238342550985790  0.5193319756724422
  0.3690570718938751  0.4278318514822397  0.4893689179741177
  0.5333641015478852  0.3655429617913973  0.6166474162658002
  0.3388697517413527  0.5535822317238136  0.5477085704578051
  0.4618022056615146  0.6014326387900728  0.6031357835622871
  0.5645152011002846  0.5010199112992495  0.6326762070536677
  0.4250016899468143  0.4635668683629988  0.6192935389218245
```

Template Cluster: Mg-25-1
1.00000000000000
23.7531158613965090 0.0000000000000000 0.0000000000000000
0.0000000000000000 23.7531158613965090 0.0000000000000000
0.0000000000000000 0.0000000000000000 23.7531158613965090
Mg
25
Direct
0.5867199785670335 0.4778219038438243 0.3172814909119560
0.6553219264721464 0.5638842720656332 0.3789187425448134
0.4652891154883503 0.4551038234274506 0.3665016891909685
0.5712919987265470 0.3761858201985057 0.3859701835412895
0.5247257841453810 0.5791758394839511 0.3603413498288771
0.6714793429958574 0.4436302760463480 0.4130021855830657
0.3981511860831897 0.5599635846748524 0.3909755419695636
0.4790147267275813 0.3802032119218709 0.4721828017833880
0.5448548846480685 0.4984451493424163 0.4600051179116559
0.5951272962139560 0.6330413154202179 0.4593888150892554
0.3774219015282555 0.4558186383444357 0.4573849010595121
0.4735859920134003 0.6100725247491561 0.4758376587683544
0.6582831526568433 0.5292667194538339 0.5078079195603449
0.3581522119214083 0.5745469754310212 0.5172642427981220
0.3164744430424439 0.4699695619581602 0.5759306407888161
0.4604102074255429 0.4968510473530002 0.5492786383630033
0.3888448662652791 0.3721101732092283 0.5586400353562531
0.4448943400956472 0.6380068888201587 0.5951027155228875
0.6033780408817712 0.3929212641793715 0.5117791898255672
0.5624293028057417 0.5949273942994137 0.5725804421716822
0.5104009328199908 0.3787430179770172 0.5960038123459331
0.3657103946110886 0.5605412933990237 0.6478957590894943
0.4907588261011532 0.5407968483893706 0.6663316201628683
0.4116659925444351 0.4396687647944246 0.6618429544097412
0.5856131552188877 0.4783036912173708 0.6017515514225944

```
Template Cluster: Mg-30-1
   1.00000000000000
    25.1049362212092007    0.0000000000000000    0.0000000000000000
     0.0000000000000000   25.1049362212092007    0.0000000000000000
     0.0000000000000000    0.0000000000000000   25.1049362212092007
   Mg
    30
Direct
  0.4875631577451807  0.5508799110695826  0.3648134090849586
  0.3933841603576312  0.4860235480611033  0.3398999262181058
  0.5031990526885074  0.4274130130034077  0.3463984603881459
  0.5806224459545823  0.6231996958079038  0.3750094926142742
  0.4857532321703621  0.6320272048428945  0.4588543552994589
  0.5564786003442426  0.5347720005981602  0.4737982943476175
  0.3880469497642817  0.3726047551219054  0.3721580294064580
  0.5754373038204419  0.4120248186912334  0.4448500825708720
  0.6048233774804876  0.5017425497257831  0.3645944031288505
  0.3886311080121765  0.5627238288932692  0.4406210733187377
  0.4581868985480940  0.4645177459030473  0.4565693292026112
  0.3259230894733025  0.4533996962674079  0.4317973726650396
  0.6808091704841400  0.5855705853165240  0.4260470581221484
  0.6074611824173404  0.6585802415287164  0.4832557434201744
  0.4817684210050537  0.3406923745544428  0.4298050235764064
  0.6732743838985852  0.4779022258570204  0.4647468057561854
  0.3883470982287808  0.3713064585103377  0.5017743846348018
  0.5056970082217797  0.3782098710863697  0.5395432471799303
  0.3605576333540625  0.4884747459088170  0.5377610694219616
  0.4178690454297563  0.5978066147862787  0.5522184982808583
  0.6519242980674720  0.5637868034509919  0.5490080217675712
  0.4862621002448481  0.5000952102878845  0.5702454264165174
  0.5399052721022877  0.6172325274034469  0.5710862693201754
  0.6015895294817314  0.4501352995314967  0.5566748403221468
  0.4168197928574203  0.4099070247423955  0.6205633662460273
  0.3798007007302777  0.5251058592565445  0.6486894390158323
  0.5370519188553491  0.4111073501176892  0.6504111159160814
  0.5774145041121507  0.5271071930373936  0.6518470323366475
  0.4738710566288892  0.5926347914924817  0.6662148946126162
  0.4715275075207918  0.4830160551454561  0.7107435354087810
```

```
Template Cluster: Na-10-1
   1.00000000000000
    26.7130800960587784    0.0000000000000000    0.0000000000000000
     0.0000000000000000   26.7130800960587784    0.0000000000000000
     0.0000000000000000    0.0000000000000000   26.7130800960587784
   Na
    10
Direct
  0.4907411717957041  0.4978051280566998  0.3154272713683887
  0.5297995984258671  0.5556509678510094  0.4309656999856102
  0.5435104657005969  0.4263385313217863  0.4137608522609821
  0.4299903194605711  0.4656730822513437  0.4547465006723380
  0.6181591158592254  0.4942671122111615  0.5062304131834060
  0.4227276039510460  0.5896498036161918  0.4930074902171579
  0.5165835528063495  0.4252976570482724  0.5466379469889309
  0.4165339900453268  0.4917324504220916  0.5866534846986433
  0.5057056256527657  0.4935455952895901  0.6841416922879918
  0.5262485563025686  0.5600396719318392  0.5684286483365513
```

Template Cluster: Na-15-1

| | | |
|---|---|---|
| 1.00000000000000 | | |
| 25.4203838259235617 | 0.0000000000000000 | 0.0000000000000000 |
| 0.0000000000000000 | 25.4203838259235617 | 0.0000000000000000 |
| 0.0000000000000000 | 0.0000000000000000 | 25.4203838259235617 |
| Na | | |
| 15 | | |
| Direct | | |
| 0.4991863255170945 | 0.5687128693020902 | 0.3908348776311135 |
| 0.6222839805227983 | 0.5093460980748720 | 0.3932505385797902 |
| 0.3861817776218309 | 0.4789182994340105 | 0.4048006850154268 |
| 0.5066077037730508 | 0.4420931629704472 | 0.3281110677608024 |
| 0.6072354737924582 | 0.3728968508395397 | 0.4042145762242055 |
| 0.5709397137551282 | 0.5739099844848089 | 0.5128359905145310 |
| 0.2989130320236255 | 0.5196386311969895 | 0.5125340996840720 |
| 0.4317877332591493 | 0.5623157668001830 | 0.5159324513255483 |
| 0.5122252555731489 | 0.4504514605651336 | 0.4648109769959863 |
| 0.3987170182696169 | 0.4258851392471856 | 0.5376972530892488 |
| 0.6300301335700462 | 0.5438406162934671 | 0.6378712279580452 |
| 0.6420585196193025 | 0.4574788647022522 | 0.5234689798065569 |
| 0.4988381839808897 | 0.6015018377449984 | 0.6370639671551114 |
| 0.3804183767144603 | 0.5218600127308383 | 0.6347605042567450 |
| 0.5145767720074070 | 0.4711504056132053 | 0.6018128040028167 |

Template Cluster: Na-20-1
1.00000000000000
25.2246992318476515 0.0000000000000000 0.0000000000000000
0.0000000000000000 25.2246992318476515 0.0000000000000000
0.0000000000000000 0.0000000000000000 25.2246992318476515
Na
20
Direct

| | | |
|---|---|---|
| 0.3813977401840685 | 0.5636561444139403 | 0.3451225350690232 |
| 0.4980140818522139 | 0.4754664825446041 | 0.3474315232591966 |
| 0.4921845301520940 | 0.5904279528974694 | 0.4295912535307037 |
| 0.6063138982002289 | 0.3899062298572748 | 0.3949601727316301 |
| 0.6210735693791292 | 0.5357979228268654 | 0.4099571842609742 |
| 0.4694103265543729 | 0.3512342213591265 | 0.4189116440849241 |
| 0.3876866363272308 | 0.4644640735243134 | 0.4417741836661066 |
| 0.3642843162024924 | 0.5985256975145195 | 0.4820354102068330 |
| 0.5290996470837724 | 0.4618980147348312 | 0.4801939323944217 |
| 0.6055085076749823 | 0.6718508507119973 | 0.4598928696423834 |
| 0.4366361357632076 | 0.3877757169285519 | 0.5566026114159478 |
| 0.5681682050090272 | 0.3358014479373744 | 0.5241856628307848 |
| 0.4531886353451986 | 0.5320202626000482 | 0.5612283333381149 |
| 0.6621407788140241 | 0.4464039772803533 | 0.5181512142301138 |
| 0.4926226918877762 | 0.6613820771142045 | 0.5486187807803615 |
| 0.5995750855323611 | 0.5672882045818265 | 0.5516723640813844 |
| 0.3262344478280514 | 0.4794808227856020 | 0.5669478716799718 |
| 0.5563605412995947 | 0.4454963479245710 | 0.6164662881079866 |
| 0.4260598763586303 | 0.4638637670303724 | 0.6776923316354890 |
| 0.5240403485515572 | 0.5772597854321465 | 0.6685638330536766 |

```
Template Cluster: Na-25-1
   1.00000000000000
    25.0723134871061113    0.0000000000000000    0.0000000000000000
     0.0000000000000000   25.0723134871061113    0.0000000000000000
     0.0000000000000000    0.0000000000000000   25.0723134871061113
   Na
    25
Direct
  0.5766037165451041  0.4498862766235215  0.2873800507888796
  0.4401380254324346  0.4436104750731579  0.3396610141814282
  0.5211895902597865  0.3254111667326438  0.3271553511019474
  0.5266389259726124  0.5618458233430057  0.3738897082819977
  0.6643643866811535  0.3647509941250684  0.3641627705730209
  0.6591652910432347  0.5068839005732736  0.3911944048421871
  0.3817125932218625  0.5825574936827320  0.3948366740672509
  0.5550103894813532  0.4170080415961693  0.4238735871949450
  0.3250956832421427  0.4544048975930979  0.4457041421922001
  0.4328342773248589  0.3538741204264239  0.4580771900729619
  0.6082903098200808  0.6537395453409205  0.4531210965223185
  0.4511456352157376  0.4939456116398239  0.4735345186093528
  0.4711231032877727  0.6406746971248145  0.4885216220599582
  0.6599525379767308  0.4192930083878821  0.5170045076964884
  0.3463802802675735  0.5692973988569029  0.5415317233268632
  0.5829063532623875  0.5331043757069118  0.5081170943436567
  0.4203758822734524  0.6628902436973935  0.6305534525462274
  0.4726063841635195  0.5373675526690194  0.5984364253935224
  0.5237578708790982  0.3996502992623829  0.5640341672074484
  0.3814769194560185  0.4292587586434352  0.5778055760780718
  0.5648728653606547  0.6389622524091876  0.6013248812558880
  0.6019801837406732  0.4937694222268038  0.6379888780760625
  0.3617496349715263  0.5418295849632384  0.6828067719168800
  0.4719395030091073  0.4415695870496903  0.6964338195440051
  0.4986896571111313  0.5844144722524992  0.7228505721264377
```

Template Cluster: Na-30-1
1.00000000000000
26.3493435316983913 0.0000000000000000 0.0000000000000000
0.0000000000000000 26.3493435316983913 0.0000000000000000
0.0000000000000000 0.0000000000000000 26.3493435316983913
Na
30
Direct
0.4332629519407339 0.4484176397131361 0.5804643035116405
0.4231710535411472 0.5747460975288909 0.5299525337748460
0.5498462843042379 0.5218050700325199 0.5691909206026395
0.4555972890098821 0.5543031892376846 0.6617135217228995
0.4739423351182795 0.4724446085695309 0.4596371517235120
0.5477028830926320 0.3810096549574630 0.5323029319018157
0.5283801113268646 0.6430792051434130 0.3283155070269145
0.5347324885470263 0.4281467221048694 0.6630273099858374
0.4134807920151174 0.3545146756456470 0.4873740165559467
0.7295279823779647 0.4937849711855379 0.4412347118234768
0.5268803915069769 0.5083498101360380 0.3346546895198984
0.2978312930686839 0.6001638346860689 0.5085231375587689
0.5265692611831682 0.6521392646796033 0.5711990330266704
0.6442389139636349 0.5811348068515472 0.3686738167728426
0.3724057275558518 0.6525131812558440 0.6179410698187370
0.6510504505092423 0.4445642082712565 0.3320052139075672
0.4056504072824641 0.5670981124405012 0.3947421844137454
0.4069293641120320 0.4275356117315450 0.3625520113463506
0.5409779744206429 0.3781974143563306 0.3885955042984254
0.3251651513480076 0.5210847747911713 0.6173389300763709
0.6029995826421296 0.4801882620391042 0.4485753793869167
0.6506177721072706 0.5916056902881727 0.5093724494979682
0.3952929319092958 0.4299702672764437 0.7009430214992312
0.3203821659545270 0.3825116934219780 0.5884991666429757
0.4430692439572579 0.3259915763989737 0.6230153878699115
0.6035827387277125 0.6965309344612197 0.4364675623890901
0.6697631910206794 0.3687228746024515 0.4481947649315298
0.5279852258998069 0.5940059512471952 0.4485716988429998
0.6635281831856914 0.4529395422679501 0.5633517218793181
0.3354358583710320 0.4725003546779197 0.4835703476911252

```
Template Cluster: Rb-10-1
   1.00000000000000
    29.1167357109253295    0.0000000000000000    0.0000000000000000
     0.0000000000000000   29.1167357109253295    0.0000000000000000
     0.0000000000000000    0.0000000000000000   29.1167357109253295
   Rb
    10
Direct
  0.4647723572989728  0.5416221719449392  0.3348785418911356
  0.6302252221275867  0.4507394855486949  0.3436250359088643
  0.4852670656690873  0.4070431455829704  0.4220616710461343
  0.5870877422378638  0.5761680250750996  0.4376911682927392
  0.4165883969115845  0.5280035108344527  0.4925510176571254
  0.6040847459455050  0.4582034670554597  0.5391003155035007
  0.5416412757553488  0.6358686534422564  0.5708183210157234
  0.4792527455578422  0.3651405899159685  0.5749661705097645
  0.4717854595579767  0.5137649887912961  0.6476653356359841
  0.3192949889382182  0.5234459618088554  0.6366424225390354
```

Template Cluster: Rb-15-1
1.00000000000000
32.4706775238884120 0.0000000000000000 0.0000000000000000
0.0000000000000000 32.4706775238884120 0.0000000000000000
0.0000000000000000 0.0000000000000000 32.4706775238884120
Rb
15
Direct
0.5526800595755323 0.4647841872902440 0.3688750526819094
0.5259138578351119 0.6165967961175572 0.3760897700342235
0.4083791944003857 0.4389592227925768 0.4034419954923077
0.6371832416426090 0.5717666917702102 0.4607343305121631
0.5066589187231583 0.3349614788847406 0.4301713799071886
0.4125215710348918 0.6437428734031704 0.4738747058283621
0.4985413775014834 0.5249297603126538 0.4910844731424224
0.5953293564331729 0.4225199801488727 0.5159794451226107
0.3625348470693115 0.5074311337886789 0.5312538337820046
0.5513231001149624 0.6804525727650503 0.5161912498353312
0.3933486463625545 0.3637645926616770 0.5272065942208709
0.5192572928971179 0.3092408772631127 0.5754294540264858
0.4602537566095359 0.6075234366827723 0.6119693385725370
0.5973475816651063 0.5638304552729561 0.5995002053523887
0.4787271981350662 0.4494959408457484 0.6181981714892296

Template Cluster: Rb-20-1

```
   1.00000000000000
    32.4452068579261024    0.0000000000000000    0.0000000000000000
     0.0000000000000000   32.4452068579261024    0.0000000000000000
     0.0000000000000000    0.0000000000000000   32.4452068579261024
   Rb
    20
Direct
  0.4439636993865841  0.4365225466531562  0.3639790256447178
  0.5739703206357577  0.5084537483981998  0.3328016651938310
  0.3535645599890571  0.5087690056317564  0.4622894898764792
  0.5980603971304439  0.6084420679603785  0.4400366716203519
  0.5772487045333579  0.3741861332200768  0.3918278722028643
  0.4992584559180427  0.7197772190628843  0.4411748836019619
  0.3284532054958962  0.3666602314310070  0.4306410938559427
  0.4584848489770906  0.5878791656636191  0.3851807897279577
  0.4646939049000456  0.3547995496005953  0.4889526053061349
  0.6710677577972258  0.4766214555326201  0.4391662813386584
  0.3842459952746589  0.6490107702898512  0.4985924884045580
  0.6061097491100536  0.3865987964865210  0.5388622282904318
  0.5164159927701780  0.6367752685839144  0.5628498607513335
  0.6263172447650742  0.5330066432209357  0.5709253703200616
  0.3507695908483804  0.4077758721204154  0.5698439302432055
  0.5058615651812636  0.4974424743074045  0.4922755717429056
  0.4037584976083307  0.5438549988550925  0.6015042671812788
  0.4869395347970403  0.4198131224611964  0.6248455546136086
  0.6215350460473834  0.4340383131719687  0.6817743666043461
  0.5292809288341140  0.5495726173484354  0.6824759834793778
```

```
Template Cluster: Rb-25-1
   1.00000000000000
    35.2378105221978188    0.0000000000000000    0.0000000000000000
     0.0000000000000000   35.2378105221978188    0.0000000000000000
     0.0000000000000000    0.0000000000000000   35.2378105221978188
   Rb
    25
Direct
  0.4172558391932444  0.4464632466298897  0.3244899121280986
  0.3950322874096724  0.5763192399658107  0.3630156251916050
  0.6386636873957519  0.4669348168545926  0.3760442510810755
  0.5180570871301889  0.5261630446496801  0.3842354157420214
  0.5195691736867306  0.3863643934473880  0.3843713868852617
  0.3058659193189354  0.4748474607589661  0.3873123017091396
  0.3766933777784048  0.3519881283185645  0.4216559627998914
  0.4770196213894033  0.6358957877277367  0.4595156008347972
  0.6188587214220544  0.5783593645475446  0.4620891934549514
  0.4348479647849086  0.4547625649466595  0.4681472402313930
  0.3600358021941236  0.5641033702368115  0.4905466498875719
  0.7186399841342164  0.4969167791545971  0.4908987098586179
  0.5928721816214195  0.4258708915475276  0.4930263086145706
  0.4963402271296962  0.3255923423688176  0.5001982378880931
  0.3040838117476758  0.4365273987698199  0.5111021667197936
  0.5148460165477529  0.5307321713405193  0.5314085237250342
  0.7089627530835623  0.6139775614977286  0.5633741149394290
  0.5717867620968556  0.6458703272677996  0.5738766386119227
  0.3910129648660366  0.3580640199821344  0.5702577714789655
  0.4330670951517165  0.6261021874068942  0.5905499699825678
  0.6317838050412918  0.5077892099115154  0.5970425583678757
  0.4002292494268483  0.4927459919678322  0.6034060106796446
  0.5121950733606631  0.4166428750013970  0.6049008125465720
  0.5196344076486110  0.5481611747038756  0.6659353663112269
  0.6426461864402430  0.6128056509959116  0.6825992703298791
```

Template Cluster: Rb-30-1
1.00000000000000
36.6660245398361582 0.0000000000000000 0.0000000000000000
0.0000000000000000 36.6660245398361582 0.0000000000000000
0.0000000000000000 0.0000000000000000 36.6660245398361582
Rb
30
Direct

| | | |
|---|---|---|
| 0.3642760830247512 | 0.5810356819240039 | 0.3516274425800372 |
| 0.3400911515082507 | 0.4502538255655857 | 0.3457290685376461 |
| 0.4709582300707582 | 0.4934067304704487 | 0.3446768878926398 |
| 0.5768136022544404 | 0.4027734901485188 | 0.3894112197138279 |
| 0.4338929996390624 | 0.3816636750003964 | 0.4190376688837227 |
| 0.4769448113098427 | 0.6063228025085469 | 0.4209535541623338 |
| 0.5946253736178067 | 0.5392451750701590 | 0.3908504640390995 |
| 0.2684185284636158 | 0.5316121956663280 | 0.4303758156040459 |
| 0.6967225925181486 | 0.4561733688756537 | 0.4107671217579126 |
| 0.3900854753613393 | 0.5054280580691621 | 0.4533053774626364 |
| 0.3520290820097887 | 0.6291956610869173 | 0.4804290637358888 |
| 0.5746165937415363 | 0.6082314229664866 | 0.5087235980886020 |
| 0.6634897944119732 | 0.3524362266267724 | 0.4766468583476738 |
| 0.3060904071186460 | 0.4085784318834847 | 0.4678674670009771 |
| 0.5094962986562296 | 0.4762372917426571 | 0.4687032920248307 |
| 0.6976948157538088 | 0.5673117068538754 | 0.4743686565728960 |
| 0.5342796232159840 | 0.3541564096369240 | 0.5099476406429027 |
| 0.4607951238811975 | 0.6747461151046377 | 0.5395882710506251 |
| 0.6108954384939725 | 0.4810114931030328 | 0.5275536828689450 |
| 0.4546913403346830 | 0.5490076716809571 | 0.5509978072423968 |
| 0.6340179749469599 | 0.3701320812039121 | 0.5996641746081057 |
| 0.4117341367981239 | 0.4111028992181988 | 0.5499132664682658 |
| 0.5196909842660553 | 0.4410494036773866 | 0.6156903542868175 |
| 0.7276062208953890 | 0.4568874283927731 | 0.5492249195105939 |
| 0.6710148784708260 | 0.5874786656579122 | 0.5990088962707155 |
| 0.5440785069900697 | 0.5723034087415381 | 0.6353957288275680 |
| 0.3116424265595486 | 0.5105728831877652 | 0.5592747851222299 |
| 0.3634425339639875 | 0.6204664610131587 | 0.6118986222262729 |
| 0.6374275476838317 | 0.4822057895400933 | 0.6637378808178945 |
| 0.4024374240393659 | 0.4989735453827279 | 0.6546304136519034 |

Template Cluster: Sr-10-1
1.00000000000000
26.3713431732858083 0.0000000000000000 0.0000000000000000
0.0000000000000000 26.3713431732858083 0.0000000000000000
0.0000000000000000 0.0000000000000000 26.3713431732858083
Sr
10
Direct
0.5192982997588231 0.4107492580269086 0.3885567216955499
0.4441991997888224 0.5557832695989787 0.3841199922806940
0.6035577456788691 0.5482594417642489 0.4179639449018810
0.4060855734819948 0.3961952256406197 0.5069309629031786
0.5012136869400805 0.4986276424067876 0.4984685712422091
0.3676359365295486 0.5513618982144418 0.5256389422958246
0.6173561848388166 0.5306266489022251 0.5772554900957644
0.5640003210568296 0.3824581626682039 0.5431984599220141
0.5056417867373674 0.6348640382051218 0.5241097003396292
0.4710112651888477 0.4910744145724495 0.6337572143232550

Template Cluster: Sr-15-1

```
   1.00000000000000
    28.8972837448572584    0.0000000000000000    0.0000000000000000
     0.0000000000000000   28.8972837448572584    0.0000000000000000
     0.0000000000000000    0.0000000000000000   28.8972837448572584
   Sr
    15
Direct
  0.4959329243777900  0.5160017508054220  0.3449694377953686
  0.5624981266420112  0.4067591479927680  0.4131225955144108
  0.6287112864943528  0.5364110132463905  0.4035301003793234
  0.4183336549781480  0.4272667847593996  0.4361014361574176
  0.5260315082047761  0.6401905171160260  0.4171051182500810
  0.3975879111251825  0.5732664011605542  0.4350578166926091
  0.5171767917869959  0.5192459288217787  0.4802071278925098
  0.3652371309293619  0.3608403103028870  0.5557394093976642
  0.5027990580987025  0.3959243266699804  0.5477610282783894
  0.4032548849069834  0.4972436804136279  0.5607116576672123
  0.6335354642694692  0.4619749343634685  0.5291108465452381
  0.4691778339451261  0.6284848754168996  0.5511552507421360
  0.6109924486342956  0.6049532432387186  0.5318610486673546
  0.5318087835288221  0.5154431218111352  0.6215054335765455
  0.4369221920779828  0.4159939638809652  0.6720616924437538
```

```
Template Cluster: Sr-20-1
   1.00000000000000
    25.5002255720189410    0.0000000000000000    0.0000000000000000
     0.0000000000000000   25.5002255720189410    0.0000000000000000
     0.0000000000000000    0.0000000000000000   25.5002255720189410
   Sr
    20
Direct
  0.5281179701555473  0.5389796749737199  0.3175955298398968
  0.3719432876852287  0.4860844700834978  0.3507670891766052
  0.5051593186617376  0.4052832906385173  0.4068786520998615
  0.4013273214627328  0.6489969866339284  0.3534395004553084
  0.6206077261760913  0.5242612948350834  0.4500188234040459
  0.6477797356165906  0.4172645711617208  0.3317912219518389
  0.5521557112241027  0.6717041793404783  0.4181437253900334
  0.4604228406589475  0.5534763689608156  0.4589315760759234
  0.3719371797175842  0.4267187722355448  0.5076938849109419
  0.3082553614016423  0.5767023433836472  0.4732578005096258
  0.6358924795075253  0.3694134401440758  0.4960213728619829
  0.4856941146823261  0.3067101876245124  0.5387438057618398
  0.4199337418731254  0.6917058399788376  0.5151181429730606
  0.5220111485382160  0.4565079787726910  0.5611902651792893
  0.5543409093791847  0.6147236953826086  0.5760975567225026
  0.4037237074732785  0.5536665175110408  0.6094374456165673
  0.6662326733368008  0.4927362745510838  0.6063630262612055
  0.4231294099593567  0.3963763093323228  0.6633287641736968
  0.5866716525838769  0.3577377977116156  0.6601913919944988
  0.5346637099060905  0.5109500067442653  0.7049904246412608
```

```
Template Cluster: Sr-25-1
   1.00000000000000
    26.9950687165133516    0.0000000000000000    0.0000000000000000
     0.0000000000000000   26.9950687165133516    0.0000000000000000
     0.0000000000000000    0.0000000000000000   26.9950687165133516
   Sr
    25
Direct
  0.5693581727564008  0.3570434853827095  0.3449563585490024
  0.4912672332530312  0.4964975012762364  0.3239580410507139
  0.4125524353816047  0.3590228038497752  0.3591613393653095
  0.5731957525194418  0.6339102348759531  0.3457666463166791
  0.4175946255496420  0.6358566036332567  0.3597616960808450
  0.6273319022779638  0.4950539886777947  0.4053270943924037
  0.3705520755493406  0.4977468165025187  0.4262057988077360
  0.4992862957154526  0.2779916613804263  0.4622351596167520
  0.5014808564347073  0.4218453696491179  0.4544915237401567
  0.5031783578107208  0.5697677219485735  0.4544752241345053
  0.6339249468437674  0.3641048575592978  0.4901213229070538
  0.3750679090172344  0.3685079906731351  0.5161355829009120
  0.5048941097034166  0.7138607372190026  0.4630892370017721
  0.6367327536330880  0.6240373747911434  0.4957458691661097
  0.3790879651942348  0.6272596363696281  0.5167940804494536
  0.7299945617573126  0.4990821089607875  0.5255500059688959
  0.4372242169983522  0.4966553815335686  0.5649088829723202
  0.3802711338979441  0.5772269283488672  0.6702131393541272
  0.5864670495961809  0.4939247649174241  0.5545661162495693
  0.6581975077022006  0.5819121108203541  0.6476057627229012
  0.5156019883968842  0.6249032380147341  0.5966675833441002
  0.2924044596270130  0.4988484324446256  0.5655643304271902
  0.5172566454887276  0.4994524526795313  0.6901729548737122
  0.5093558136401455  0.3663316571652352  0.5959308334932707
  0.3777212312552067  0.4191561413263105  0.6705954161145011
```

Template Cluster: Sr-30-1
1.00000000000000
32.8327787031128508 0.0000000000000000 0.0000000000000000
0.0000000000000000 32.8327787031128508 0.0000000000000000
0.0000000000000000 0.0000000000000000 32.8327787031128508
Sr
30
Direct
0.4090123393718823 0.4765946402613128 0.3222074565878123
0.5319352569996245 0.5167916214518031 0.2986699031813244
0.4400870004304320 0.6038525089584761 0.3337598868625898
0.6083831169650601 0.3640468554280822 0.4530356462891743
0.5127851701501408 0.4040226844173121 0.3692034734837970
0.6065318766402901 0.4897593938262054 0.4034303124041348
0.3617011950881563 0.5463484648404042 0.4274926115112932
0.5632198444130628 0.6133226814730145 0.3866210829361957
0.4820857318856976 0.5199078908602477 0.4056940152020582
0.5533083762051708 0.5586918673580142 0.4952909646853721
0.4073528475323547 0.4249000111272536 0.4434114852180078
0.6647151397845756 0.5883437172210919 0.4719108542231416
0.5254513344707558 0.4398639359825257 0.4850259457955293
0.4559707875634834 0.6310271465749311 0.4617055653931658
0.4831469265380729 0.3274464553962300 0.4788454594466173
0.3516970547759741 0.3364893129607924 0.5127606129351264
0.5736861600490590 0.6742905619447961 0.5069187784761542
0.4389090700642506 0.5209627815080354 0.5183888876799617
0.3322626768815307 0.4691082032344182 0.5373393962771201
0.6376817684216964 0.4717753600753000 0.5297126654198107
0.3578646448300192 0.5991469484258481 0.5500333312220995
0.4392423801946586 0.4062612698495385 0.5700781297609824
0.4870040011042701 0.6129243810829160 0.5880965185782047
0.5653735355887475 0.3736791891257794 0.5792920068634997
0.5290916022981810 0.4925067343247768 0.5935970298946384
0.6151271985004864 0.5829853493621199 0.5975619683568228
0.4141105379573247 0.5154641601079226 0.6373996022088373
0.6224801581948427 0.4648906946372676 0.6606614155309841
0.5000188472221390 0.4232051696632032 0.6856974166889204
0.5297634198780746 0.5513900085203671 0.6961575768866385

```
Template Cluster: Zn-10-1
   1.00000000000000
    15.8392282053775801    0.0000000000000000    0.0000000000000000
     0.0000000000000000   15.8392282053775801    0.0000000000000000
     0.0000000000000000    0.0000000000000000   15.8392282053775801
   Zn
    10
Direct
  0.3948466698515887  0.3773261333392739  0.3971789342066145
  0.5499591127807832  0.4333309736188369  0.4126305639092109
  0.5500739112835831  0.5971269939075938  0.3741416064839314
  0.4073504623712623  0.5350053316133909  0.4343962709422513
  0.6367392973836914  0.5450903654603891  0.5040842851983527
  0.4963686270314582  0.6427894147462041  0.5261700888565102
  0.6113287244636112  0.3934446643229492  0.5649476859625153
  0.5316819212890138  0.5191579275739926  0.6391356743206202
  0.4460237430661422  0.4054035945230083  0.5504974316814639
  0.3756275304788872  0.5513246008943824  0.5968174584385295
```

```
Template Cluster: Zn-15-1
   1.00000000000000
    18.5841230912179292    0.0000000000000000    0.0000000000000000
     0.0000000000000000   18.5841230912179292    0.0000000000000000
     0.0000000000000000    0.0000000000000000   18.5841230912179292
   Zn
    15
Direct
  0.4500649743665193  0.5237672717129414  0.3249144096036289
  0.5870674897911172  0.5317492187024513  0.3623900676941290
  0.4936225011460706  0.6444745798343376  0.3775505315649568
  0.4867714948951232  0.4459601872493764  0.4366990525613517
  0.3999098882082137  0.5664558772332811  0.4563622044434794
  0.5999009252977956  0.6257537595915821  0.4745509639354637
  0.4367499466555458  0.3273010347983032  0.4929714951006616
  0.6108921533744720  0.4846110588599970  0.4959360108666431
  0.4737090848945584  0.6813308236414528  0.5112645443542050
  0.5724662529442242  0.3505442675515766  0.5254972767156971
  0.3891717956577239  0.4597062497422186  0.5448860301354301
  0.5051497818617605  0.5538654298285621  0.5621475699413168
  0.4629638022618678  0.3629669378826534  0.6249070986597829
  0.5809395287287700  0.4495642437669716  0.6341167946863919
  0.4506203799162307  0.4919490596042451  0.6758059497368976
```

```
Template Cluster: Zn-20-1
   1.00000000000000
    22.1391528277089193    0.0000000000000000    0.0000000000000000
     0.0000000000000000   22.1391528277089193    0.0000000000000000
     0.0000000000000000    0.0000000000000000   22.1391528277089193
   Zn
    20
Direct
  0.4973740536378557  0.4152871915622711  0.3468269886408564
  0.5485005207771394  0.5872863135188737  0.4311888732706990
  0.4647639446731802  0.5204380724336275  0.3844684786360304
  0.3632563951322894  0.5258856450943341  0.4457034976870276
  0.5361591637435481  0.3646524412218781  0.4414622048939094
  0.5802352859214821  0.4740811735173409  0.4054480433970511
  0.4316042613431819  0.6206547326415119  0.4416342955978720
  0.4255620933835758  0.4226944841995177  0.4421308339186254
  0.6485128934582676  0.5282239018312964  0.4811057571307273
  0.4982436316272540  0.5011367602097039  0.4994348919834195
  0.6152386912915588  0.6305786922636614  0.5199595220221127
  0.5919722032838806  0.4318809044365395  0.5206615536120864
  0.5016557924137786  0.6160420508762172  0.5377174388481762
  0.3859042614357691  0.5863875521596000  0.5433222858291842
  0.4813476478315053  0.3810815315896505  0.5418990277648845
  0.3913216947172700  0.4675200008718861  0.5457958887192760
  0.5862613401185257  0.5371170710986419  0.5860585969253407
  0.4687473104258402  0.5315985032260997  0.6127202647410783
  0.5478502173583593  0.4367553321279445  0.6299825975373281
  0.4354885974257670  0.4206976451193967  0.6424789588442650
```

Template Cluster: Zn-25-1

```
   1.00000000000000
    22.2251049038818707    0.0000000000000000    0.0000000000000000
     0.0000000000000000   22.2251049038818707    0.0000000000000000
     0.0000000000000000    0.0000000000000000   22.2251049038818707
   Zn
    25
Direct
  0.4132166296646673  0.5201257268356798  0.3321666042393474
  0.5141734726550951  0.5648108009390498  0.3607202934305604
  0.4109512144211180  0.6250610849755702  0.3862791419247884
  0.6050226631257729  0.4970302942736566  0.3914314752496466
  0.3633494262608608  0.4414245502056744  0.3942835035079355
  0.4919802091523380  0.4568219669372695  0.4148156198246653
  0.3314275186496261  0.5471432716577240  0.4289015967765764
  0.5983780737647990  0.5974811486111662  0.4414791039163788
  0.5976327909492127  0.4126371828831523  0.4674709145308569
  0.4866700153764825  0.5883208177516295  0.4742316944630289
  0.5011454257829087  0.3515636695445465  0.4751488442039076
  0.3919878375964743  0.3797085580119131  0.4834226397201675
  0.3752745344883266  0.6253220428811659  0.5005590574872798
  0.6471031405634591  0.5149477804010516  0.5072255114013871
  0.4033688473094230  0.4978535112604234  0.5075763825871604
  0.5237981176454198  0.4877096757078121  0.5253525706516697
  0.5690849922514545  0.5974130918958096  0.5547704290820690
  0.6554234539281794  0.4192641783364905  0.5705962618487774
  0.5676803450516701  0.3480265655362018  0.5739635964131580
  0.4560098667556929  0.3975022415784935  0.5756511273837788
  0.4518840495343568  0.6101638670061291  0.5883744245969424
  0.4353857502809788  0.5013388270999144  0.6203990054720236
  0.6325102550997102  0.5194972413645402  0.6252541690458528
  0.5460037172083005  0.4411715843187665  0.6421364016401583
  0.5305376524836518  0.5576603199861765  0.6577896306018971
```

```
Template Cluster: Zn-30-1
   1.00000000000000
    23.2068300044197287    0.0000000000000000    0.0000000000000000
     0.0000000000000000   23.2068300044197287    0.0000000000000000
     0.0000000000000000    0.0000000000000000   23.2068300044197287
   Zn
    30
Direct
  0.5170378785953247  0.5756902067709078  0.3547201715144865
  0.5113845932816332  0.4634141341182598  0.3621171731154078
  0.6081989829045398  0.5217492358169267  0.3759018500580211
  0.4212461994754690  0.5294715959202492  0.3829830261586622
  0.4138639920853890  0.4209606397711667  0.3931731938820704
  0.6045036293250163  0.4213954558464721  0.4150699704882754
  0.5036828224146959  0.3646125826303575  0.4198217801559565
  0.4555665188689488  0.6374156879807202  0.4225025410416805
  0.5860641533415247  0.6066844732837851  0.4399461580440445
  0.3577377169890710  0.5912606837404653  0.4486412907423087
  0.6046485518188334  0.3157129082384031  0.4560366601806578
  0.3611468803175626  0.4782372798607781  0.4687254460751191
  0.6424179476567886  0.5168057264748782  0.4802571105024498
  0.5236640379685653  0.4566163813038173  0.4923054083025988
  0.4176038007904168  0.3837020452246591  0.4963800834958931
  0.5235181014878521  0.6696101275760924  0.5027286024002606
  0.4761697894619061  0.5442561060822939  0.5035928635699705
  0.6271651893576831  0.4085904076814018  0.5242179738138404
  0.5273420110651799  0.3471889985242953  0.5299144399051130
  0.4198024122074457  0.6374324306201231  0.5335809926628556
  0.3445835507133381  0.5551640572352170  0.5505033613605477
  0.5960698888095806  0.5943142969432103  0.5497109473173651
  0.3822071915327356  0.4561076330817144  0.5756616569325672
  0.6332595303979360  0.4985612738934790  0.5873378568984862
  0.4511891782938733  0.3753995898044522  0.6012407421930774
  0.5078969523792480  0.6379074535957159  0.6064236270407619
  0.5545223879206702  0.4245749483035581  0.6120848142222357
  0.4259322904799134  0.5649732666857332  0.6233899746474805
  0.4606400385717393  0.4663822046907597  0.6584231410803270
  0.5409337814871193  0.5358081683000933  0.6326071421974646
```

# REFERENCES


1 Holland, J. H. *Adaptation in Natural and Artificial Systems: An Introductory Analysis with Applications to Biology, Control, and Artificial Intelligence*. 1 edn, (Ann Arbor, MI: University of Michigan Press, 1975).

2 Johnston, R. L. Evolving better nanoparticles: Genetic algorithms for optimising cluster geometries. *Dalton Trans.*, 4193-4207 (2003).

3 Shayeghi, A., Götz, D., Davis, J. B. A., Schäfer, R. & Johnston, R. L. Pool-BCGA: a parallelised generation-free genetic algorithm for the ab initio global optimisation of nanoalloy clusters. *Physical Chemistry Chemical Physics* **17**, 2104-2112 (2015).

4 Wang, Y. *et al.* Accelerated prediction of atomically precise cluster structures using on-the-fly machine learning. *npj Comput. Mater.* **8**, 173 (2022).
